# Estimation of Recombination Rate and Maternal Linkage Disequilibrium in Half-Sibs

**DOI:** 10.3389/fgene.2018.00186

**Published:** 2018-06-05

**Authors:** Alexander Hampel, Friedrich Teuscher, Luis Gomez-Raya, Michael Doschoris, Dörte Wittenburg

**Affiliations:** ^1^Leibniz Institute for Farm Animal Biology (FBN), Institute of Genetics and Biometry, Dummerstorf, Germany; ^2^Departamento de Mejora Genética Animal, Instituto Nacional de Investigación y Tecnología Agraria y Alimentaria (INIA), Madrid, Spain

**Keywords:** allele frequency, expectation maximization algorithm, genome assembly, likelihood function, linkage analysis

## Abstract

A livestock population can be characterized by different population genetic parameters, such as linkage disequilibrium and recombination rate between pairs of genetic markers. The population structure, which may be caused by family stratification, has an influence on the estimates of these parameters. An expectation maximization algorithm has been proposed for estimating these parameters in half-sibs without phasing the progeny. It, however, overlooks the fact that the underlying likelihood function may have two maxima. The magnitudes of the maxima depend on the maternal allele frequencies at the investigated marker pair. Which maximum the algorithm converges to depends on the chosen start values. We present a stepwise procedure in which the relationship between the two modes is exploited. The expectation maximization algorithm for the parameter estimation is applied twice using different start values, followed by a decision process to assess the most likely estimate. This approach was validated using simulated genotypes of half-sibs. It was also applied to a dairy cattle dataset consisting of multiple half-sib families and 39,780 marker genotypes, leading to estimates for 12,759,713 intrachromosomal marker pairs. Furthermore, the proper order of markers was verified by studying the mean of estimated recombination rates in a window adjacent to the investigated locus as well as in a window at its most distant chromosome end. Putatively misplaced markers or marker clusters were detected by comparing the results with the revised bovine genome assembly UMD 3.1.1. In total, 40 markers were identified as candidates of misplacement. This outcome may help improving the physical order of markers which is also required for refining the bovine genetic map.

## Introduction

Population genetic parameters, such as linkage disequilibrium (LD) and recombination rate, are relevant parameters for characterizing a livestock population. Methods for genomic selection (e.g., Meuwissen et al., 2001) implicitly exploit the LD between quantitative trait loci (QTL) and genetic markers for estimating breeding values or genetic effects captured by the markers in a population. The accuracy of estimated effects depends on the extent of LD (e.g., Hayes et al., 2009), which is affected by several factors (e.g., Ardlie et al., 2002). For instance, recombination breaks the physical linkage between chromosomal segments. Hence a recombination event creates new combinations of alleles in the next generation. Furthermore, population structure influences the extent of LD differing not only between populations and breeds (De Roos et al., 2008) but also between families within a breed (Dekkers, 2004).

The estimation of recombination rate requires genotypic and pedigree information—most often parent-offspring data are used. Different approaches are available enabling the parameter estimation based on phased (e.g., “direct method”; Ott, 1991) or unphased genotype data (e.g., likelihood approaches such as the LOD score; Ott, 1991). Such approaches are applicable to natural populations and experimental crosses (e.g., F2 or backcross) but typically not to livestock populations. Non-random mating influences the population structure and the way of data collection in livestock. For instance, in paternal half-sib families, the sire and its ancestors are genotyped but not necessarily the dam. Paternal half-sib families are a typical family structure in dairy cattle. In this context, the paternal recombination rate (θ) and the LD of maternal gametes (*D*^*dam*^) are of particular interest, for instance, to map QTL in such breeds. Neglecting the population structure may lead to biased parameter estimates.

Maps for cattle breeds are available in low dimensions, and they have mostly been based on the analysis of microsatellites (e.g., Kappes et al., 1997; Thomsen et al., 2001). Due to advances in molecular genetics, single nucleotide polymorphisms (SNPs) are available to cover animal genomes at high density. With the exception of some errors, the order of SNPs is known from the genome sequence (Bovine HapMap Consortium, 2009). In a recent effort, estimates of recombination rates considering more than 50 K SNPs have been determined (Ma et al., 2015) which opens up the way to a more comprehensive map.

Previous studies (Gomez-Raya, 2012; Gomez-Raya et al., 2013) explicitly exploited the family structure of paternal half-sibs and proposed a likelihood approach for the estimation of θ and *D*^*dam*^ which was based on an expectation maximization (EM) algorithm. However, a special characteristic of the underlying likelihood function (LF) is its bimodality (Figure 2 in Gomez-Raya et al., 2013) and thus the possibility of having two maxima. Depending on the maternal allele frequencies at the two loci, the modes can have equal height. EM converges to a local maximum—which one depends on the start values (Dempster et al., 1977). Specifying alternative start values allows identifying the two modes and the selection of the most likely estimate. However, in case of equal-height modes, a unique estimation of *D*^*dam*^ and θ is actually not possible. Therefore, the first objective of this study was to elucidate the relationship between the two modes of LF which is incorporated in determining alternative start values. In case of equally high modes, the most likely estimate of *D*^*dam*^ and θ is selected based on additional information from the neighborhood. A stepwise procedure is proposed which was validated using simulated genotypes of half-sibs. The second objective addresses the proper order of SNPs according to the underlying genome assembly. As recombination rates can be estimated between all pairs of SNPs on a chromosome, and not only between adjacent SNPs, the pattern of estimates of θ will be examined in depth to identify SNPs which are putatively misplaced in the underlying genome assembly. Empirical data consisting of multiple half-sib families of Holstein-Friesian cows were analyzed, and candidates for misplacement in the genome assembly are presented.

## Methods

At first, the LF for estimating θ and *D*^*dam*^ is inspected to differentiate its bimodality. Then, the unknown parameters are estimated using an EM algorithm corresponding to the LF and two different sets of start values. A decision process (DP) is employed to find the most probable final estimate when the LF has two modes.

### Likelihood function

The SNP genotypes of progeny are used to estimate the unknown parameters; SNP alleles are denoted as A and B. The observed genotype frequencies at two loci, e.g., AA and AB, are denoted as *n*_*AA, AB*_. It is assumed that paternal linkage phases are known without error, and only those loci at which a sire is heterozygous are considered. Otherwise a recombination rate cannot be inferred. The sire haplotypes are denoted as A-A and B-B in the coupling phase as well as A-B and B-A in the repulsion phase. Hence the frequency of the paternal A allele is 0.5 among offspring.

Based on the multinomial distribution and considering a full model, in which the maternal haplotype frequencies and θ are the unknown parameters, the logarithmic LF can be expressed similarly to Gomez-Raya (2012) as:

$$\begin{array}{l} \log LF(\pi_{AA,AA},\pi_{AA,AB},\ldots ,\pi_{BB,BB}|n_{AA,AA},n_{AA,AB},\ldots ,n_{BB,BB})\\ \;=\sum_{i,k\in \{AA,AB,BB\}}n_{i,k}\log\pi_{i,k}+\text{constant}, \end{array}$$

where π_*i, k*_ is the probability of the two-locus genotype *i, k*∈{*AA, AB, BB*}. For instance, $\pi_{AA,AB}=\frac{1}{2}\left(1-\theta \right)p_{A-B}+\frac{1}{2}\theta p_{A-A}$ in the coupling phase. If the sire haplotypes are in repulsion phase, θ changes to 1−θ. The log*LF* depends on the frequency of maternal gametes (e.g., *p*_*A*−*A*_ denotes the frequency of the maternal haplotype A-A) and θ. It is now reparametrized and written in terms of LD of maternal and paternal gametes, $D^{dam}=p_{A-A}-p_{1}p_{2}$ and *D*^*sire*^ = ±(1−2θ)/4 (the positive sign corresponds to the coupling phase), respectively, to better investigate the relationship between these parameters. Indices are used at frequencies (*p*_1_ and *p*_2_) of the maternal A allele to distinguish between the loci. Hence, using the computer algebra system *Maple*™ (Maplesoft, Waterloo, Ontario, Canada):

(1) $$\begin{array}{l} \log LF\left(D^{sire},D^{dam},p_{1},p_{2}|n_{AA,AA},n_{AA,AB},\ldots ,n_{BB,BB}\right)\\ =n_{BB,BB}\log\left(\frac{1}{4}\left(1+4D^{sire}\right)\left(D^{dam}+\left(-1+p_{1}\right)\left(-1+p_{2}\right)\right)\right)\\ +\;n_{BB,AB}\log\left(\frac{1-p_{1}}{4}+D^{sire}\left(-2D^{dam}+\left(-1+p_{1}\right)\left(1-2p_{2}\right)\right)\right)\\ +\;n_{BB,AA}\log\left(\frac{1}{4}\left(-1+4D^{sire}\right)\left(D^{dam}+\left(-1+p_{1}\right)p_{2}\right)\right)\\ +\;n_{AB,BB}\log\left(\frac{1-p_{2}}{4}+D^{sire}\left(-2D^{dam}+\left(1-2p_{1}\right)\left(-1+p_{2}\right)\right)\right)\\ +\;n_{AB,AB}\log\left(\frac{1}{4}+D^{sire}\left(4D^{dam}+\left(-1+2p_{1}\right)\left(-1+2p_{2}\right)\right)\right)\\ +\;n_{AB,AA}\log\left(\frac{p_{2}}{4}+D^{sire}\left(-2D^{dam}+p_{2}\left(1-2p_{1}\right)\right)\right)\\ +\;n_{AA,BB}\log\left(\frac{1}{4}\left(-1+4D^{sire}\right)\left(D^{dam}+p_{1}\left(-1+p_{2}\right)\right)\right)\\ +\;n_{AA,AB}\log\left(\frac{p_{1}}{4}+D^{sire}\left(-2D^{dam}+p_{1}\left(1-2p_{2}\right)\right)\right)\\ +\;n_{AA,AA}\log\left(\left(\frac{1}{4}+D^{sire}\right)\left(D^{dam}+p_{1}p_{2}\right)\right)+\text{constant}. \end{array}$$

**Figure 1:**
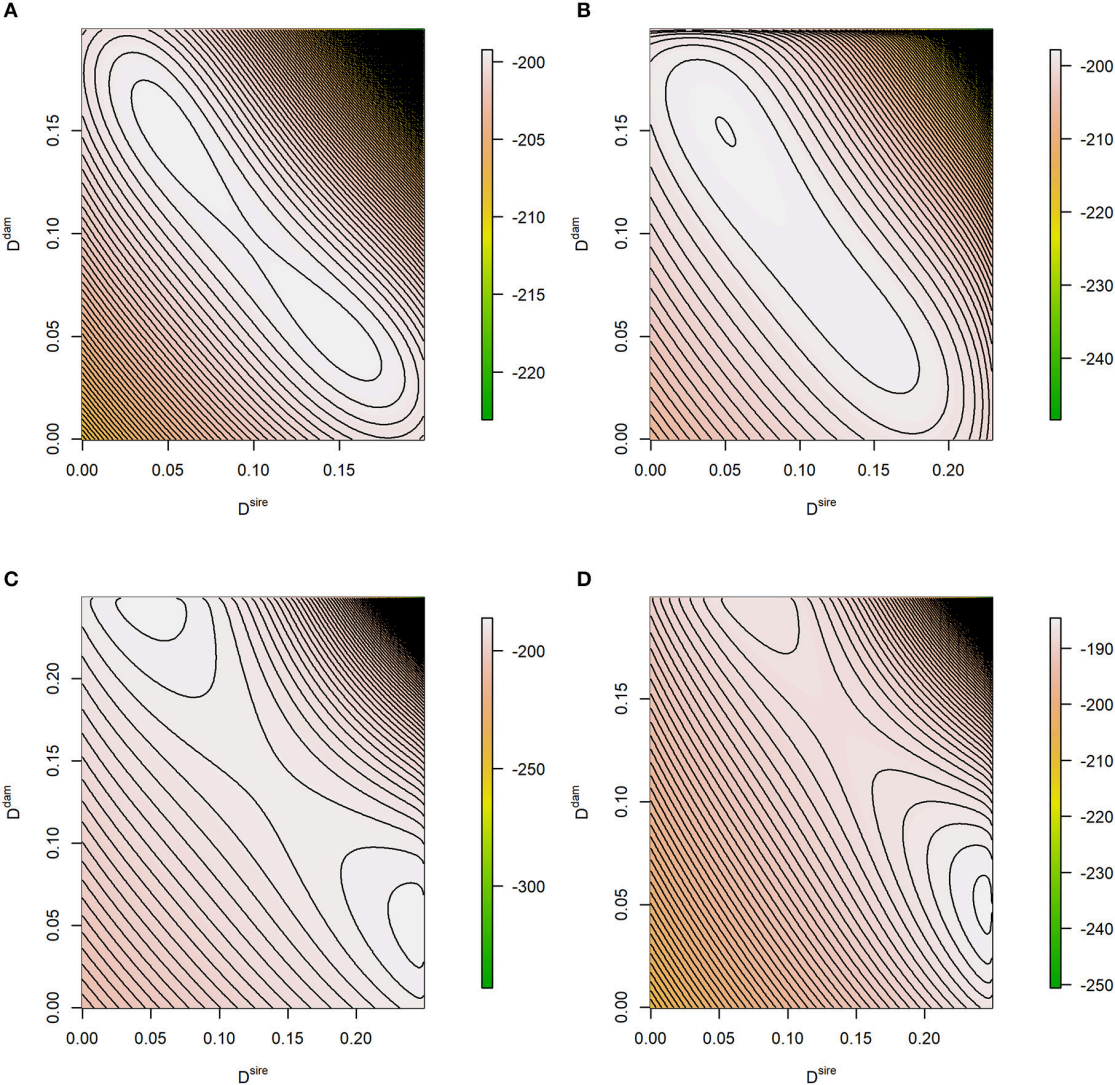
Contour plot of the theoretical log-likelihood function. The true parameters of the likelihood problem were **(A)** *p*_1_ = *p*_2_ = 0.5, *D*^*sire*^ = 0.05, *D*^*dam*^ = 0.15; **(B)** *p*_1_ = 0.5, *p*_1_ = 0.4, *D*^*sire*^ = 0.05, *D*^*dam*^ = 0.15; **(C)** *p*_1_ = *p*_2_ = 0.5, *D*^*sire*^ = 0.245, *D*^*dam*^ = 0.05, and **(D)** *p*_1_ = 0.5, *p*_1_ = 0.4, *D*^*sire*^ = 0.245, *D*^*dam*^ = 0.05. Expected genotype frequencies based on the true parameters were considered, e.g., $n_{AA,AA}=\left(1/4+D^{sire}\right)\left(D^{dam}+p_{1}p_{2}\right)$.

The shape of log*LF* depends on the maternal allele frequencies at the two loci: they affect the number (one or two) and height of the modes. Plotting this function also reveals its characteristics, see Figure 1. The examples consider maternal and paternal LD being inside (Figures 1A,B) or near the boundary (Figures 1C,D) of the parameter space which is *D*^*sire*^∈[−0.25, 0.25] and $D^{dam}\in \left[L_{1},L_{2}\right]$ with *L*_1_ = max{−*p*_1_*p*_2_, −(1−*p*_1_)(1−*p*_2_)} and *L*_2_ = min{*p*_1_(1−*p*_2_), (1−*p*_1_)*p*_2_} (Lewontin, 1964). In absence of distorted segregation, *D*^*sire*^ is not restricted by allele frequencies and has its extreme value of ±0.25. In case of maternal allele frequencies being 0.5 at both loci, the modes have equal height, and a symmetric pattern in terms of *D*^*dam*^ and *D*^*sire*^ is observed (Figures 1A,C). For frequencies beyond 0.5, the log*LF* is either unimodal or the two modes have different heights.

### Start values for the EM algorithm

Although the form of log*LF* is elementary, computing the maximum-likelihood (ML) estimation is not straightforward, see e.g., Hill (1974) and File S1. Gomez-Raya (2012) and Gomez-Raya et al. (2013) proposed an EM algorithm to estimate θ and maternal haplotype frequencies based on the log*LF*. For an EM algorithm, and for other commonly used optimization methods, it is known that it may converge to a local maximum if the underlying distribution is bimodal (Dempster et al., 1977). Which maximum is found depends on the start values of the unknown parameters. Thus, an EM algorithm shall be started multiple times with different start values to explore the modes of the likelihood surface (e.g., Biernacki et al., 2003). In our experience, there will be no more than two modes. Thus, we will use two sets of start values thereby employing the relationship between the two modes.

The theoretical derivations of Bonk et al. (2016) can be adapted and applied to estimate the statistical dependence in half-sib families. The covariance between genotype codes for additive effects cov_*add*_ (AA, AB and BB are coded as 1, 0, and −1, respectively) and dominance effects cov_*dom*_ (AA, AB, and BB are coded as −1, 1, and −1, respectively) require the paternal and maternal LD, and a system of two equations is achieved:

$$\begin{array}{l} {\mathrm{cov}}_{dom}=16D^{sire}D^{dam}+4D^{sire}\left(1-2p_{1}\right)\left(1-2p_{2}\right),\\ {\mathrm{cov}}_{add}=D^{sire}+D^{dam}. \end{array}$$

Then two solutions for *D*^*dam*^ and *D*^*sire*^ (indices *I* and *II*) can be obtained. Given one solution, the other solution is derived as:

(2) $$\begin{array}{l} D_{II}^{sire}=D_{I}^{dam}+\frac{1}{4}(1-2p_{1})(1-2p_{2})\text{ and}\\ D_{II}^{dam}=D_{I}^{sire}-\frac{1}{4}(1-2p_{1})(1-2p_{2}). \end{array}$$

In the particular case of *p*_1_ = *p*_2_ = 0.5, maternal and paternal LD are mutually exchangeable.

For the first run of EM, default start values are used: *D*^*sire*^ = 0.25 (θ = 0) and *D*^*dam*^ = 0. Start values for the maternal haplotype frequencies are received from *D*^*dam*^ and ML estimates of the maternal A allele at the investigated loci, i.e., $\hat{p}=n_{AA}/\left(n_{AA}+n_{BB}\right)$, where *n*_*AA*_ and *n*_*BB*_ denote the number of offspring with AA and BB genotypes, respectively. Start values for the second run are obtained after using the EM estimates of the first run and employing the complementary relationship in Equation (2). If this relationship leads to start values outside the parameter space, we conclude that the underlying likelihood is unimodal and skip a second EM run. Otherwise, this “educated guess” shall lead the EM algorithm to the second mode.

### Decision process for non-critical allele frequencies

In our experience, log*LF* can either be unimodal or bimodal with modes of different height when *p*_1_ and *p*_2_ differ from 0.5. Then, the most likely estimate of *D*^*dam*^ and θ can be identified from comparing the value of log*LF* at the two proposals. It is obvious to select the estimates of parameter values constituting the higher value of log*LF*. Because log*LF* can be rather flat (e.g., Figure 1 and Figure 2 in Gomez-Raya et al., 2013), sometimes the height of the two modes cannot be differentiated even when *p*_1_≠0.5 or *p*_2_≠0.5. Hence we introduce the interval [0.48, 0.52] for allele frequencies; this is denoted as the critical range. For allele frequencies outside this range, *p*_1_∨*p*_2_∉[0.48, 0.52], the modes of log*LF* differed clearly more than 10^−5^ in prior investigations using simulated data with *D*^*sire*^, *D*^*dam*^∈{0, 0.05, 0.15} and 100 half-sibs.

In view of the empirical data analysis, the log*LF* in Equation (1) was modified mildly by combining genotype frequencies. In a family with few progeny, not all genotype combinations may have been observed, i.e., some cells remained empty in a genotype-count table, and haplotype frequencies were then estimated to be close to zero. To level the influence of zero or almost-zero haplotype frequencies on the likelihood function, the none-observed genotypes and the least-observed genotype were combined into one cell. The corresponding set of index pairs was set up as $J=J_{0}\cup J_{1}$ where J_0_ = {*i, k*∈{*AA, AB, BB*}|*n*_*i, k*_ = 0} collects the indices from the empty cells, and $J_{1}=\underset{i,k\in \left\{AA,AB,BB\right\}}{\mathrm{argmin}}\left\{n_{i,k}|n_{i,k}>0\right\}$ refers to the genotype index with least observed frequency. This gave rise to a log LF with a combined cell:

$$\begin{array}{l} \log LF^{*}(\pi_{AA,AA},\pi_{AA,AB},\ldots ,\pi_{BB,BB}|n_{AA,AA},\ldots ,n_{BB,BB})\\ \;=\sum_{i,k\in \{AA,AB,BB\}\backslash J}n_{i,k}\log\pi_{i,k}+\left(\sum_{i,k\in J}n_{i,k}\right)\log\left(\sum_{i,k\in J}\pi_{i,k}\right) \end{array}$$

with parameters as above. Thus, compared to a strategy of leaving empty cells out, the probability of the least observed genotype is marginally increased.

The DP based on the value of logLF^*^ was applied to all locus pairs with allele frequencies outside the critical range.

### Decision process for critical allele frequencies

If the allele frequencies are within the critical range, *p*_1_, *p*_2_∈[0.48, 0.52], it is expected that the modes of log*LF* have almost equal height, and a decision about the final estimates is actually not possible. Only additional information about the physical position and estimates of θ in the neighborhood might allow selecting the most appropriate set of parameter estimates. If SNP *i* and *k* had estimates of allele frequency in the critical range, i.e., ${\hat{p}}_{i},{\hat{p}}_{k}\in \left[0.48,0.52\right]$, *i, k*∈{1, …, *m*_*c*_}, *i*<*k*; *m*_*c*_ denotes the number of SNPs on a particular chromosome*c*. Then the estimated recombination rates (${\hat{\theta }}_{i,j}$) between SNP *i* and all other SNPs *j*, *j* = 1, …, *m*_*c*_, *i*≠*j*, with non-critical allele frequency were used to determine a smoothing B-spline curve: The estimates ${\hat{\theta }}_{i,j}$ were regressed onto the corresponding physical position of the SNPs *j*, *j* = 1, …, *m*_*c*_, *i*≠*j*, using the *R* function “smooth.spline” considering five degrees of freedom (R Core Team, 2014). The physical position was scaled according to the whole chromosome length. Then, for SNP *k* with critical allele frequency, the value was selected out of the two proposals ${\hat{\theta }}_{I}$ and ${\hat{\theta }}_{II}$ yielding the minimum squared deviation to the fitted curve at the relative physical position of SNP *k*.

The stepwise procedure using EM twice followed by a DP based on either logLF^*^ or information from the neighborhood is denoted as EMDP. For comparison, EM estimates were also achieved based on fixed start values. In short, we applied three approaches to simulated and empirical data with the following start values:

– EMDP: θ = 0 and *D*^*dam*^ = 0, second set of start values was obtained from Equation (2),
– EMT0: θ = 0 and *D*^*dam*^ = 0,
– EMT05: θ = 0.5 and *D*^*dam*^ = 0.

### Detection of misplaced SNPs

The estimation of recombination rates based on empirical bovine data enables the identification of SNPs with wrong placement in the underlying genome assembly. SNPs revealing high estimates of θ with SNPs in close neighborhood or low estimates with distant SNPs are candidates for misplaced SNPs. In order to trace such SNPs, a statistic for unusually high estimates was calculated for each locus *i*∈{1, …, *m*_*c*_} as the average of the nearby *s* markers:

$${\hat{\theta }}_{i,c}^{H}=\{\begin{array}{l} \begin{array}{l} \frac{1}{s}\sum_{j=i+1}^{i+s}{\hat{\theta }}_{i,j};\text{if }i<m_{c}-s,\\ \frac{1}{s}\sum_{j=i-s}^{i-1}{\hat{\theta }}_{i,j};\text{if }i\geq m_{c}-s. \end{array} \end{array}$$

The number of SNPs considered in a local environment of the target SNP was *s* = 30; this choice will be discussed later. Similarly, for investigating the pattern of unusually low estimates, a statistic was computed for a window at the most distant chromosome end:

$${\hat{\theta }}_{i,c}^{L}=\{\begin{array}{l} \begin{array}{l} \frac{1}{s}\sum_{j=m_{c}-s+1}^{m_{c}}{\hat{\theta }}_{i,j}\;;\;i<\frac{m_{c}}{2},\\ \frac{1}{s}\sum_{j=1}^{s}{\hat{\theta }}_{i,j}\;;\text{ if }i\geq \frac{m_{c}}{2}. \end{array} \end{array}$$

The 99% quantile of ${\hat{\theta }}_{i,c}^{H}$ and 1% quantile of ${\hat{\theta }}_{i,c}^{L}$ over the entire genome (*i* = 1, …, *m*_*c*_; *c* = 1,.., 29) were taken as thresholds, and SNPs with ${\hat{\theta }}_{i,c}^{H}$ (${\hat{\theta }}_{i,c}^{L}$) above (below) the corresponding threshold were candidates for misplacement. The subset of selected SNPs was visually inspected afterwards, and conspicuously misplaced SNPs were also matched to the improved reference assembly UMD 3.1.1 (Merchant et al., 2014).

## Data

### Simulation

In order to validate the influence of start values on the parameter estimates, genotype data of a single half-sib family (*N* = 100 or *N* = 1, 000 progeny) were simulated at two loci. The sire was double-heterozygous in coupling phase, and the paternal haplotypes of progeny were determined considering a random recombination event during meiosis. The maternal haplotypes of progeny were drawn by chance from a dam population with a given LD between the two loci.

In a first study, three LD scenarios were simulated with 10,000 replicates each:

I) Small θ: *D*^*sire*^ = 0.245 (θ = 0.01); *D*^*dam*^ = 0.05,
II) Medium θ: *D*^*sire*^ = 0.15 (θ = 0.20); *D*^*dam*^ = 0.05,
III) Large θ: *D*^*sire*^ = 0.05 (θ = 0.40); *D*^*dam*^ = 0.15.

Different maternal allele frequencies were considered for each scenario: (a) *p*_1_ = *p*_2_ = 0.5; (b) *p*_1_ = *p*_2_ = 0.4; (c) *p*_1_ = 0.4, *p*_2_ = 0.6; and (d) *p*_1_ = *p*_2_ = 0.8. Scenarios II (a) and III (a) are complementary settings. A balanced design, i.e., *D*^*sire*^ = *D*^*dam*^, leads to a unimodal log*LF* and is not considered here. As only a single SNP pair was simulated, the DP was reduced to comparing the logLF^*^ values only.

In a second simulation study, θ was varied in a range from zero to 0.5 with step size 0.005. For each value, genotypes of *N* = 100 or *N* = 1, 000 half-sibs and 1,000 replicates were simulated. Separate runs of the simulation were executed with fixed allele frequency at the first locus, *p*_1_ = 0.4 or *p*_1_ = 0.5, but varying frequency at the second locus: *p*_2_ was randomly drawn from a uniform distribution on the interval [0.3, 0.7]. In each run *D*^*dam*^ was fixed at 0.05 or 0.10. Hence, 8 scenarios were studied in total.

Data simulation and all analyses were carried out with programs written in *R* (R Core Team, 2014).

### Empirical dataset

The stepwise procedure was applied to an empirical dataset consisting of multiple families of Holstein-Friesian cows and 39,780 SNP genotypes on the autosomes; data and quality control were described in Melzer et al. (2013). SNPs were ordered according to the Btau 4.2 assembly (Bovine HapMap Consortium, 2009). Sires with at least 30 progeny were selected; hence five paternal half-sib families comprising 265 cows remained. The phases of the non-genotyped sires were estimated using the *R* package *hsphase* version 2.0.0 (Ferdosi et al., 2014). Then, 8,512 loci were disregarded because all sires were homozygous. The number of intrachromosomal SNP pairs expected from the number of SNPs was reduced by the quantity of SNP pairs at which only sires with homozygous-heterozygous or heterozygous-homozygous haplotypes were observed (roughly one third of SNP pairs); 12,759,713 SNP pairs within chromosomes remained for the analysis. The estimate of *D*^*dam*^ and its standardized measure *r*^2^ were determined from the estimated haplotype frequencies.

The pattern search to detect misplaced SNPs was validated by rearranging SNPs within and between chromosomes. Firstly, a single SNP was moved on BTA4 from index position 3 to position 1,000 as well as a SNP cluster on BTA4 from index positions 10–15 to 1,602–1,607. Secondly, single SNPs were moved from BTA20 (index positions 1 and 1,000) to BTA4 (index positions 100 and 1,000) as well as to a SNP cluster from BTA1 (index positions 411–420) to BTA4 (index positions 411–420).

The empirical data are provided in Files S2, S3. Marker names and positions according to the genome assemblies Btau 4.2 and UMD 3.1.1 are listed in File S4.

## Results

### Simulated data

Using either one set of fixed start values (EMT0 or EMT05) or two proposals (EMDP) influenced the estimates of disequilibrium in sires and dams. The first simulation study showed that EMT0 and EMDP performed equally well for the small-θ setting in terms of bias and MSE of $\hat{\theta }$ and ${\hat{D}}^{dam}$. For the medium- and large-θ setting, EMT0 and EMT05, respectively, had the least bias and MSE (Tables 1, 2) when *N* = 100 half-sibs were considered. In such cases, EMDP was the second-best choice. If *N* = 1, 000, bias and MSE reduced for all approaches and EMDP performed best overall in terms of MSE (Tables S1, S2). Furthermore, it was obvious from the two-locus investigation that a decision solely based on logLF^*^ is not sufficient if *p*_1_ = *p*_2_ = 0.5. Using EMDP in such a case and *N* = 1, 000, both modes were selected at almost equal proportion in the medium- and large-θ scenario. However, if *N* = 100, in 58.0% and 57.9% of the repetitions in the medium-θ and small-θ scenarios, respectively, the decision was in favor of the mode derived from the default start values. In the small-θ scenario, the mode derived from the default start values yielded most often the higher value of logLF^*^: in 95.5% of the repetitions if *N* = 100 and 81.1% if *N* = 1, 000.

**Table 1:** Bias of estimated paternal recombination rate and maternal linkage disequilibrium for simulated scenarios.

		**bias** $\hat{\theta }$				**bias** ${\hat{D}}^{dam}$			
		***p*_1_ = *p*_2_ = 0.5**	***p*_1_ = *p*_2_ = 0.4**	***p*_1_ = 0.4, *p*_2_ = 0.6**	***p*_1_ = *p*_2_ = 0.8**	***p*_1_ = *p*_2_ = 0.5**	***p*_1_ = *p*_2_ = 0.4**	***p*_1_ = 0.4, *p*_2_ = 0.6**	***p*_1_ = *p*_2_ = 0.8**
θ = 0.01, *D*^*dam*^ = 0.05	EMT05	0.153	0.079	0.023	0.013	0.073	0.036	0.009	0.002
	EMT0	**0.002**	**0.003**	**0.001**	**0.002**	−**0.001**	−**0.001**	−**0.001**	−**0.001**
	EMDP	0.016	0.009	0.002	**0.002**	0.006	0.002	−**0.001**	−**0.001**
θ = 0.20, *D*^*dam*^ = 0.05	EMT05	0.097	0.059	0.043	**0.000**	0.051	0.031	0.022	0.003
	EMT0	**0.012**	**0.011**	**0.009**	−0.014	**0.008**	**0.007**	**0.006**	−**0.002**
	EMDP	0.069	0.042	0.018	−0.014	0.036	0.023	0.010	−**0.002**
θ = 0.40, *D*^*dam*^ = 0.15	EMT05	−**0.106**	−**0.080**	−**0.053**	−**0.013**	−**0.051**	−**0.039**	−**0.025**	−**0.010**
	EMT0	−0.187	−0.163	−0.123	−0.252	−0.092	−0.080	−0.060	−0.095
	EMDP	−0.130	−0.109	−0.105	−0.248	−0.064	−0.053	−0.051	−0.093

*Start values have been fixed (EMT0 and EMT05) or adapted (EMDP), 100 half-sibs were simulated with 10,000 replicates. The best outcome for each parameter setting is highlighted in bold*.

**Table 2:** MSE of estimated paternal recombination rate and maternal linkage disequilibrium for simulated scenarios.

		**MSE** $\hat{\theta }$				**MSE** ${\hat{D}}^{dam}$			
		***p*_1_ = *p*_2_ = 0.5**	***p*_1_ = *p*_2_ = 0.4**	***p*_1_ = 0.4, *p*_2_ = 0.6**	***p*_1_ = *p*_2_ = 0.8**	***p*_1_ = *p*_2_ = 0.5**	***p*_1_ = *p*_2_ = 0.4**	***p*_1_ = 0.4, *p*_2_ = 0.6**	***p*_1_ = *p*_2_ = 0.8**
θ = 0.01, *D*^*dam*^ = 0.05	EMT05	0.061	0.030	0.009	0.002	0.014	0.008	0.002	**0.001**
	EMT0	**0.001**	**0.002**	**0.001**	**0.001**	**0.001**	**0.001**	**0.001**	**0.001**
	EMDP	0.007	0.004	**0.001**	**0.001**	0.002	**0.001**	**0.001**	**0.001**
θ = 0.20, *D*^*dam*^ = 0.05	EMT05	0.030	0.022	0.018	**0.008**	0.008	0.006	0.004	**0.002**
	EMT0	**0.014**	**0.013**	**0.012**	0.010	**0.003**	**0.003**	**0.002**	**0.002**
	EMDP	0.026	0.020	0.014	0.010	0.006	0.005	0.003	**0.002**
θ = 0.40, *D*^*dam*^ = 0.15	EMT05	**0.032**	**0.026**	**0.020**	**0.006**	**0.008**	**0.006**	**0.004**	**0.002**
	EMT0	0.048	0.045	0.035	0.099	0.011	0.010	0.007	0.013
	EMDP	0.038	0.034	0.032	0.097	0.009	0.007	0.007	0.012

*Start values have been fixed (EMT0 and EMT05) or adapted (EMDP), 100 half-sibs were simulated with 10,000 replicates. The best outcome for each parameter setting is highlighted in bold*.

**Figure 2:**
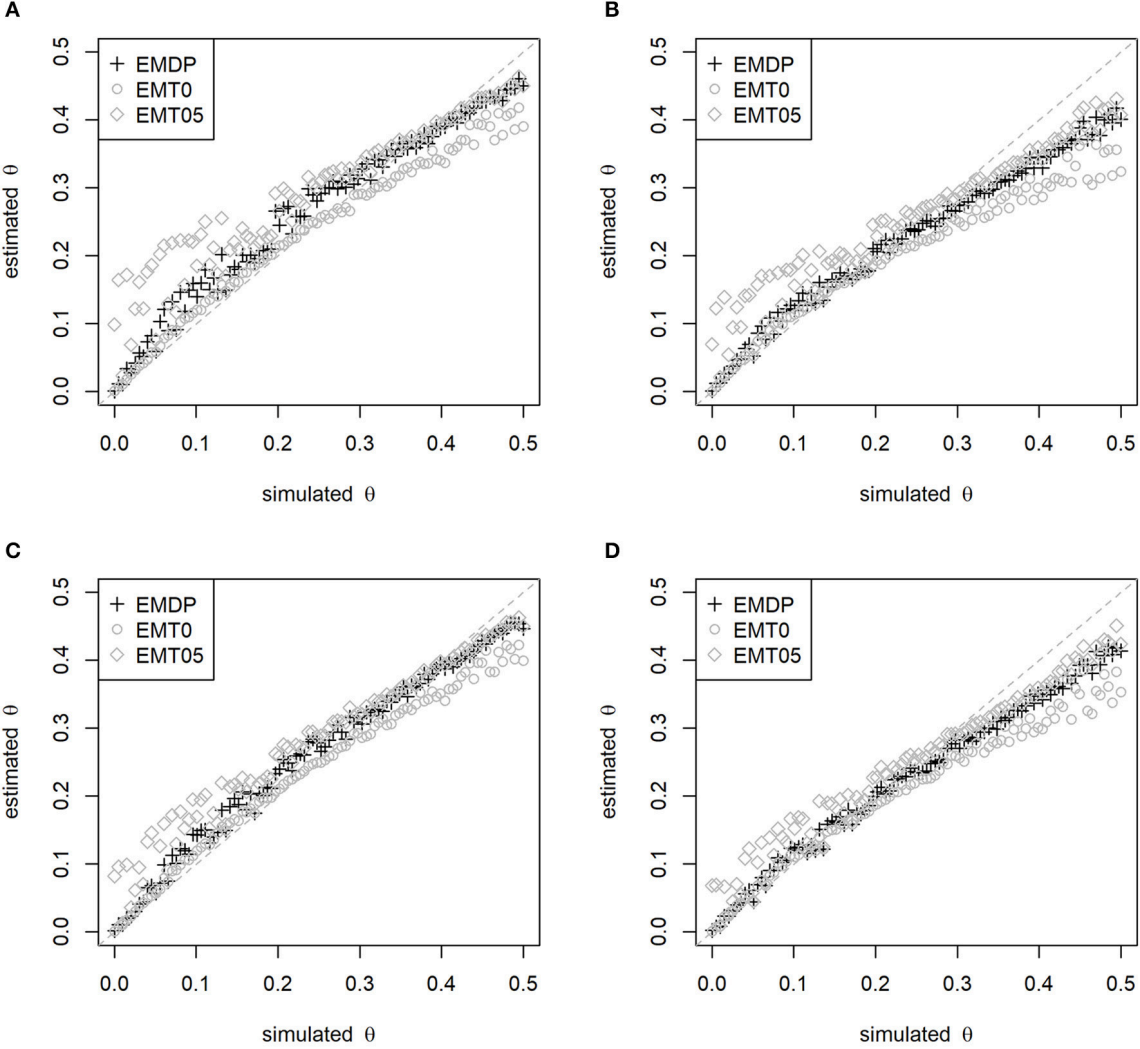
Estimated vs. simulated recombination rate depending on start-value strategy. Start values have been fixed (EMT0 and EMT05) or adapted (EMDP). Simulated maternal allele frequency and LD: **(A)** *p*_1_ = 0.5, *D*^*dam*^ = 0.05, **(B)** *p*_1_ = 0.5, *D*^*dam*^ = 0.10, **(C)** *p*_1_ = 0.4, *D*^*dam*^ = 0.05, and **(D)** *p*_1_ = 0.4, *D*^*dam*^ = 0.10. In total, 100 half-sibs were simulated with 1,000 replicates.

The second simulation study confirmed the ranking of methods as described above. Moreover, an impact of the simulated maternal LD on the estimates of θ was observed. Using *N* = 100, EMDP had the least bias of $\hat{\theta }$ in the upper range (θ>0.30) if *D*^*dam*^ = 0.05 (Figures 2A,C) and the least bias in the lower range (θ < 0.05) if *D*^*dam*^ = 0.10 (Figures 2B,D). The bias of $\hat{\theta }$ was reduced for all approaches if *p*_1_ = 0.4 instead of *p*_1_ = 0.5 was simulated as well as when *N* = 1, 000 half-sibs were considered (Figure S1). In summary, EMDP has led to robust estimates of θ and *D*^*dam*^ being least susceptible to changes in the parameter settings compared with EMT0 and EMT05.

### Parameter estimation in cattle

**Figure 3:**
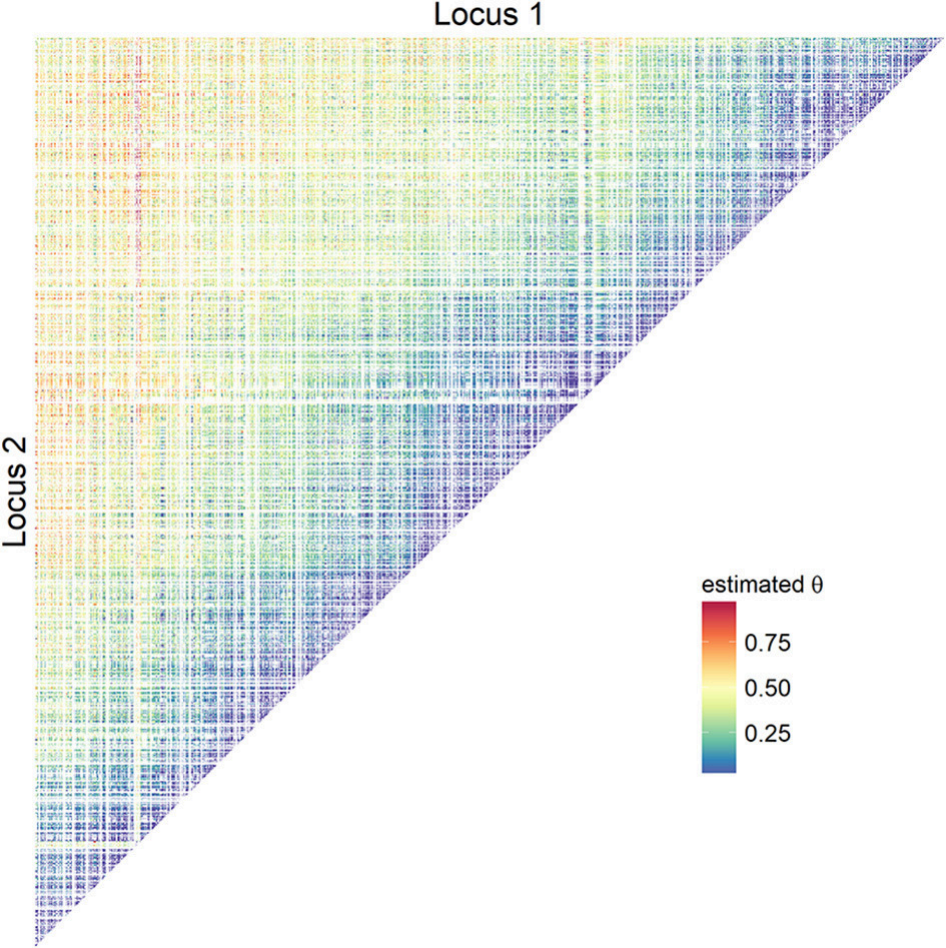
Estimates of recombination rates using empirical bovine data. Pairwise recombination rates on BTA5 were obtained using adapted start values (EMDP).

For the empirical data analysis, the results of estimating θ and *D*^*dam*^ based on EMDP are described. Loci at which all sires were homozygous did not contribute to parameter estimations, and they are shown blank in all figures. Usually, the chance of recombination was lower in the immediate neighborhood than for more distant SNPs: lower estimates of θ were found between the next 50 neighboring SNPs (about 3.2 Mb range, Figure 3 and Figure S2), and $\hat{\theta }$ increased with increasing distance between loci. Typical triangles with lower estimates showing regions of high paternal LD were also found for more distant SNPs.

The estimated *D*^*dam*^ between distant SNPs was generally close to zero but specific regions existed where ${\hat{D}}^{dam}\pm 0.2$ (see Figure S3; the corresponding *r*^2^ is shown in Figure S4). High LD was estimated near the center on BTA10 (23,319.73 kb range, mean *r*^2^ = 0.170), BTA13 (16,326.63 kb range, mean *r*^2^ = 0.140) and BTA20 (13,870.19 kb range, mean *r*^2^ = 0.179). Smaller regions of high LD were also observed outside the center, e.g., on BTA14 (3,811.90 kb range, mean *r*^2^ = 0.178), BTA23 (474.85 kb range, mean *r*^2^ = 0.699), and BTA29 (3,474.42 kb range, mean *r*^2^ = 0.176). Such regions may indicate the presence of cold spots.

**Figure 4:**
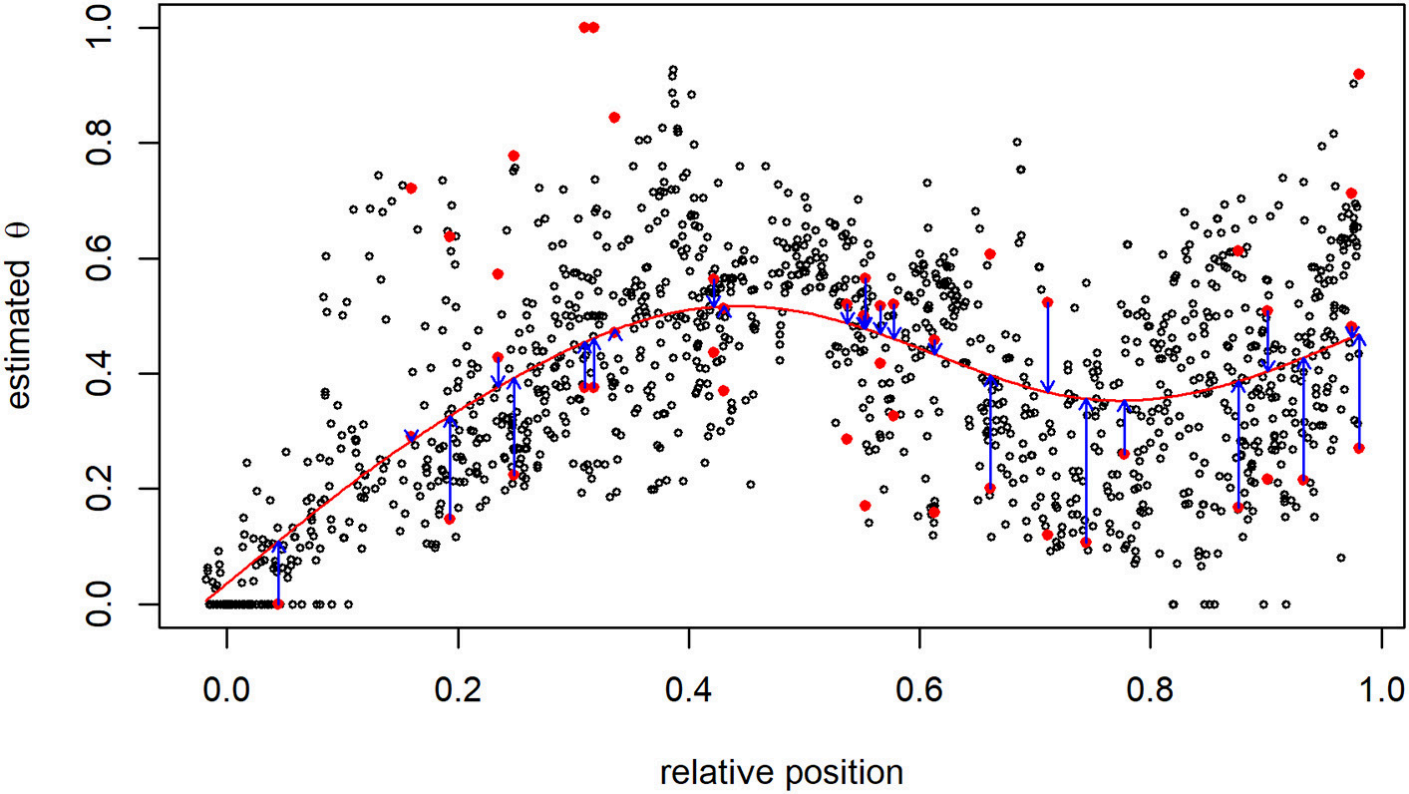
Recombination rate between the 61st and all other SNPs on BTA1. The pair of proposals at SNPs with critical allele frequency are shown as red points, and smoothing via B-splines (red line) was used to identify the most suitable estimate having the smallest distance to the B-spline curve (blue arrows).

Among all SNP pairs, 37,040 (0.3%) estimates of θ followed the rules of the DP for critical maternal allele frequencies: the most appropriate $\hat{\theta }$ was determined using the cubic B-spline smoothing approach. For example, considering SNP 61 on BTA1, 25 mates with critical allele frequency existed: 61 × 188, 61 × 424, 61 × 510, 61 × 604, 61 × 644, …, 61 × 2,539, and 61 × 2,560. Estimates based on mates with SNP 61 having a non-critical allele frequency were employed to generate the B-spline curve (Figure 4). Five degrees of freedom were equivalent to 152 proper knots in this case. Then, for instance, the two proposals for SNP pair 61 × 604 were ${\hat{\theta }}_{I}=0.428$ and ${\hat{\theta }}_{II}=0.572$, and the first was closer to the fitted curve.

In 99.7% of pairwise investigations, a comparison of log*LF* at the two proposals yielded the final estimate of genetic parameters. The decision was in favor of the first proposal, which was derived from default start values θ = 0 and *D*^*dam*^ = 0, in 72.13% regarding all chromosomes. On the other hand, in 27.87% of pairwise investigations, the proposal obtained from the complementary relationship (Equation 2) yielded the larger log*LF*; this makes EMDP superior to EMT0. Either way, the smaller $\hat{\theta }$ out of the two proposals was related to the larger likelihood in about 89% of the comparisons.

Taking the SNPs with statistics ${\hat{\theta }}_{i,c}^{H}$ above and ${\hat{\theta }}_{i,c}^{L}$ below the corresponding thresholds and looking at the pattern of $\hat{\theta }$ in the rows or columns of the matrix representation, we concluded that individual SNPs or even clusters of SNPs were wrongly placed on some chromosomes. For instance, a SNP cluster was identified on BTA5 ranging from 10.11 Mb to 10.86 Mb (13 SNPs; rs110564524 to rs110035083, see Table 3) where $\hat{\theta }$ was exceptionally high not only with very distant SNPs but also with adjacent SNPs. (If non-informative SNPs occurred within such a cluster, they were declared candidates as well.) We compared the chromosome assignment, the SNP order and the base pair position for all SNPs with putatively wrong placement to the revised *Bos taurus* assembly UMD 3.1.1. The aforementioned SNP cluster on BTA5 had the same chromosome assignment but the base pair position now ranged from 103.47 to 104.23 Mb based on UMD 3.1.1. The order of SNPs within this cluster remained. A second SNP cluster was found on BTA3 (rs109129646, rs41661711, rs41665543) which was now assigned to BTA2 based on UMD 3.1.1. Furthermore, the order of SNPs within this cluster was reversed. The rsID of a single SNP on BTA2 (BTA-45006; position: 10,760,381 bp) was unavailable. The DNA sequence of this SNP derived from Btau 4.2 was mapped to the sequence based on UMD 3.1.1 using Bowtie 2 (Langmead and Salzberg, 2012); the sequence fitted to BTA4 with position 33,987,225 bp. This result is similar to that of Khatkar et al. (2010), who determined this SNP on BTA4 at 33,987,224 bp based on UMD 3.0. Furthermore, the manually replaced SNPs and SNP clusters were detected as misplacements (see Figure S5). The complete list of 40 putatively misplaced SNPs, mostly confirmed with UMD 3.1.1, is given in Table 3.

**Table 3:** List of misplaced SNPs in the genome assembly Btau 4.2 in comparison to the improved assembly UMD 3.1.1.

		**Btau 4.2**			**UMD 3.1.1**		
**rsID**	**Synonym**	**Chr**	**Index Chr**	**Position bp**	**Chr**	**Index Chr**	**Position bp**
41646101	BTA-49243	1	370	24,597,406	1	368	24,338,769
43237745	ARS-BFGL-NGS-101533	1	781	51,186,654	1	788	51,033,230
NA	BTA-45006	2	179	10,760,381	4	1,915	33,987,225
109129646	ARS-BFGL-NGS-31620	3	1,048	77,548,374	2	1,739	118,217,699
41661711	BTA-94835	3	1,049	77,635,918	2	1,738	118,130,312
41665543	BTA-98418	3	1,050	77,712,209	2	1,737	118,054,344
109560518	ARS-BFGL-NGS-16314	3	1,970	127,186,019	22	1,024	61,258,859
43504210	ARS-BFGL-NGS-105794	3	1,971	127,245,912	22	1,023	61,199,665
110389139	ARS-BFGL-NGS-92218	3	1,972	127,272,424	22	1,022	61,172,869
109112664	ARS-BFGL-NGS-67185	3	1,973	127,296,240	22	1,021	61,149,265
29018845	rs29018845	3	1,974	127,346,352	22	1,020	61,099,118
43505889	BTB-00297874	3	1,975	127,377,195	22	1,019	61,069,286
110104891	ARS-BFGL-NGS-4905	3	1,976	127,407,117	22	1,018	61,040,701
110739182	ARS-BFGL-NGS-25064	3	1,977	127,432,016	22	1,017	61,015,806
41586006	BTA-55057	3	1,978	127,462,142	22	1,016	60,985,682
109051741	ARS-BFGL-NGS-30266	3	1,979	127,516,161	22	1,015	60,930,725
110904383	ARS-BFGL-NGS-33950	3	1,980	127,552,306	22	1,014	60,897,444
29011651	BTA-03895	3	1,981	127,572,719	22	1,013	60,877,108
43506988	ARS-BFGL-NGS-90519	3	1,982	127,599,954	22	1,012	60,850,524
110144486	ARS-BFGL-NGS-70541	3	1,983	127,660,653	22	1,011	60,785,491
111007440	ARS-BFGL-NGS-58676	3	1,984	127,710,668	22	1,010	60,736,089
109871231	ARS-BFGL-NGS-114675	3	1,985	127,759,149	22	1,009	60,691,260
110218851	ARS-BFGL-NGS-42322	3	1,986	127,790,957	22	1,008	60,659,773
110564524	BTA-74753	5	178	10,108,998	5	1,330	103,472,289
110171642	ARS-BFGL-NGS-1422	5	179	10,175,658	5	1,331	103,539,042
110040410	ARS-BFGL-NGS-101402	5	180	10,211,997	5	1,332	103,583,249
109446437	ARS-BFGL-NGS-30033	5	181	10,288,674	5	1,333	103,659,884
41592968	BTA-74776	5	182	10,456,513	5	1,334	103,821,233
109366282	ARS-BFGL-NGS-678	5	183	10,495,942	5	1,335	103,860,658
109046936	ARS-BFGL-NGS-18320	5	184	10,545,228	5	1,336	103,911,258
109283161	BTA-143449	5	185	10,590,963	5	1,337	103,957,794
109955767	ARS-BFGL-NGS-118997	5	186	10,669,337	5	1,338	104,038,847
110253084	ARS-BFGL-NGS-52457	5	187	10,744,828	5	1,339	104,116,518
41654485	BTA-74821	5	188	10,769,105	5	1,340	104,140,809
110872738	ARS-BFGL-NGS-26592	5	189	10,815,342	5	1,341	104,188,186
110035083	ARS-USMARC-201	5	190	10,857,544	5	1,342	104,230,386
4.5010507.4 43521798	BTB-00316147	7	989	61,935,287	17	229	12,137,554
43696553	ARS-BFGL-NGS-69516	12	760	53,996,667	12	755	53,756,393
4.5010507.4 42103498	ARS-BFGL-NGS-77278	19	544	34,916,761	26	753	46,541,332
41255607	BTB-00780480	20	606	38,986,288	20	602	36,757,600

*The misplaced SNPs confirmed by UMD 3.1.1 (based on discrepancy in SNP position or chromosome) are marked gray*.

## Discussion

The recombination rate and maternal LD were estimated using an EM algorithm. Due to the bimodality of the underlying LF, two sets of start values were proposed to guide the iterative process into the right directions. With EMDP, the most likely estimate was selected out of the two proposals. A B-spline smoothing method was suggested for SNP pairs with critical maternal allele frequencies. The approach was validated using simulated genotypes of half-sibs, and it was applied to empirical dairy cattle data to identify putatively misplaced SNPs. The position of SNPs with unusually large estimates of θ was compared to the improved bovine genome assembly UMD 3.1.1.

### Recombination rate

The “direct method” of linkage analysis requires the haplotype phases of parents and offspring to be known; they might be inferred from genotypes ordered according to a given genome assembly (e.g., using LINKPHASE3 for half-sib families, Druet and Georges, 2015). As an option, approaches for long-range phasing of haplotypes (e.g., Daetwyler et al., 2011; Hickey et al., 2011; Ferdosi et al., 2014) enable determining the location of recombination events. Such an algorithm deduces blocks of haplotypes passed from parent to offspring. From this block structure, the number of break points can be counted and the recombination rate can be estimated. The advantage of the proposed method is that only the sire's haplotypes and progeny genotypes are required for estimating the recombination rates and the error caused by phasing offspring genotypes are circumvented. A comparison of methods is worth further investigation.

Assuming that the maternal allele frequencies are known, the recombination rate and maternal LD can also be inferred from the reduced LF where θ and *D*^*dam*^ are the unknown parameters. In this reduced LF case, the maximum of log*LF* can quickly be obtained numerically. For instance, the function “optim” in *R* can consider the boundaries of the parameter space of θ and *D*^*dam*^ using the option “L-BFGS-B”. Though the proposed sets of start values can also be subjected to the numerical optimization procedure, we have observed that some estimates have been stuck in one region of the parameter space and lead to the same maximum (results not shown). This outcome might be explained by the algorithm-internal step-width regularization. To circumvent this, the two local maxima can be sought via grid search. This procedure is feasible in the reduced LF case but not recommended for a search in the whole parameter space considering not only θ and *D*^*dam*^ but also the allele frequencies as unknown parameters (or, equivalently, treating θ and the maternal haplotype frequencies as unknowns).

For various species it is known that the recombination rate varies between males and females (e.g., mouse, Liu et al., 2014; salmon, Moen et al., 2004). This was also observed for dairy cattle (e.g., Ma et al., 2015). Moreover, the recombination rate may also be different among individuals. Kadri et al. (2016) identified causal variants with effect on an individual's number of recombination events in cattle. In general, the EM approach can be applied to each sire family separately, thus allowing the estimation of sire-specific recombination rates. Once the number of genome-wide or chromosome-wide recombination events would have been inferred from $\hat{\theta }$'s of each sire, it could be further studied which loci are relevant to the variation of recombination events. This issue claims further investigation based on a larger number of sires.

The empirical data analysis required smoothing of estimates of θ to select the final estimate in the ambiguous case where ${\hat{p}}_{1},{\hat{p}}_{2}\in \left[0.48,0.52\right]$. The proposal bearing the least squared error with its predicted outcome on the curve was chosen. For convenience, a B-spline smoothing approach was employed with a fixed degree of smoothness. In contrast to fixing the degree of smoothness, as it was also done in Ma et al. (2015), the number and position of knots can be estimated using a Bayesian approach (Zhang et al., 2003). Preliminary investigations on the decision of knots revealed only minor differences in the shape of the smoothing curve (results not shown). Thus, it is expected that the DP is hardly influenced by this outcome. Other methods for selecting the more appropriate estimate are conceivable. They might be based on a measure of similarity. For instance, the sampling variance of estimates in a certain window around the investigated locus-pair mate can be considered, including either ${\hat{\theta }}_{I}$ or ${\hat{\theta }}_{II}$. The higher measure of similarity, i.e., the lower sampling variance, would point to the more suitable estimate.

Estimates of recombination rate can reveal regions of hot and cold spots (e.g., Sandor et al., 2012; Weng et al., 2014). Our results based on LD estimates are in close agreement with those studies: cold spots were mainly found at the chromosome center or proximal chromosome end. Studies of human populations have shown the co-occurrence of runs of homozygosity (ROH) in regions with extended LD and low recombination rates (Gibson et al., 2006; Curtis et al., 2008). We did not investigate ROH distribution in our material but our results support the existence of LD blocks in regions with low recombination rate.

### Order of SNPs

The physical order of SNPs has been updated regularly, and misplacements have been corrected successively (e.g., Milanesi et al., 2015). In this study, misplacement of SNPs was inferred from unusually large estimates of θ: SNPs having low $\hat{\theta }$ to distant SNPs or large $\hat{\theta }$ to close SNPs were candidates for misplacement. The inspection of $\hat{\theta }$'s followed heuristic rules, and it enabled the identification of the most serious candidates. The pattern search was based on a window size of 30 SNPs. Choosing, for instance, *s* = 300 yielded 38 candidates of misplacement (rs43237745 on BTA1 and rs43696553 on BTA12 were not detected). Such a large window could be counteractive: it would definitely detect candidates being putatively placed on another chromosome but might fail to detect candidates which have a different position on the same chromosome.

The genome assembly UMD 3.1.1 was used to confirm candidates for misplacement in the older assembly Btau 4.2, hence providing evidence that the suggested pattern search is convenient. Additionally, the approach was validated based on manually misplaced SNPs. The comparison of genome assemblies leaves some candidates which shall be matched to the most recent assembly (http://bovinegenome.org/). Those candidates that cannot be matched may be further explored based on information about the raw data used for the current assembly. As, for instance, not only coverage but also read lengths of contigs and mate-pair distances have an impact on the quality of the genome build (Schatz et al., 2010), the value of such parameters has to be verified for suspicious SNPs. Eventually, an eligible strategy of replacement might consider the same criteria as those used for the identification of misplacements: in an iterative approach, a window measure seeks the position showing the least θ estimates to neighboring SNPs and the maximum estimates to distant SNPs, which is similar to genetic mapping algorithms (e.g., Falk, 1989; see Cheema and Dicks, 2009 for a review of methods).

A more formal approach of identifying misplacements is testing the mean of $\hat{\theta }$'s in a local environment of the investigated SNP. This can be achieved by a statistical test, such as a *t*-test. Under the null hypothesis a recombination rate close to zero is expected. Such an approach bears a severe difficulty. As a recombination event likely follows a binomial distribution, the mean and variance approach zero under the null, and the null distribution of the test statistic cannot be determined. Alternatively, as the Fisher information *F* based on the log*LF* (Equation 1) is available (see File S1), an asymptotic test based on the asymptotic normality of *D*^*sire*^ is conceivable. Because two-locus genotype frequencies—also the expected ones—may be zero in small samples, division by zero appears in terms of *F*, see Equations (S10)–(S16) in the File S1. Likewise, the term log(0) appears in a likelihood ratio test at particular SNP pairs. Hence locus-pair-specific tests are not applicable to all locus combinations in the local environment.

Not only does the investigation of $\hat{\theta }$ enable the identification of misplacements, but also that of the pattern of LD. Verifying the decay of maternal LD, Bohmanova et al. (2010) detected 223 misplaced SNPs in the Btau 4.0 assembly. Utsunomiya et al. (2016) even identified 2,906 SNPs with unexpected LD decay in the UMD 3.1 assembly; our set of candidates was not among this set. Furthermore, the presence of LD between unlinked markers (on different chromosomes) is an indication of misplacement. However, unusually large non-syntenic LD was not observed in the study of Bohmanova et al. (2010) though the underlying assembly Btau 4.0 contained wrong chromosome assignments in comparison to the improved assembly UMD 3.1.1.

### Half-Sib family

The proposed method is applicable not only to paternal but also to maternal half-sib families as they appear, for instance, in insects (Milne and Friars, 1984) or fish (Gjerde et al., 2004).

Half-sib families of dairy cattle typically contain many offspring. For instance, on average 828 offspring were reported for German Holstein bulls (median 172.5, minimum 12.0 and maximum 25,590; Vereinigte Informationssysteme Tierhaltung, 2017). A minimum family size of 30 was accepted in our study, allowing for phasing the sire haplotypes. Only the offspring of double-heterozygous sires were considered. To reduce the estimation error of the EM approach, the information from families of homozygous-heterozygous and double-homozygous sires may also be used in the estimation of θ and *D*^*dam*^. In such families, there is no means to estimate θ, and haplotype frequencies can be estimated using simplified equations (Gomez-Raya, 2012). Haplotype frequencies are then treated to be known while the recombination rate is estimated for double-heterozygous sires.

### Chromatid interference

In some cases, the smoothing curve adopts $\hat{\theta }$-values larger than 0.5. This may be a non-significant outcome and may occur by chance, but otherwise it can be explained by two reasons: (I) the uncertainty about the sire's estimated haplotype phase or (II) the presence of chromatid interference of recombination events. Additionally, a non-monotonic behavior of the smoothing curve was observed (see Figure 4) which is another and more severe indication of chromatid interference (Figure 6 in Stam, 1979; Zhao et al., 1995). The specification of the second set of start values does allow for chromatid interference. Excluding chromatid interferences requires some minor adjustment of the proposed method: in the start-value derivation step, θ has to be restricted to lay in [0, 0.5] and hence *D*^*sire*^∈[0, 0.25] in the coupling phase. The extent of genetic and chromatid interference shall be studied in more depth in future. It may help fitting a mapping function to the estimated θ's and improving the corresponding genetic map.

## Conclusions

The theoretical consideration of the likelihood function shows that the maternal allele frequencies have an influence on the position and height of its maxima. We employed an expectation maximization algorithm with two sets of start values to estimate recombination rate and maternal linkage disequilibrium near the two modes. Knowing the two modes gave a competitive edge on the parameter estimates, particularly if the maternal allele frequencies at the investigated SNP pair were about 0.5. In this case, a decision rule was proposed for selecting the most suitable proposal based on estimates in the neighborhood and the corresponding physical position. This study also showed that half-sib families can be used for the identification of misplaced SNPs in the genome assembly due to the information provided from linkage analyses and linkage disequilibrium coming from maternal contribution. A correct physical order is helpful for further investigating the genetic distance between SNPs and refining the bovine genetic map.

## Ethics statement

Animal Care and Use Committee approval was not obtained for this study because the blood samples were collected during a veterinary routine examination in 2009.

## Author contributions

AH implemented the theory, developed the simulation design, analyzed the data and drafted the manuscript. FT raised the initial question, contributed to the theoretical investigations and to the discussion of the results. LG-R contributed to the theoretical investigations and suggested improvements to the manuscript. MD contributed to the investigations of the likelihood function. DW supervised the project and contributed to the theoretical derivations, data analysis, discussion of results, and writing of the manuscript. All of the authors have read and approved the final manuscript.

### Conflict of interest statement

The authors declare that the research was conducted in the absence of any commercial or financial relationships that could be construed as a potential conflict of interest.

## Supplementary material

Figure S1 Estimated vs. simulated recombination rate depending on start-value strategy. Start values were fixed (EMT0 and EMT05) or adapted (EMDP). Simulated maternal allele frequency and LD: **(A)** *p*_1_ = 0.5, *D*^*dam*^ = 0.05, **(B)** *p*_1_ = 0.5, *D*^*dam*^ = 0.10, **(C)** *p*_1_ = 0.4, *D*^*dam*^ = 0.05, and **(D)** *p*_1_ = 0.4, *D*^*dam*^ = 0.10. In total, 1,000 half-sibs were simulated with 1,000 replicates.

Figure S2 Estimates of paternal recombination rate for all autosomes using empirical bovine data. Pairwise recombination rates were obtained using the stepwise procedure EMDP.

Figure S3 Estimates of maternal linkage disequilibrium for all autosomes using empirical bovine data. Pairwise linkage disequilibria were obtained using the stepwise procedure EMDP.

Figure S4 Estimates of *r*^2^ for all autosomes using empirical bovine data. Pairwise *r*^2^'s were obtained using the stepwise procedure EMDP.

Figure S5 Validation of recombination-rate estimates using empirical bovine data on BTA4. Pairwise recombination rates were obtained using the stepwise procedure EMDP. BTA4 contained SNPs which were misplaced **(A)** within and **(B)** between chromosomes.

Table S1 Bias of estimated paternal recombination rate and maternal linkage disequilibrium for simulated scenarios. Start values were fixed (EMT0 and EMT05) or adapted (EMDP), 1,000 half-sibs were simulated with 10,000 replicates.

Table S2 MSE of estimated paternal recombination rate and maternal linkage disequilibrium for simulated scenarios. Start values were fixed (EMT0 and EMT05) or adapted (EMDP), 1,000 half-sibs were simulated with 10,000 replicates.

File S1 Investigations on the likelihood function and derivation of the Hessian matrix.

File S2 Genotype data consisting of 39,780 SNPs (columns), and 265 progeny (rows).

File S3 Assignment of genotypes to half-sib families.

File S4 Physical position of SNPs based on assembly Btau 4.2 and UMD 3.1.1.
