# Supplementary figures and images for: Estimation of Recombination Rate and Maternal Linkage Disequilibrium in Half-Sibs
Source: Front Genet. 2018 Jun 5;9:186. doi: 10.3389/fgene.2018.00186 (PMC5996054; doi:10.3389/fgene.2018.00186)

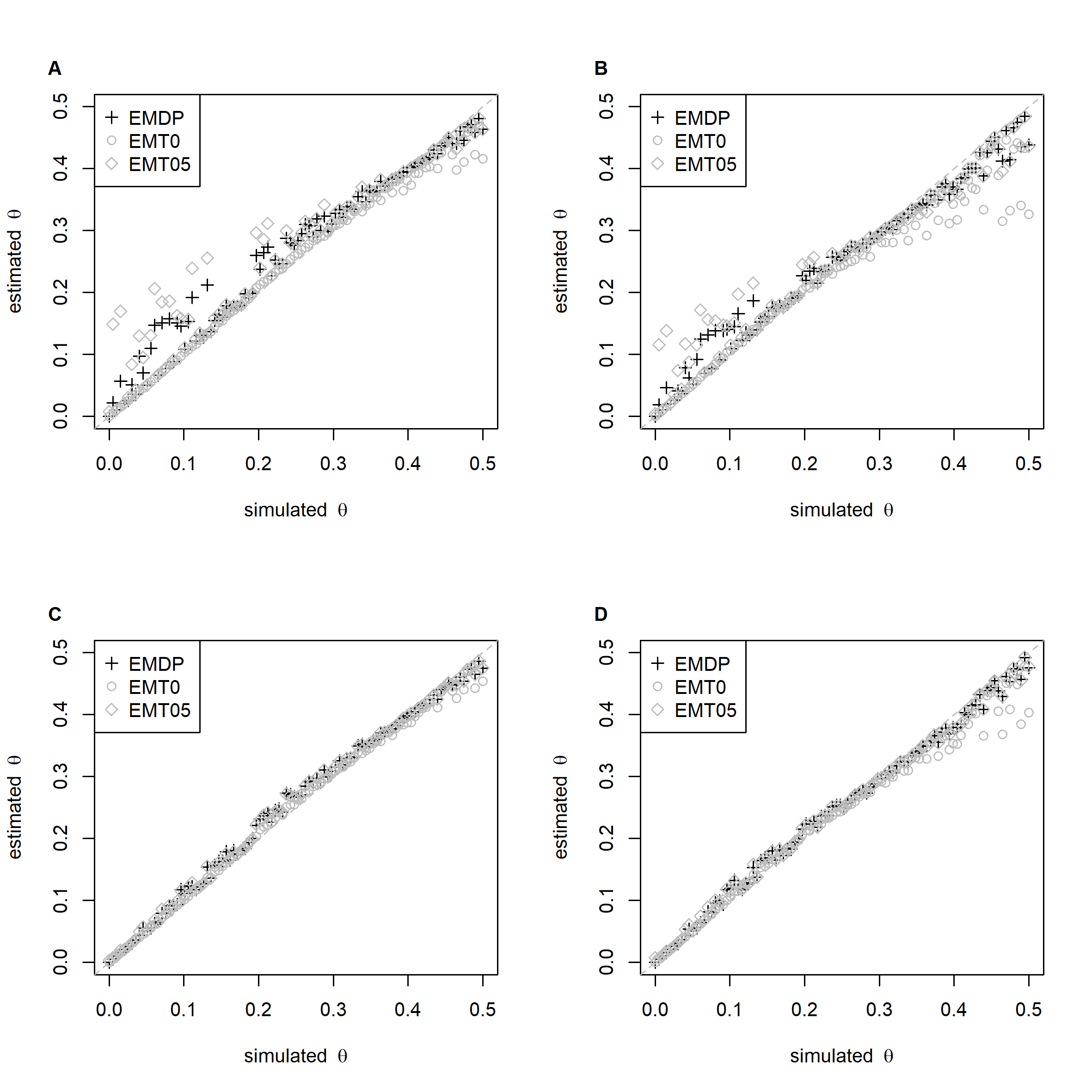

Supplement: Figure S1 — Estimated vs. simulated recombination rate depending on start-value strategy. Start values were fixed (EMT0 and EMT05) or adapted (EMDP). Simulated maternal allele frequency and LD: (A) p1 = 0.5, Ddam = 0.05, (B) p1 = 0.5, Ddam = 0.10, (C) p1 = 0.4, Ddam = 0.05, and (D) p1 = 0.4, Ddam = 0.10. In total, 1,000 half-sibs were simulated with 1,000 replicates. [file Image_1.tiff]

A

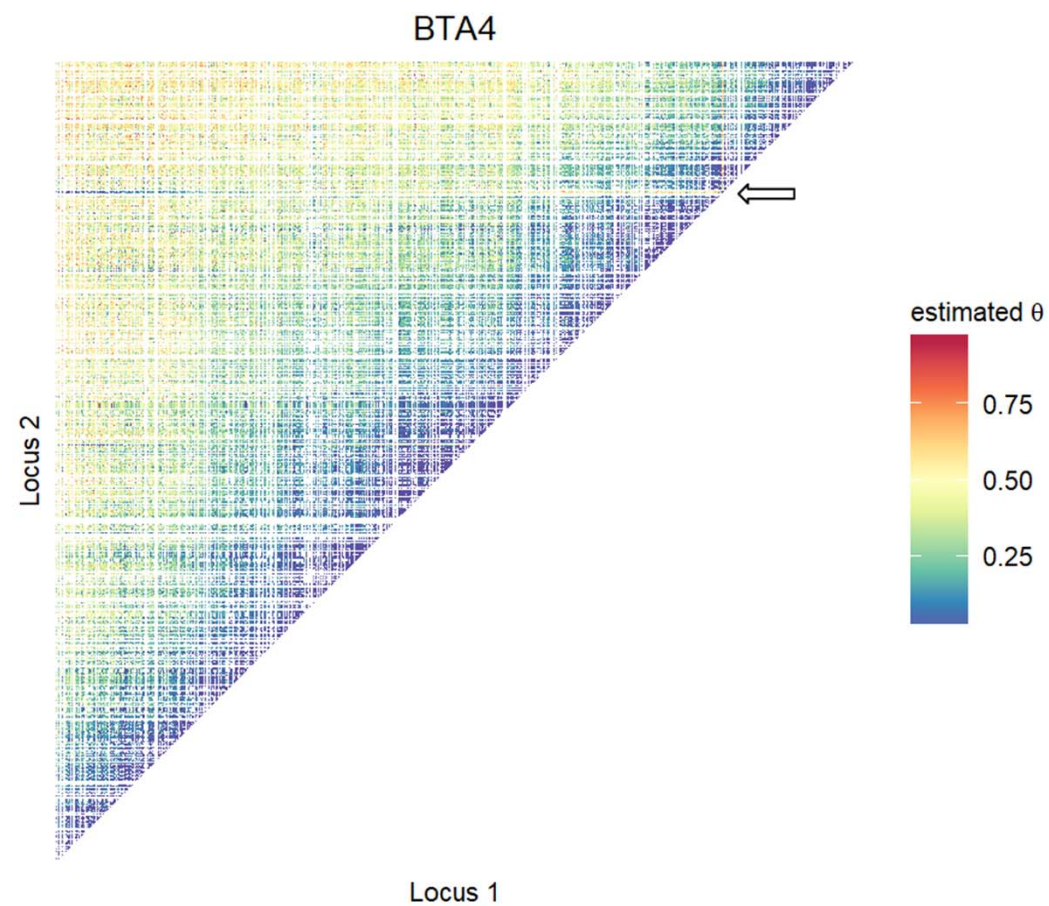

B

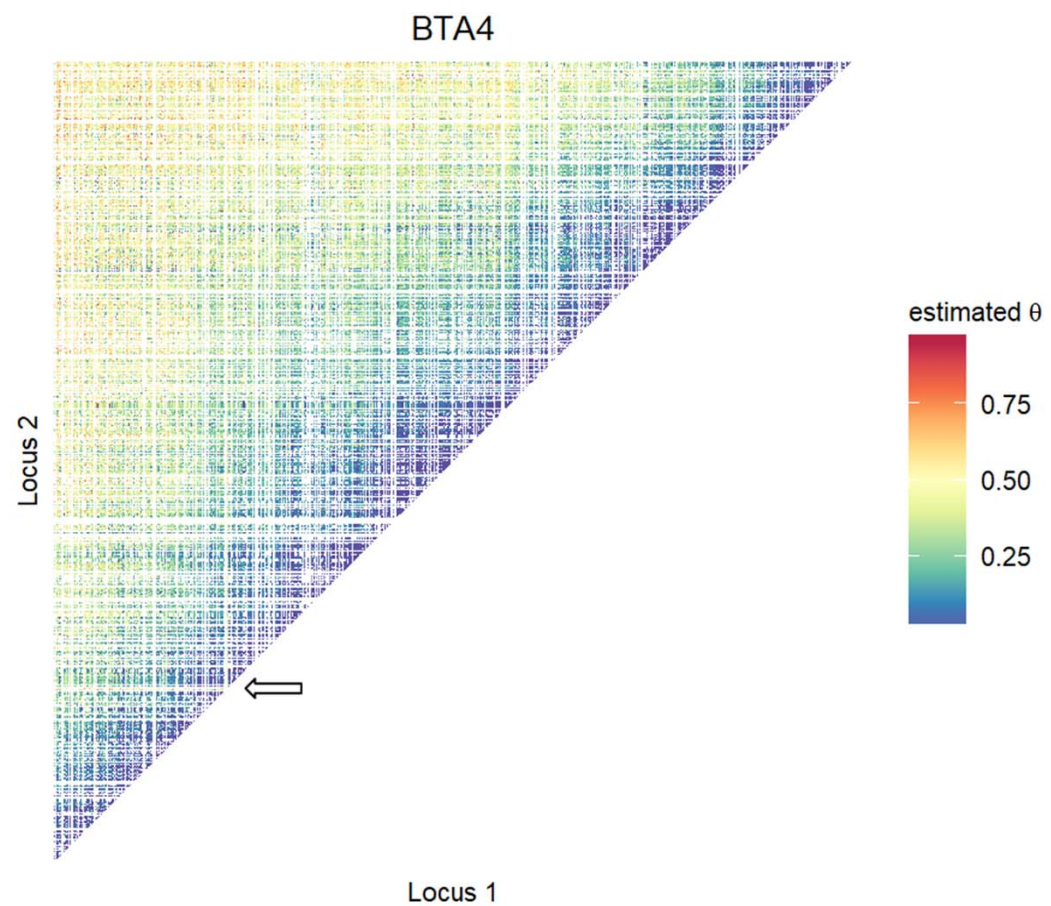

Supplement: Figure S5 — Validation of recombination-rate estimates using empirical bovine data on BTA4. Pairwise recombination rates were obtained using the stepwise procedure EMDP. BTA4 contained SNPs which were misplaced (A) within and (B) between chromosomes. [file Image_5.PDF]
