# Supplementary material for: Estimation of Recombination Rate and Maternal Linkage Disequilibrium in Half-Sibs
Source: Front Genet. 2018 Jun 5;9:186. doi: 10.3389/fgene.2018.00186 (PMC5996054; doi:10.3389/fgene.2018.00186)
Supplement: Figure S2 — Estimates of paternal recombination rate for all autosomes using empirical bovine data. Pairwise recombination rates were obtained using the stepwise procedure EMDP. [file Image_2.PDF]

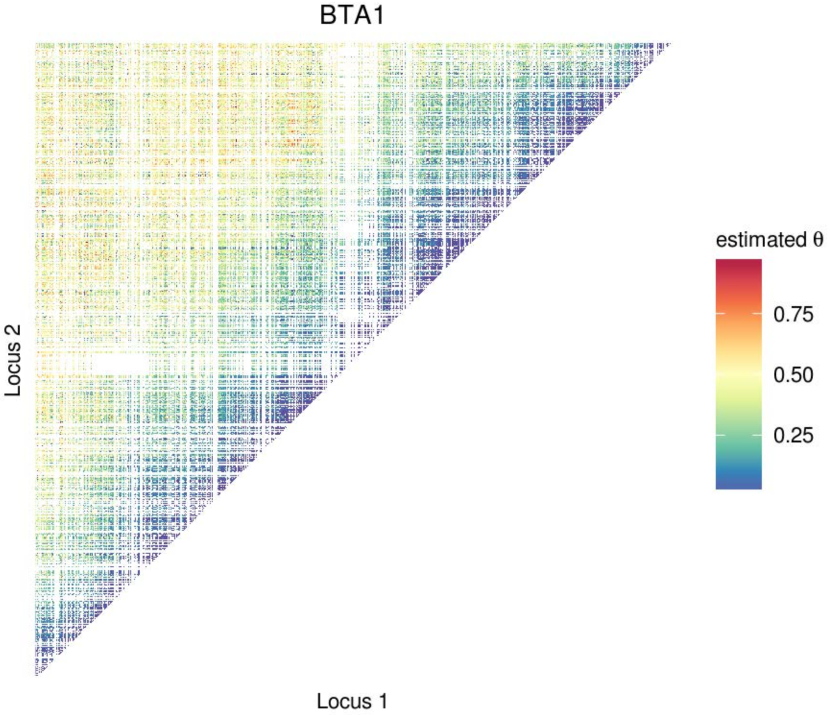

BTA1
Locus 2
Locus 1
estimated θ
0.75
0.50
0.25

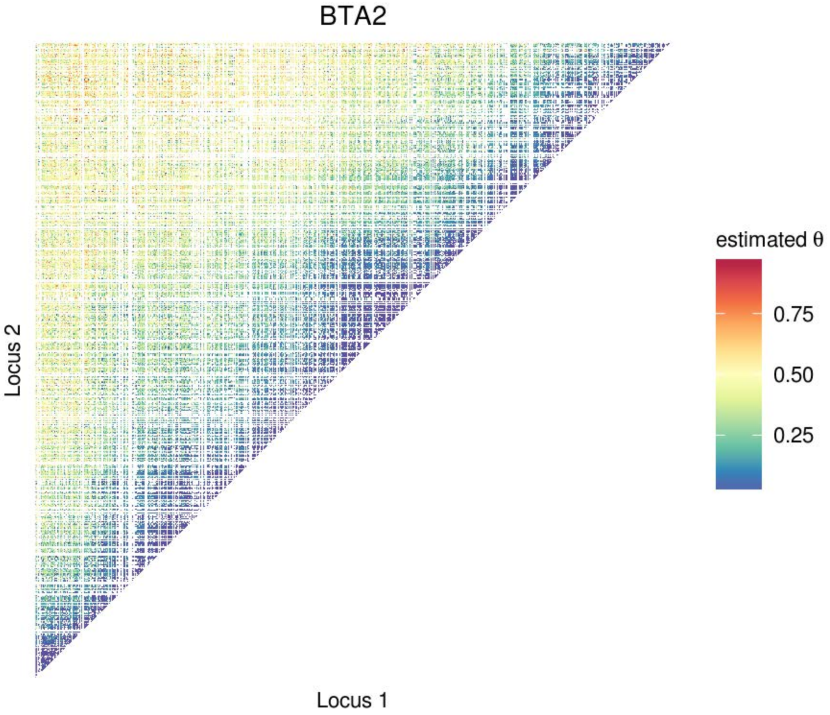

BTA2
Locus 2
Locus 1
estimated θ
0.75
0.50
0.25

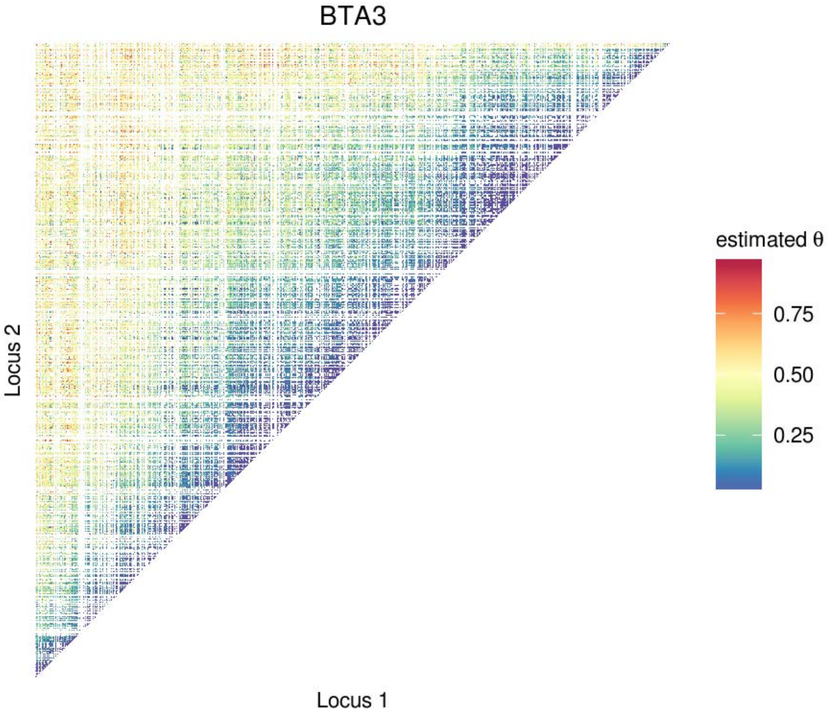
BTA3
estimated θ
0.75
0.50
0.25
Locus 2
Locus 1

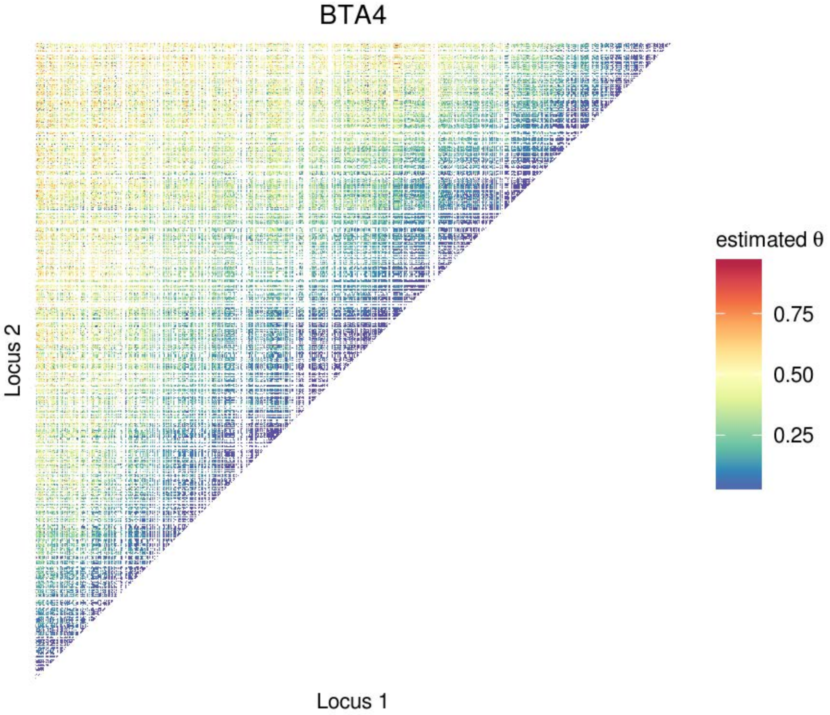
BTA4
estimated θ
0.75
0.50
0.25
Locus 2
Locus 1

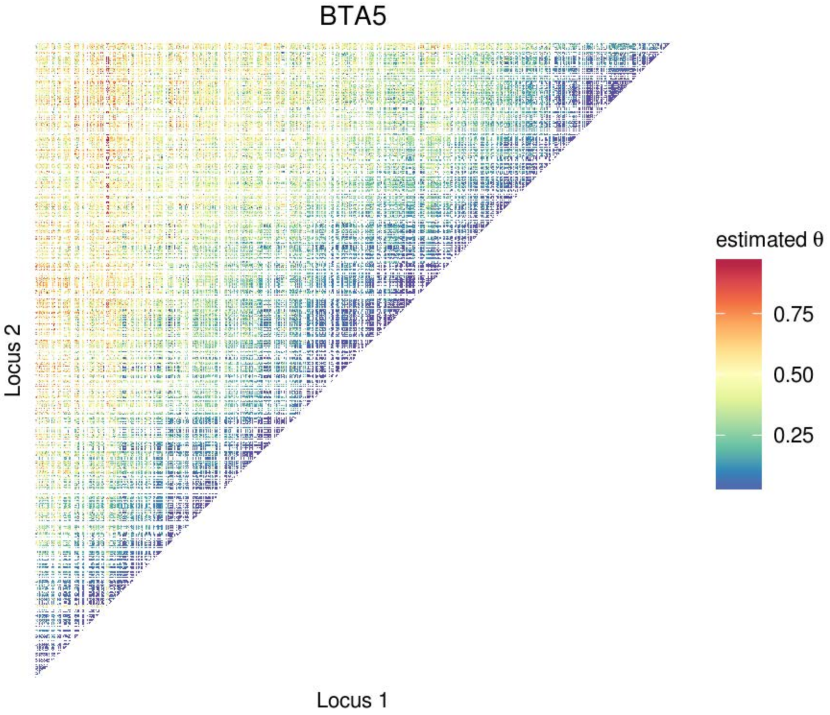
BTA5
Locus 2
Locus 1
estimated θ
0.75
0.50
0.25

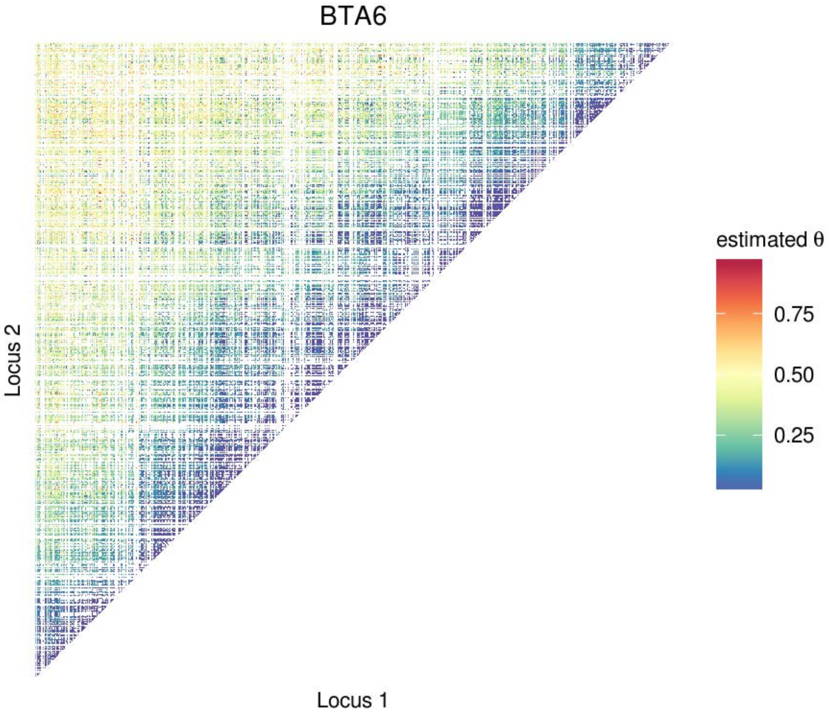
BTA6
Locus 2
Locus 1
estimated θ
0.75
0.50
0.25

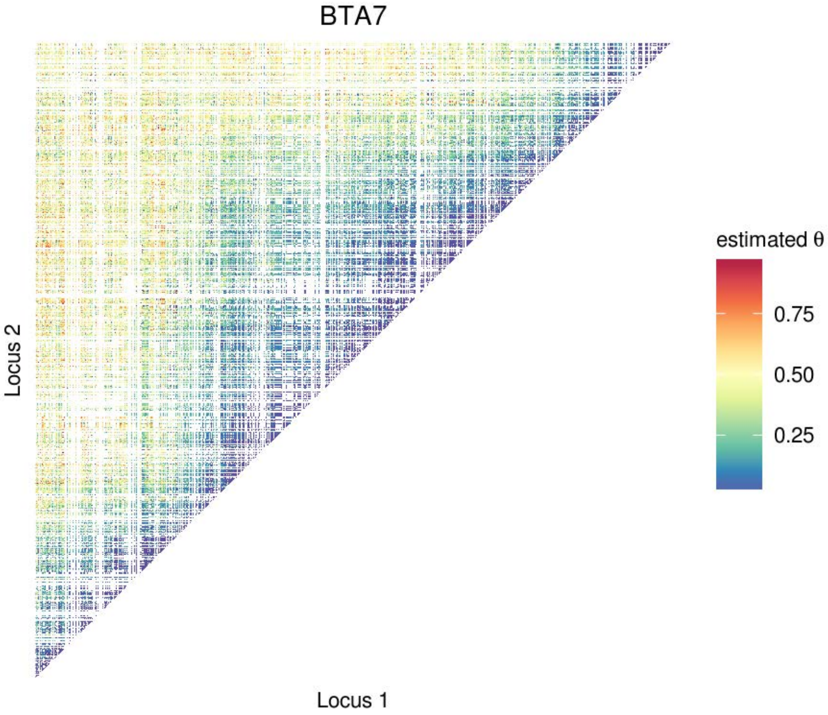
BTA7
Locus 2
Locus 1
estimated θ
0.75
0.50
0.25

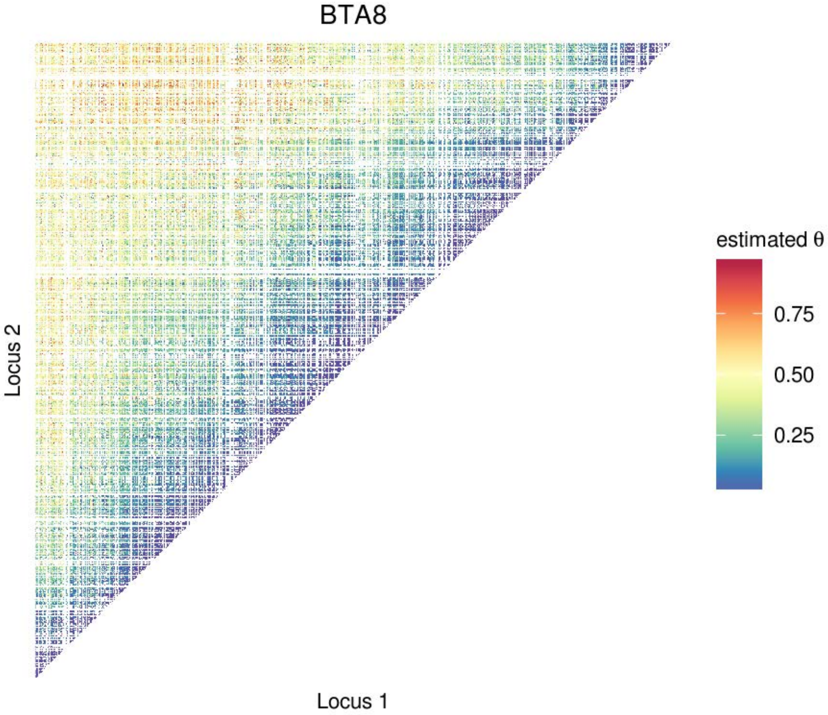
BTA8
Locus 2
Locus 1
estimated θ
0.75
0.50
0.25

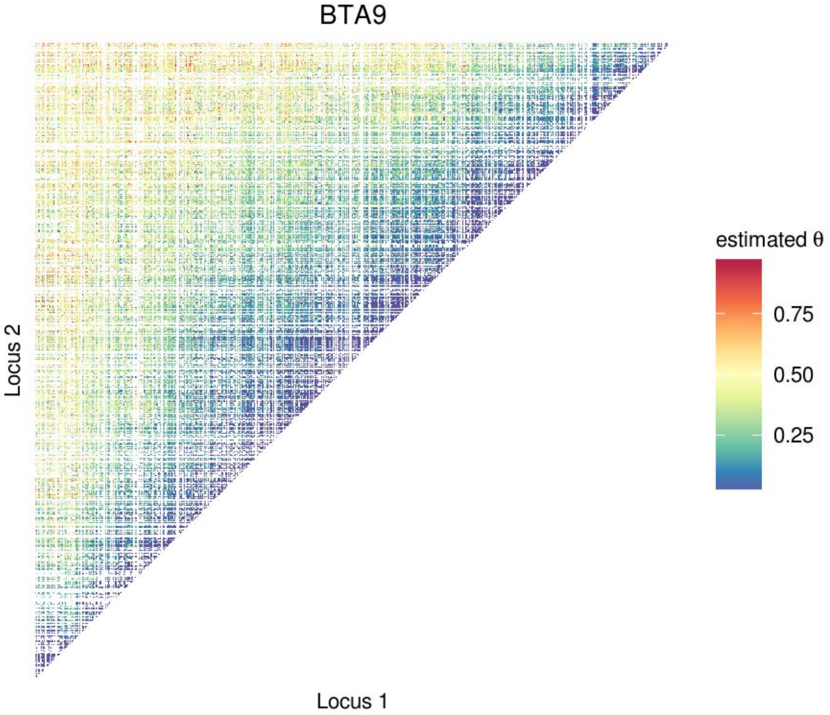
BTA9
Locus 2
Locus 1
estimated θ
0.75
0.50
0.25

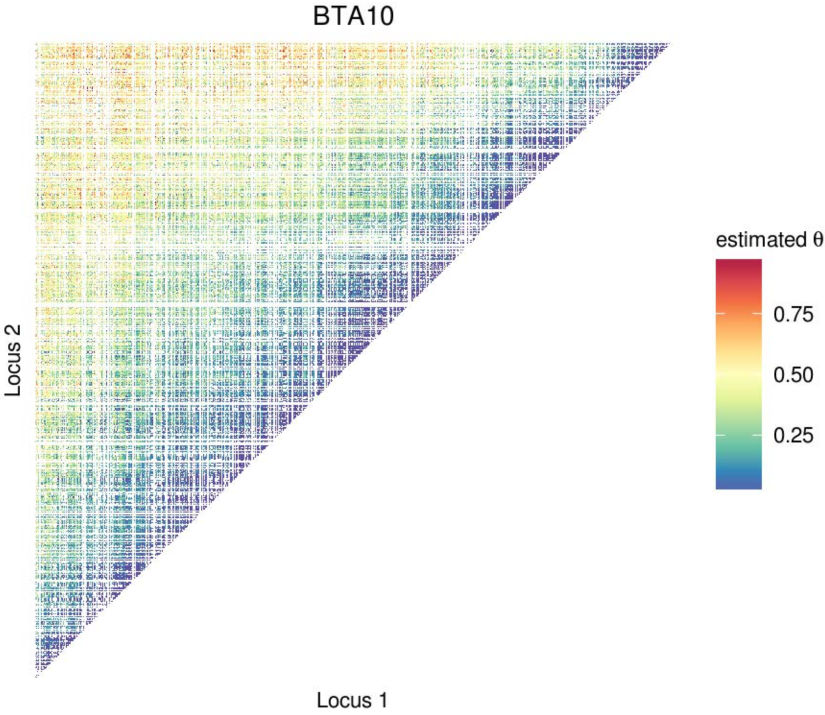
BTA10
estimated θ
0.75
0.50
0.25
Locus 2
Locus 1

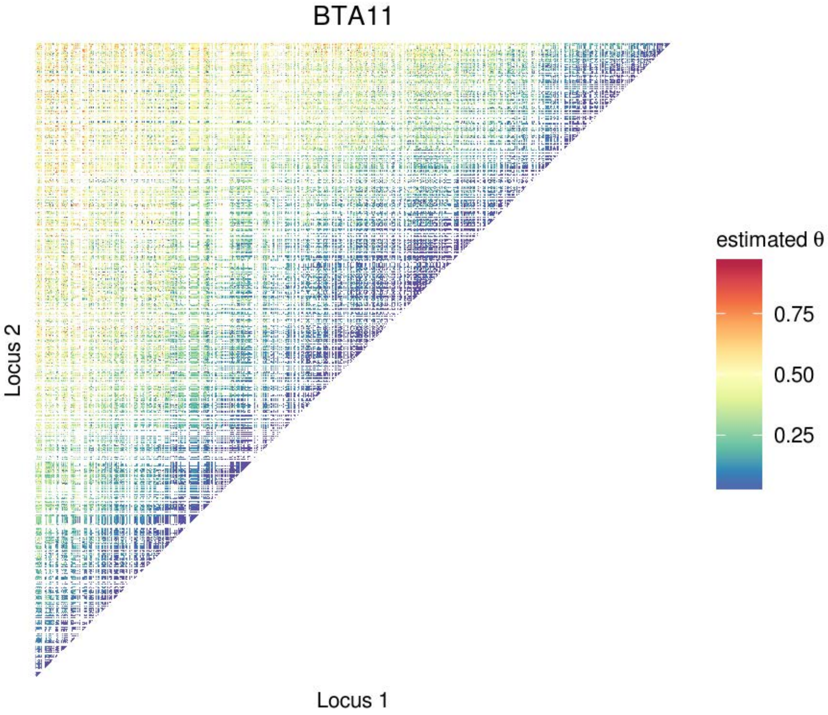
BTA11
Locus 2
Locus 1
estimated θ
0.75
0.50
0.25

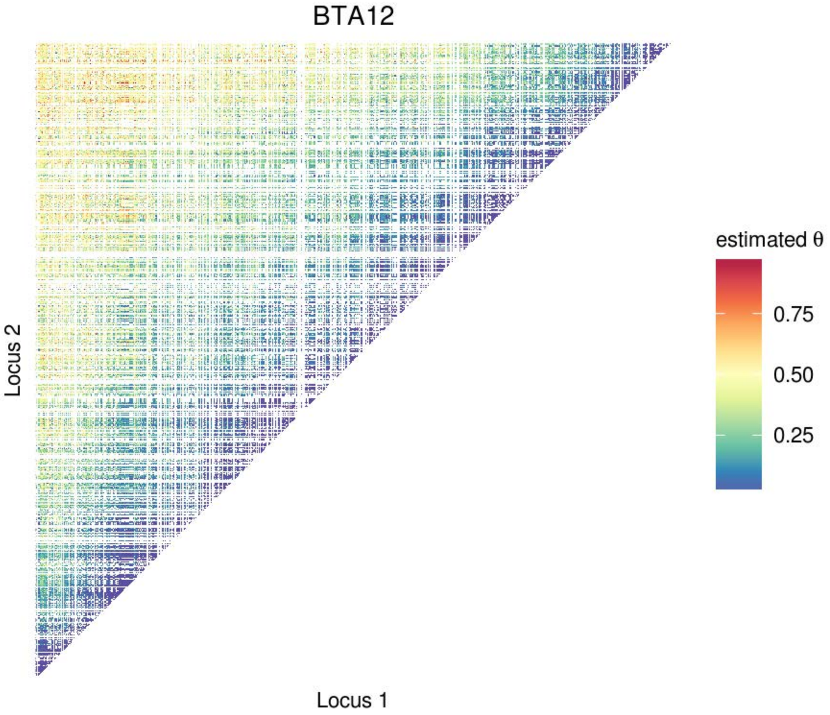
BTA12
Locus 2
Locus 1
estimated θ
0.75
0.50
0.25

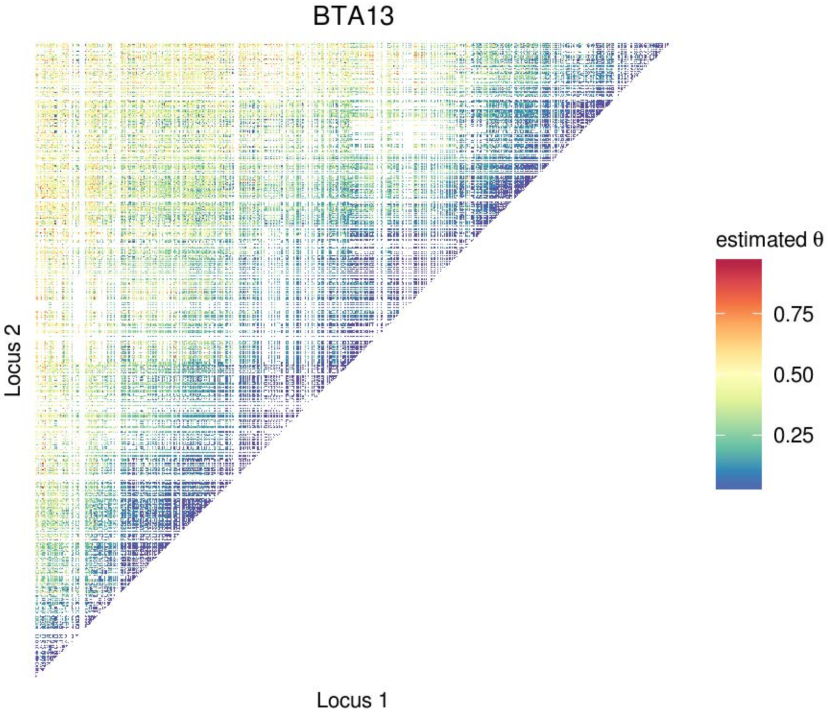
BTA13
Locus 2
Locus 1
estimated θ
0.75
0.50
0.25

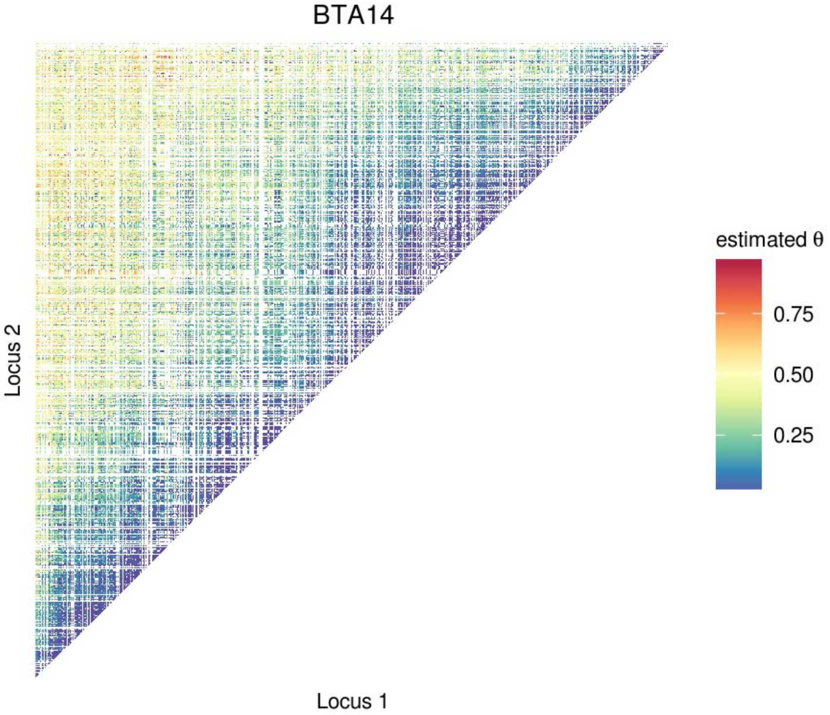
BTA14
Locus 2
Locus 1
estimated θ
0.75
0.50
0.25

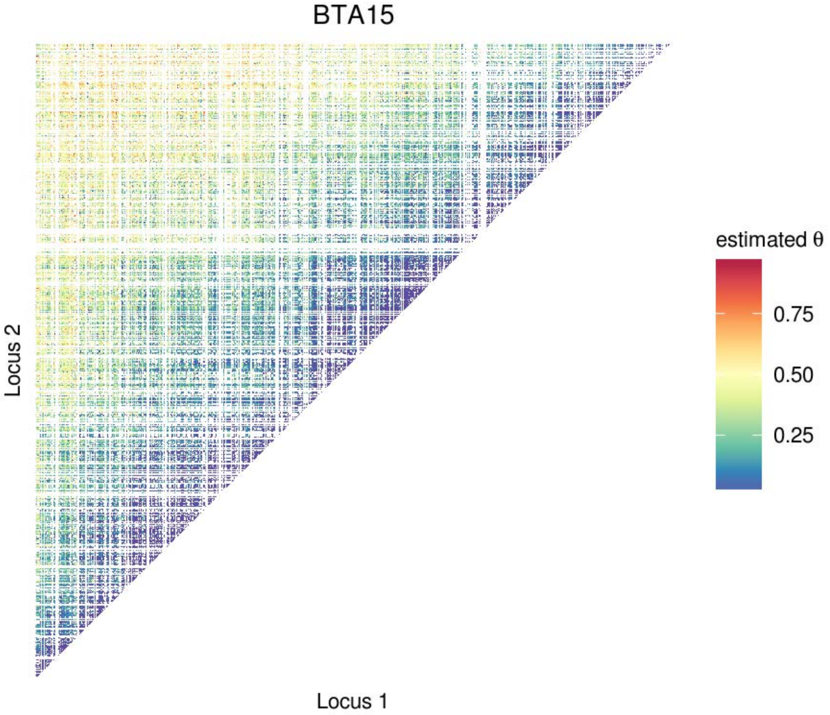
BTA15
estimated θ
0.75
0.50
0.25
Locus 2
Locus 1

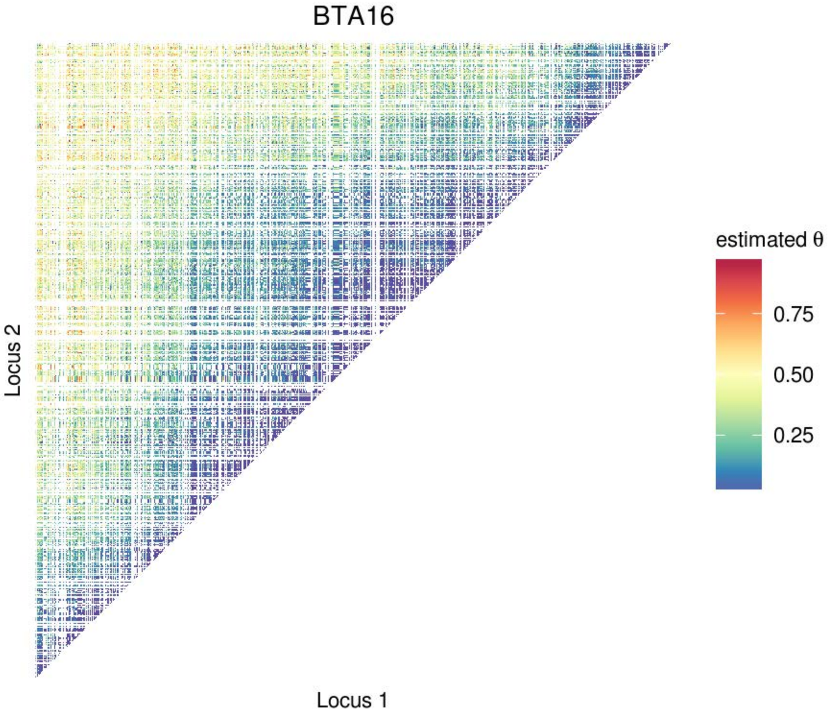
BTA16
Locus 2
Locus 1
estimated θ
0.75
0.50
0.25

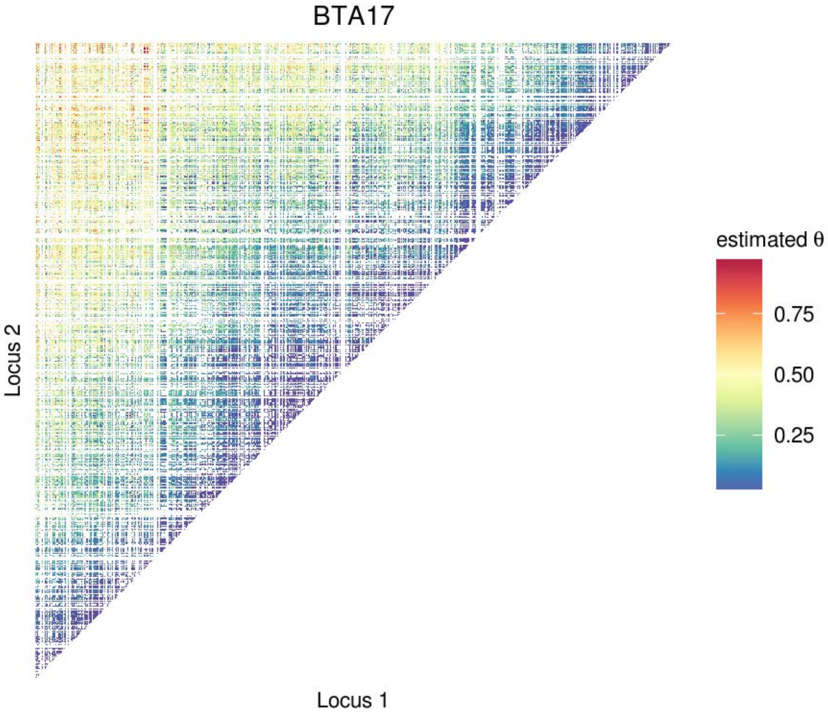
BTA17
Locus 2
Locus 1
estimated θ
0.75
0.50
0.25

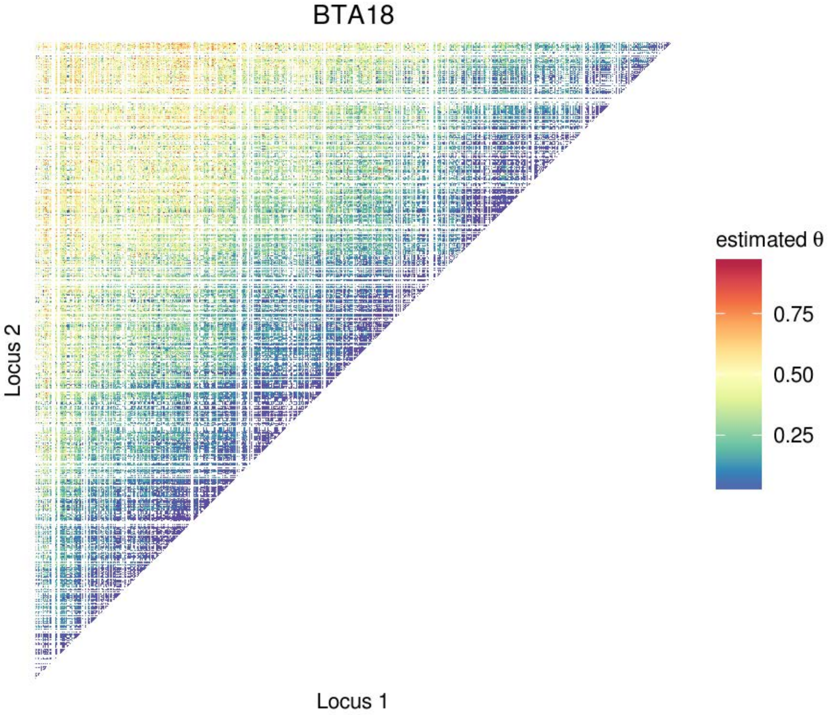
BTA18
estimated θ
0.75
0.50
0.25
Locus 2
Locus 1

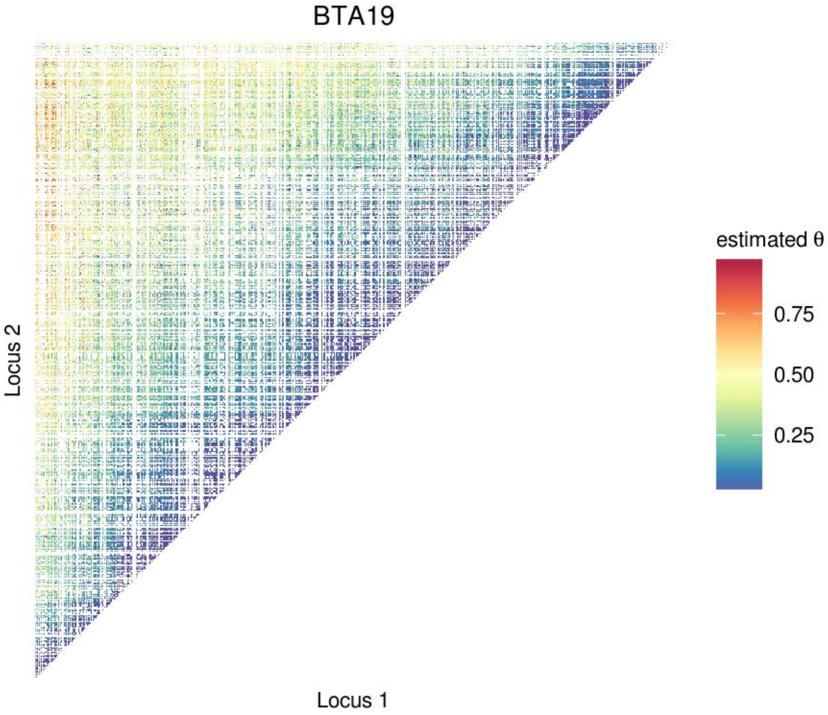
BTA19
Locus 2
Locus 1
estimated θ
0.75
0.50
0.25

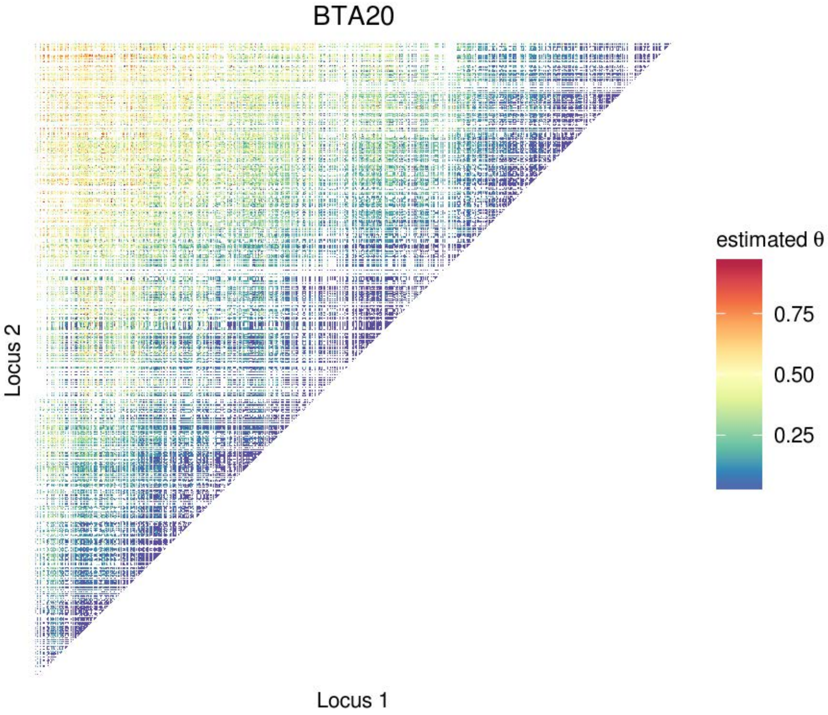
BTA20
estimated θ
0.75
0.50
0.25
Locus 2
Locus 1

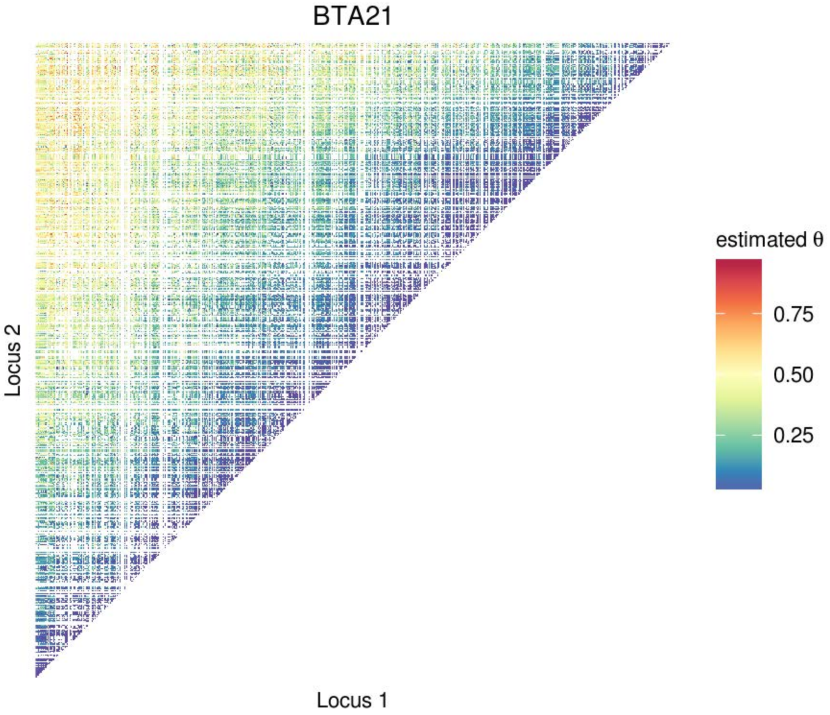
BTA21
Locus 2
Locus 1
estimated θ
0.75
0.50
0.25

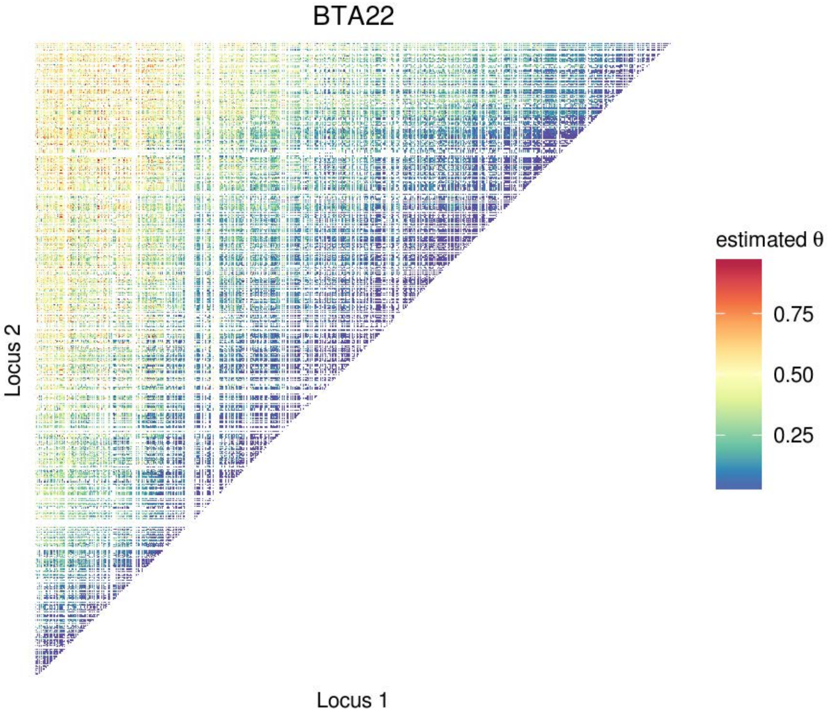
BTA22
Locus 2
Locus 1
estimated θ
0.75
0.50
0.25

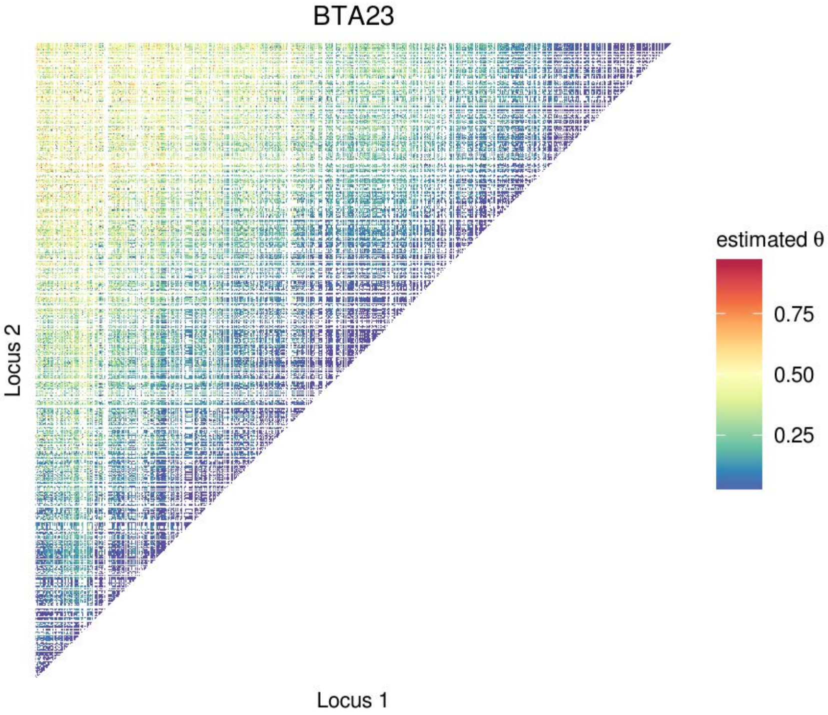
BTA23
Locus 2
Locus 1
estimated θ
0.75
0.50
0.25

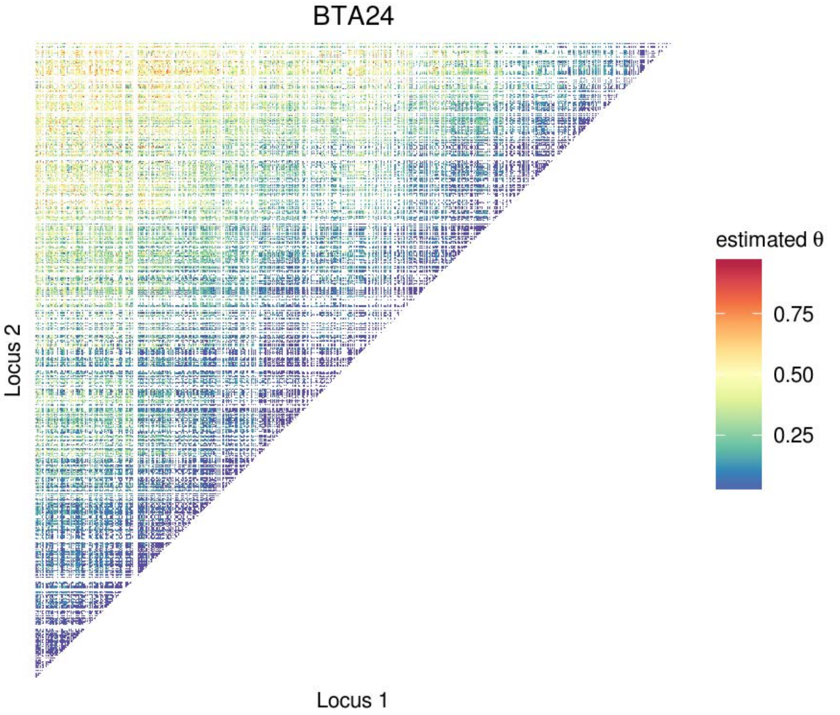
BTA24
Locus 2
Locus 1
estimated θ
0.75
0.50
0.25

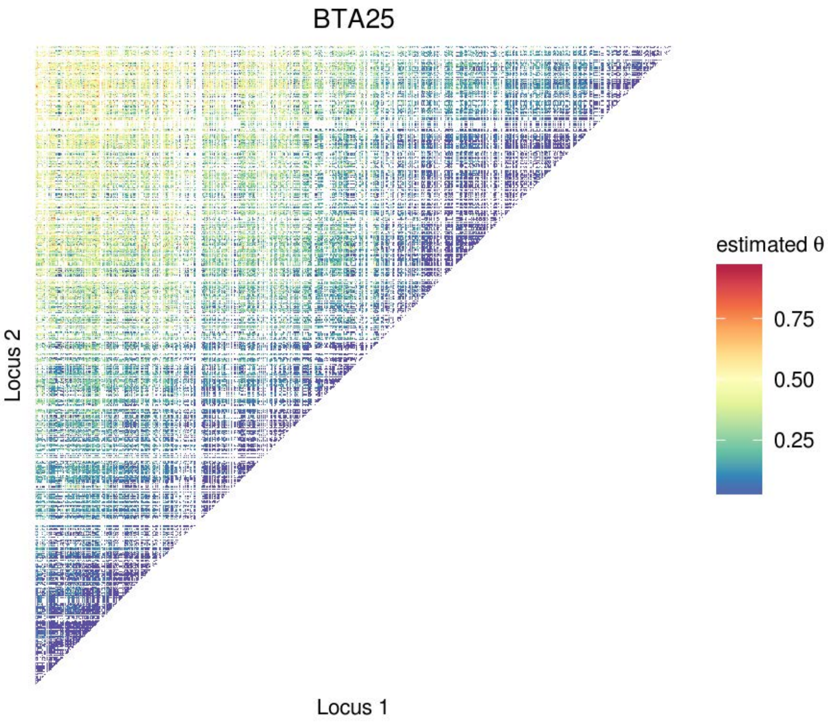
BTA25
estimated θ
0.75
0.50
0.25
Locus 2
Locus 1

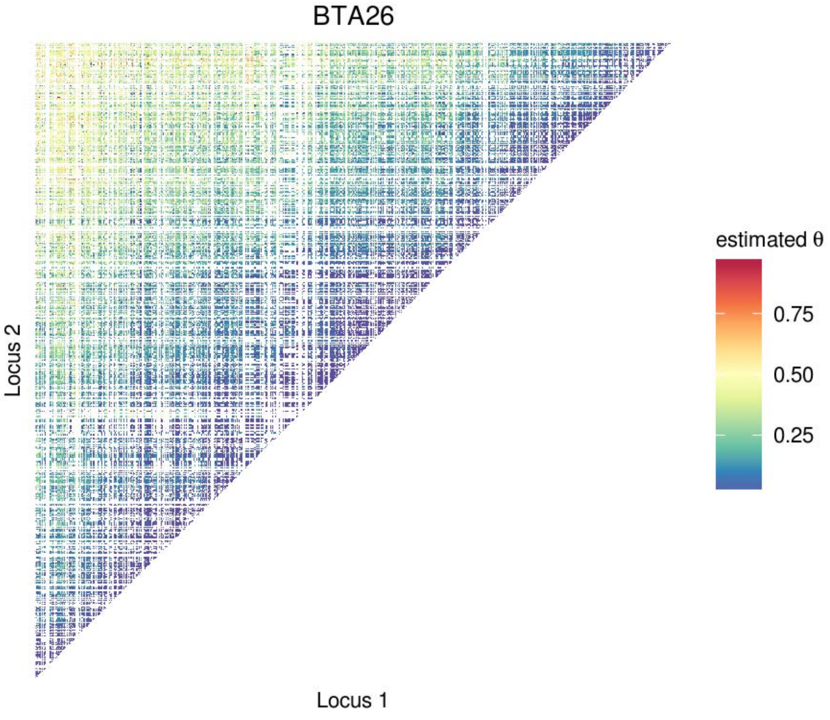
BTA26
estimated θ
0.75
0.50
0.25
Locus 2
Locus 1

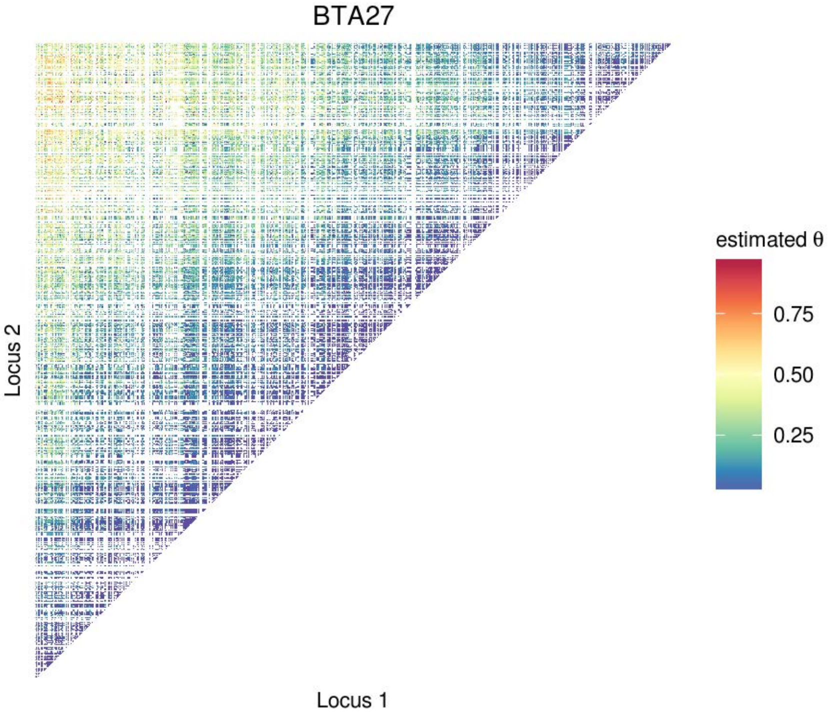
BTA27
Locus 2
Locus 1
estimated θ
0.75
0.50
0.25

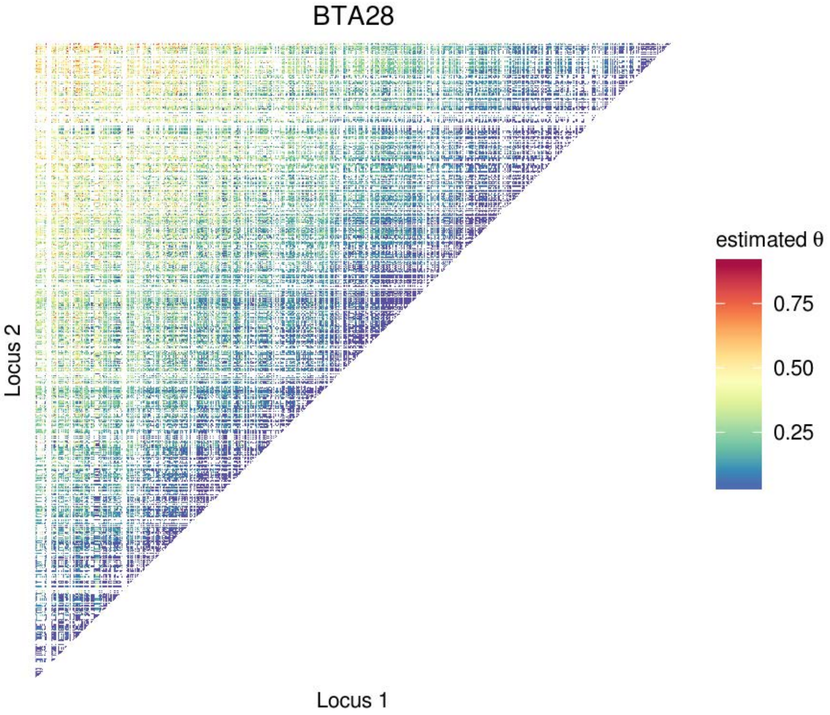
BTA28
Locus 2
Locus 1
estimated θ
0.75
0.50
0.25

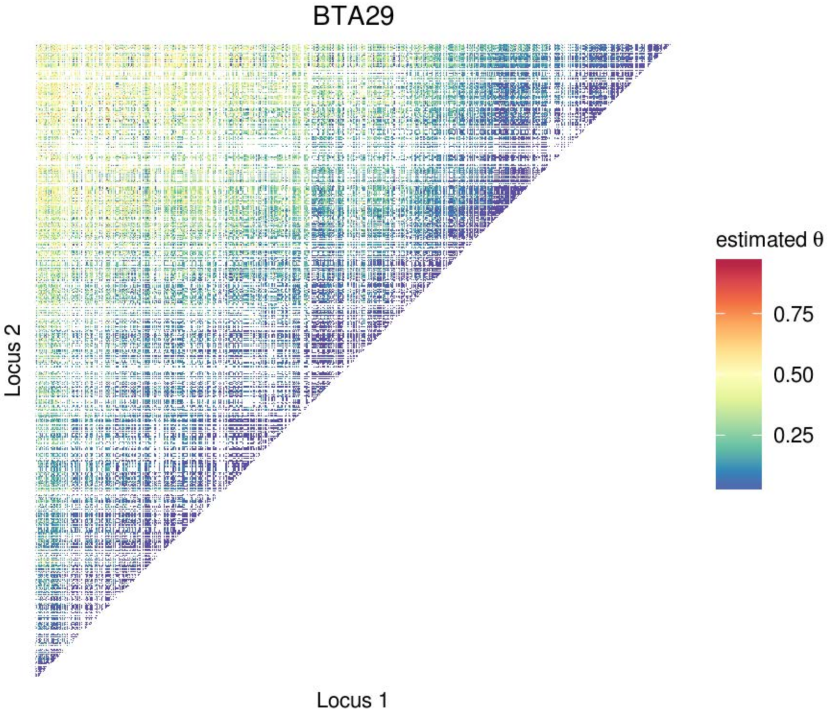
BTA29
Locus 2
Locus 1
estimated θ
0.75
0.50
0.25
