# Supplementary material for: Estimation of Recombination Rate and Maternal Linkage Disequilibrium in Half-Sibs
Source: Front Genet. 2018 Jun 5;9:186. doi: 10.3389/fgene.2018.00186 (PMC5996054; doi:10.3389/fgene.2018.00186)
Supplement: Figure S3 — Estimates of maternal linkage disequilibrium for all autosomes using empirical bovine data. Pairwise linkage disequilibria were obtained using the stepwise procedure EMDP. [file Image_3.PDF]

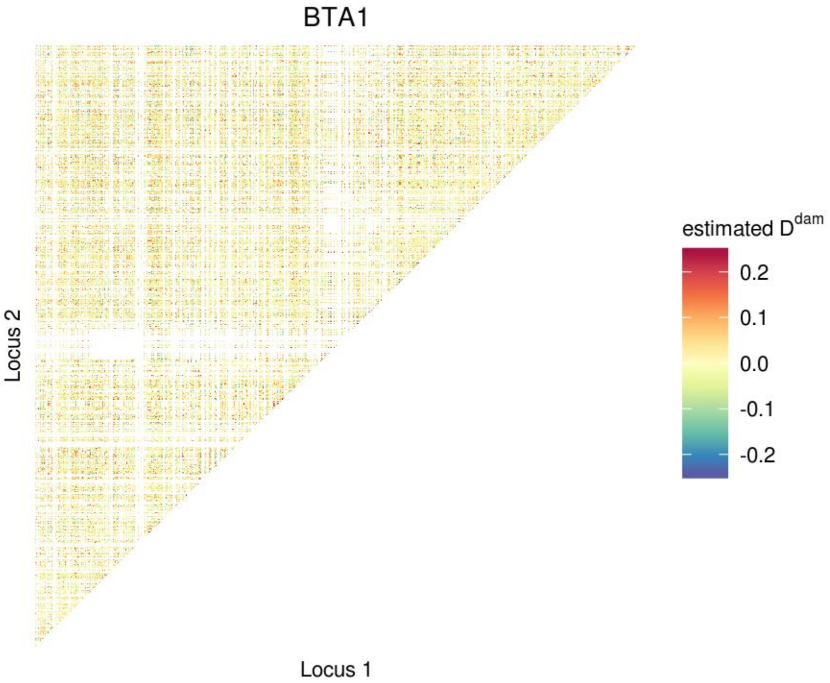
BTA1
Locus 2
Locus 1
estimated D^{dam}
0.2
0.1
0.0
-0.1
-0.2

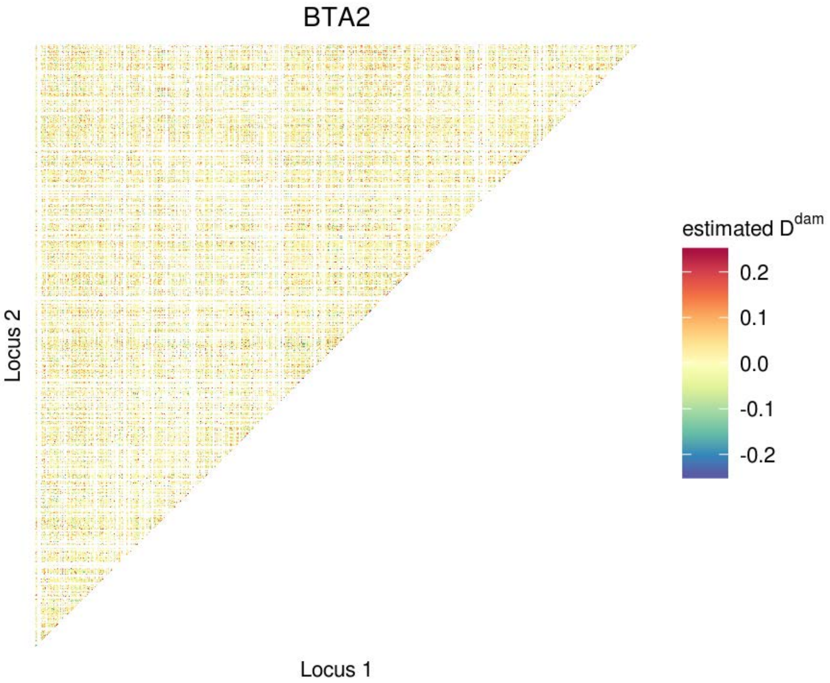
BTA2
Locus 2
Locus 1
estimated D^{dam}
0.2
0.1
0.0
-0.1
-0.2

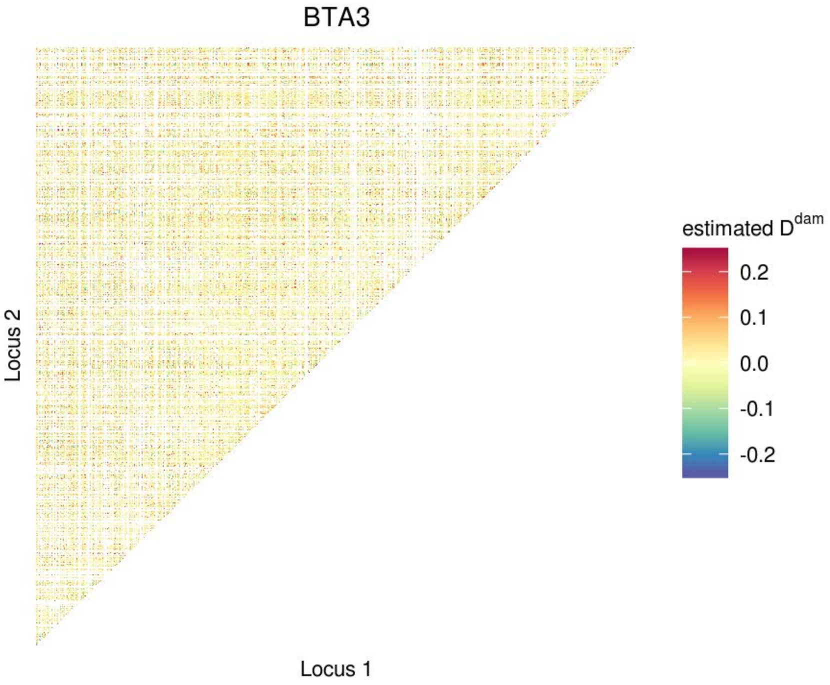
BTA3
Locus 2
Locus 1
estimated D^{dam}
0.2
0.1
0.0
-0.1
-0.2

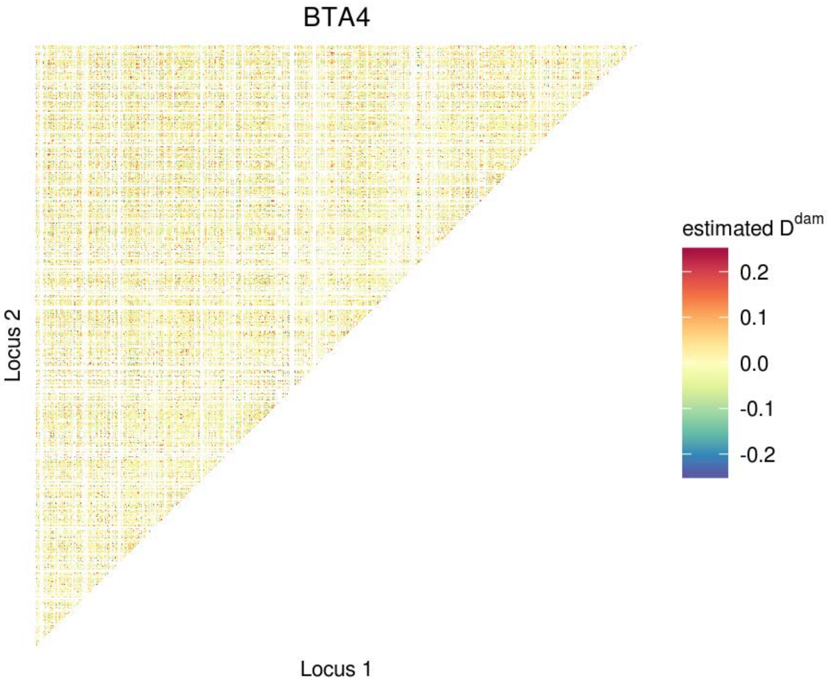

BTA4
Locus 2
Locus 1
estimated D^{dam}
0.2
0.1
0.0
-0.1
-0.2

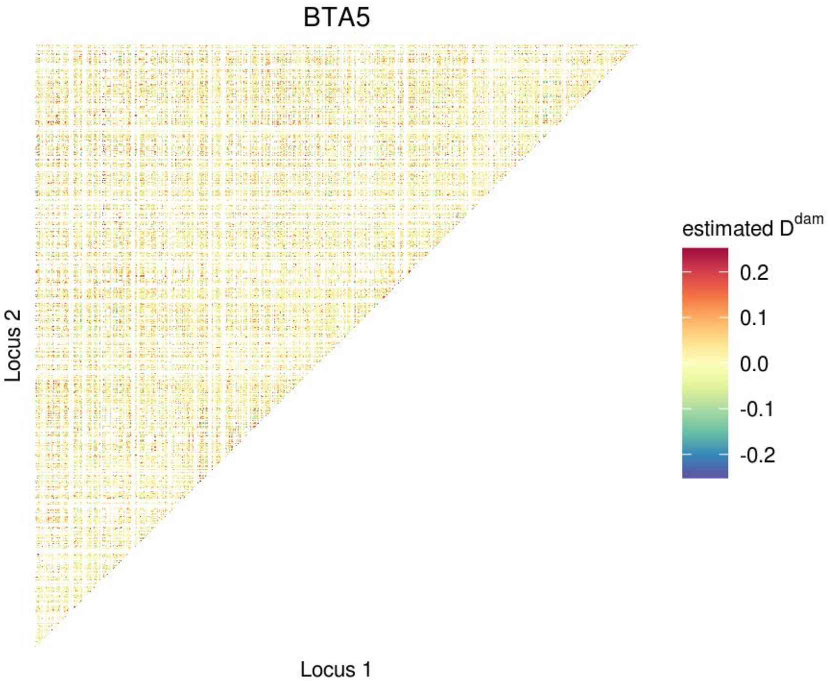
BTA5
Locus 2
Locus 1
estimated D^{dam}
0.2
0.1
0.0
-0.1
-0.2

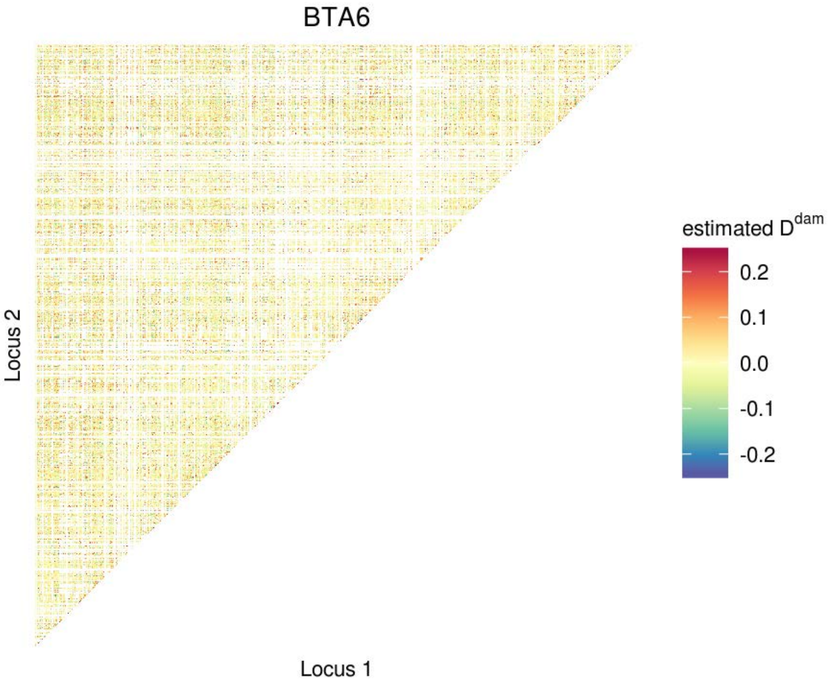

BTA6
Locus 2
Locus 1
estimated D^{dam}
0.2
0.1
0.0
-0.1
-0.2

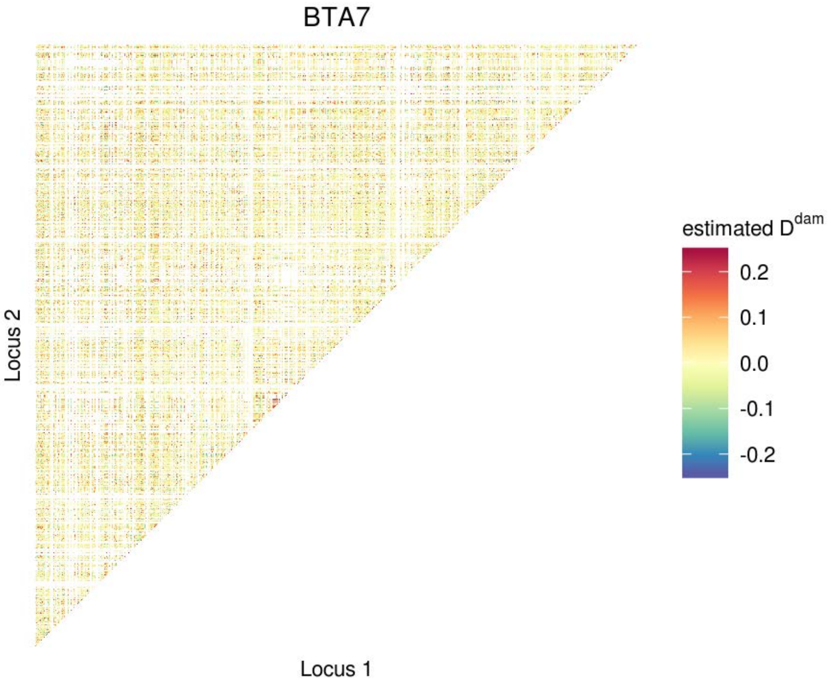

BTA7
Locus 2
Locus 1
estimated D^{dam}
0.2
0.1
0.0
-0.1
-0.2

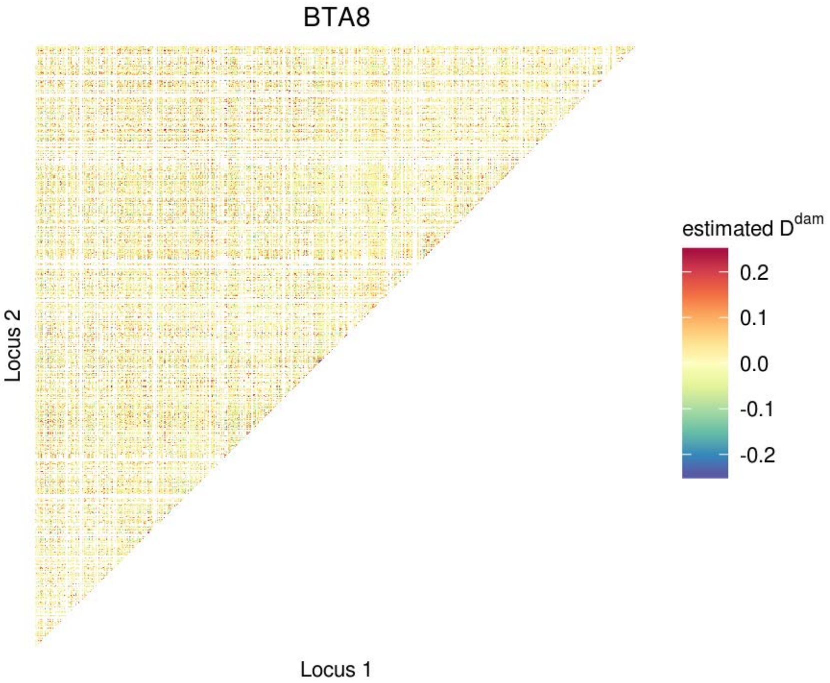

BTA8
Locus 2
Locus 1
estimated D^{dam}
0.2
0.1
0.0
-0.1
-0.2

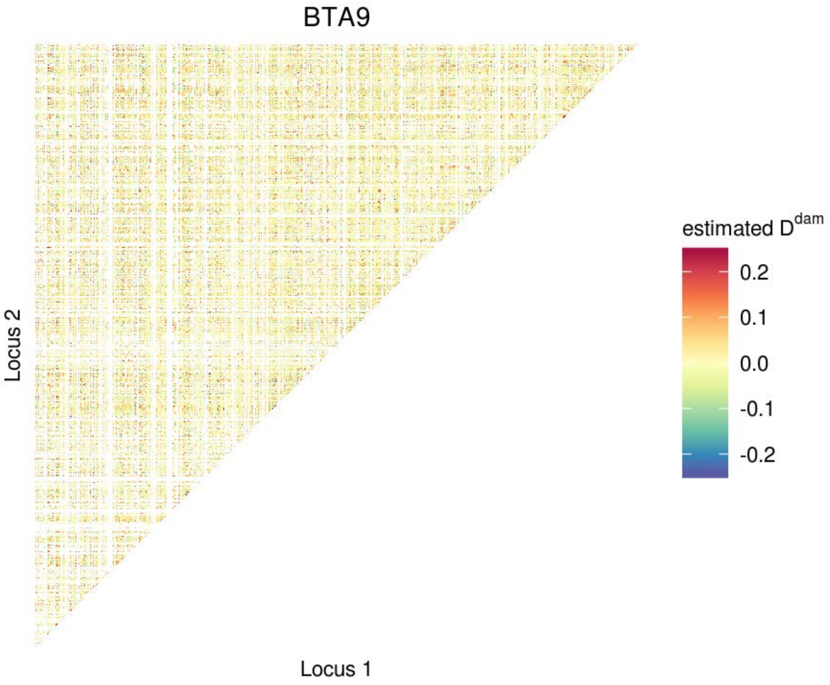
BTA9
Locus 2
Locus 1
estimated D^{dam}
0.2
0.1
0.0
-0.1
-0.2

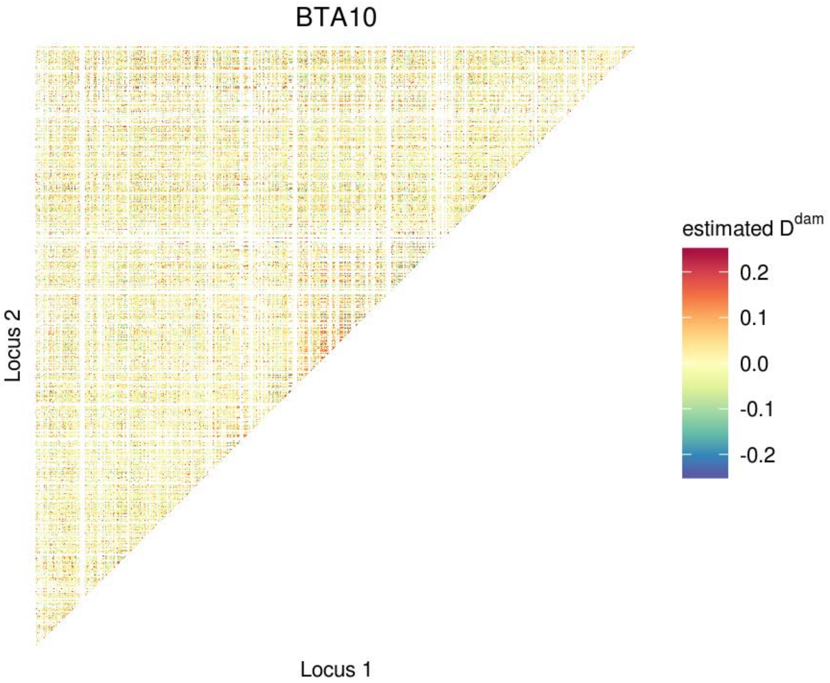

BTA10
Locus 2
Locus 1
estimated D^{dam}
0.2
0.1
0.0
-0.1
-0.2

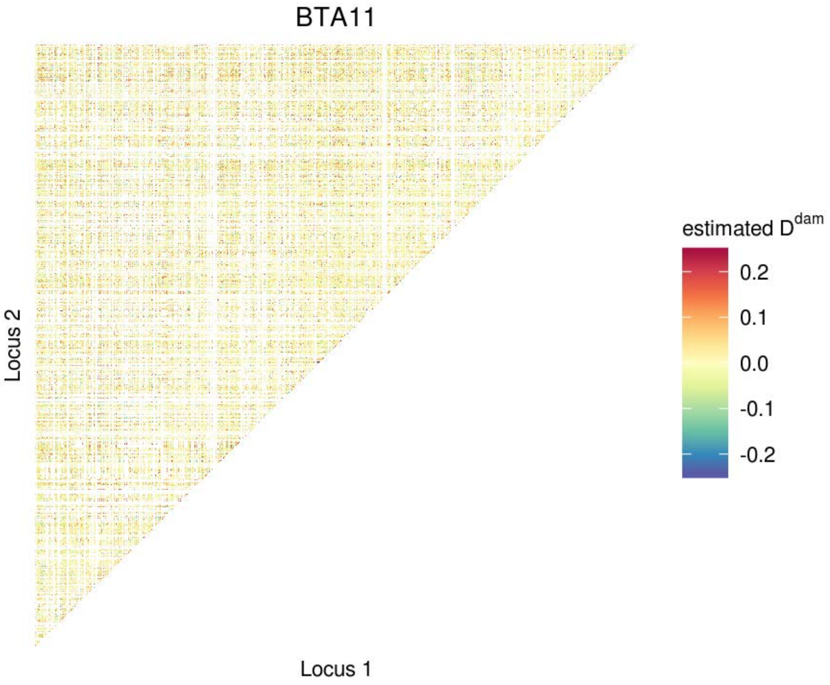
BTA11
Locus 2
Locus 1
estimated D^dam
0.2
0.1
0.0
-0.1
-0.2

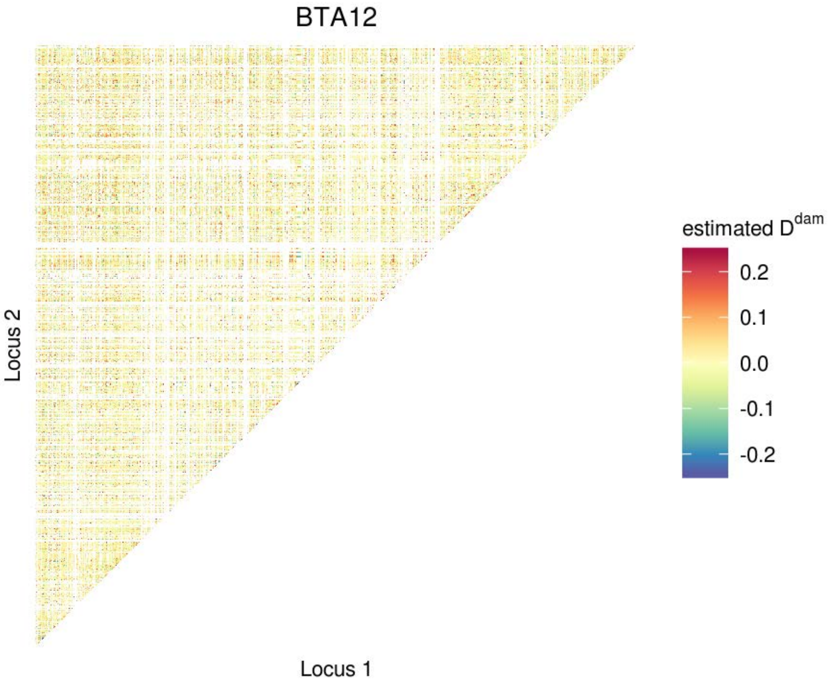

BTA12
Locus 2
Locus 1
estimated D^{dam}
0.2
0.1
0.0
-0.1
-0.2

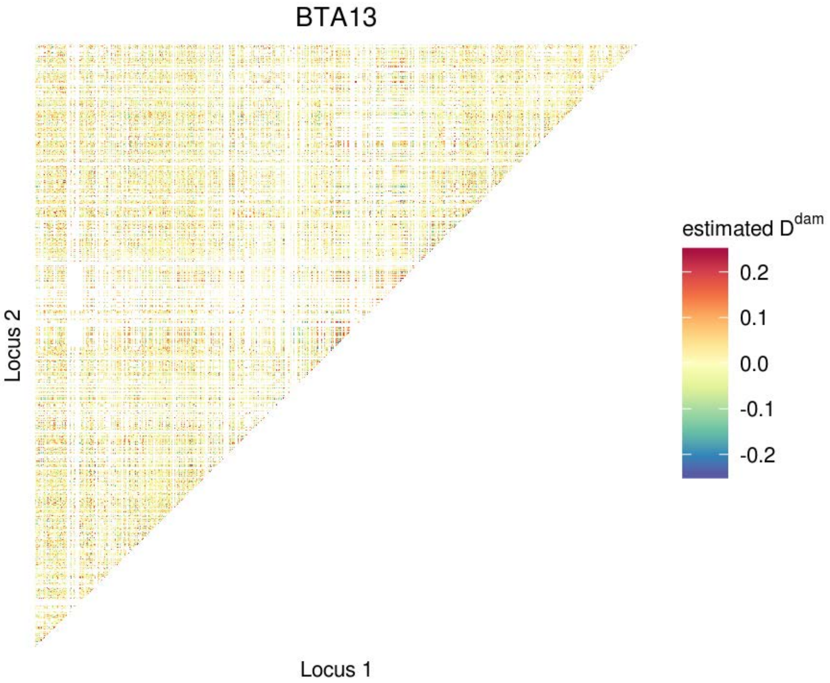
BTA13
Locus 2
Locus 1
estimated D^{dam}
0.2
0.1
0.0
-0.1
-0.2

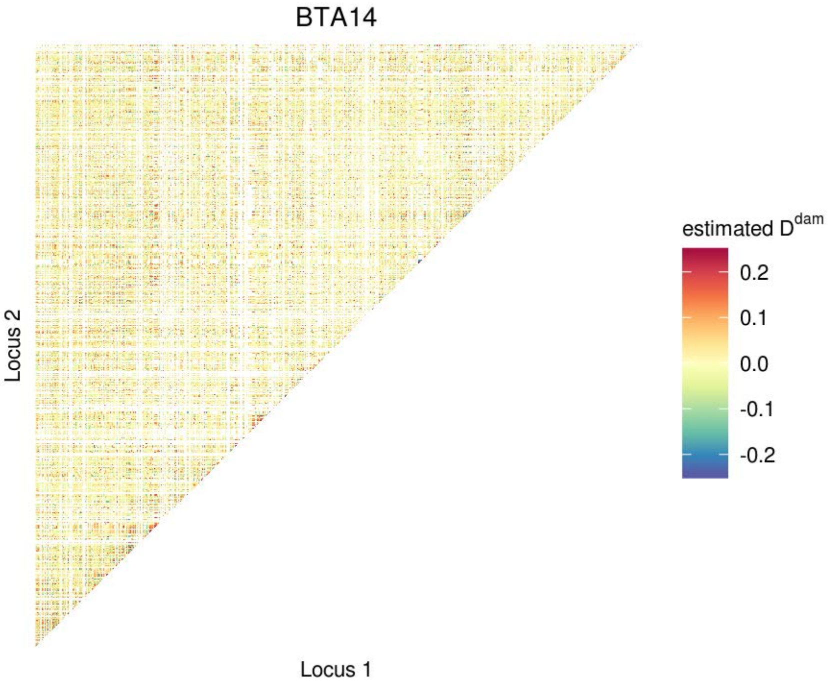
BTA14
Locus 2
Locus 1
estimated D^{dam}
0.2
0.1
0.0
-0.1
-0.2

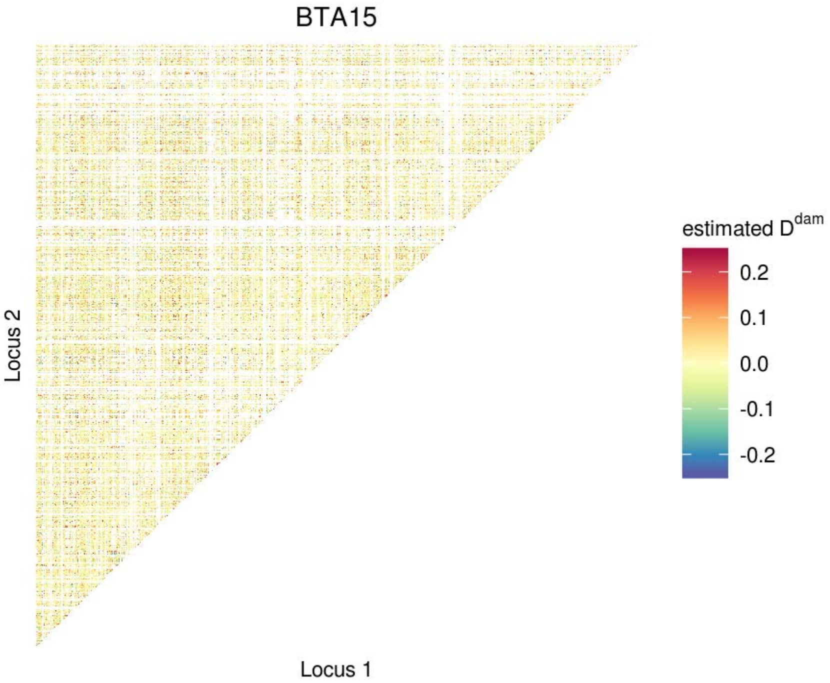
BTA15
Locus 2
Locus 1
estimated D^{dam}
0.2
0.1
0.0
-0.1
-0.2

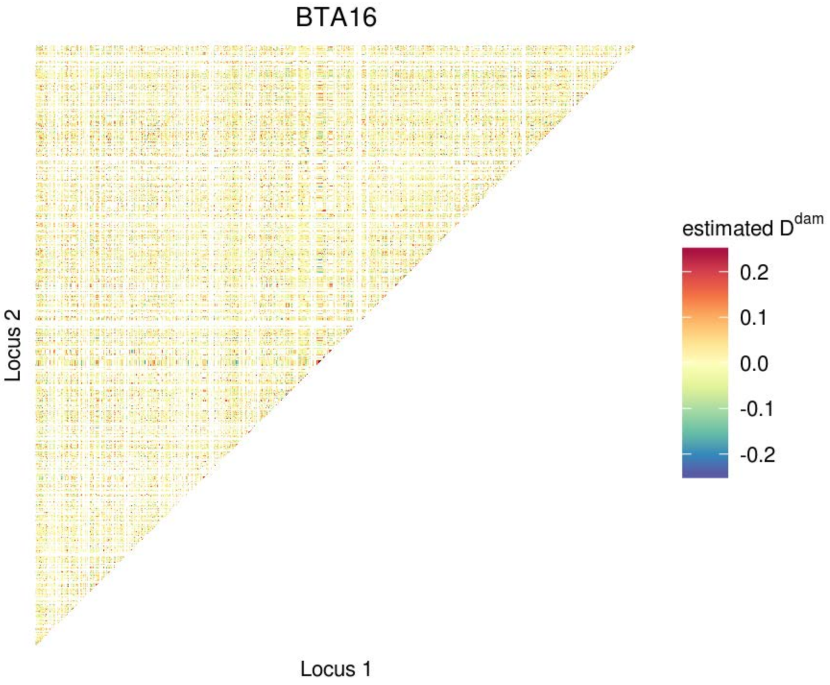

BTA16
estimated D^{dam}
0.2
0.1
0.0
-0.1
-0.2
Locus 2
Locus 1

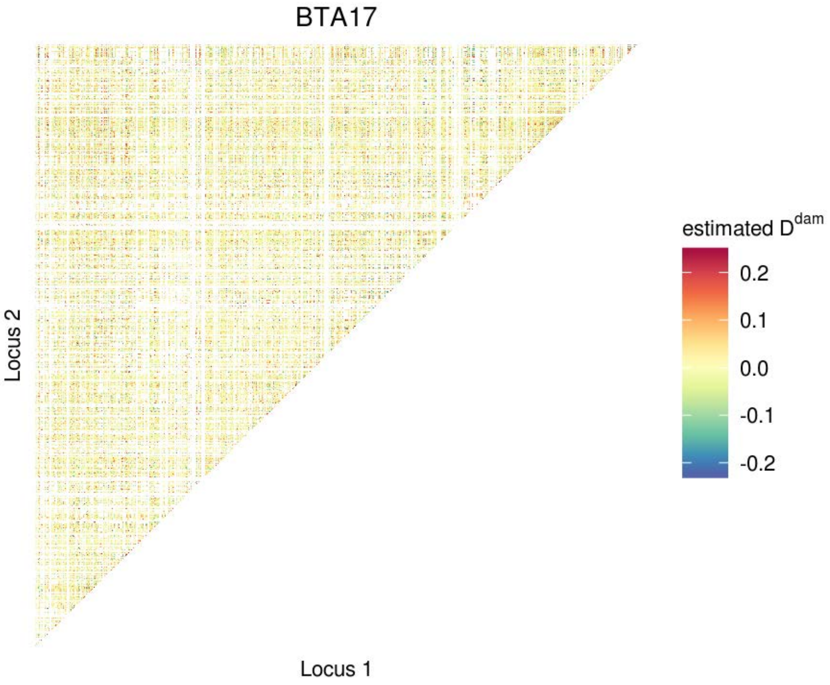
BTA17
Locus 2
Locus 1
estimated D^{dam}
0.2
0.1
0.0
-0.1
-0.2

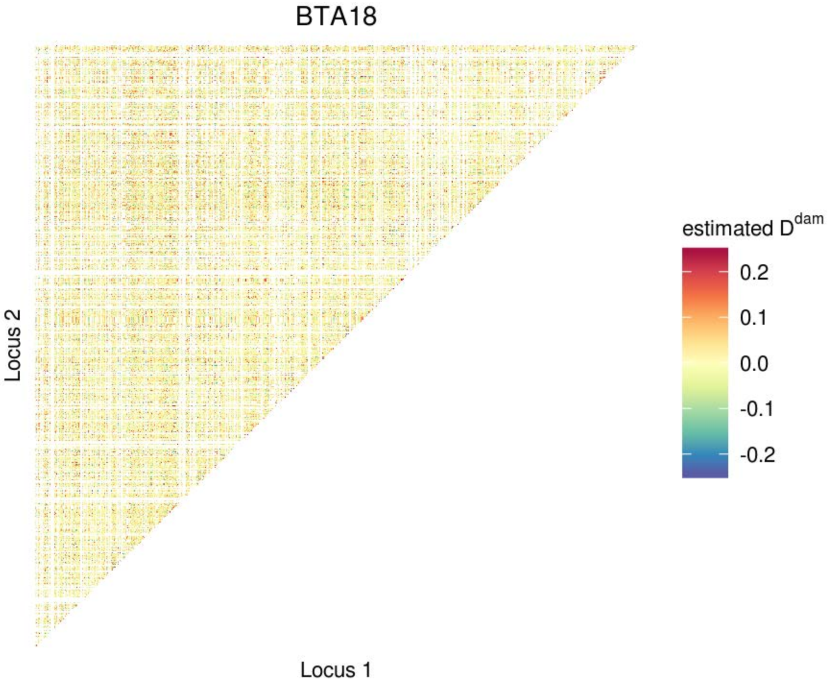
BTA18
Locus 2
Locus 1
estimated D^dam
0.2
0.1
0.0
-0.1
-0.2

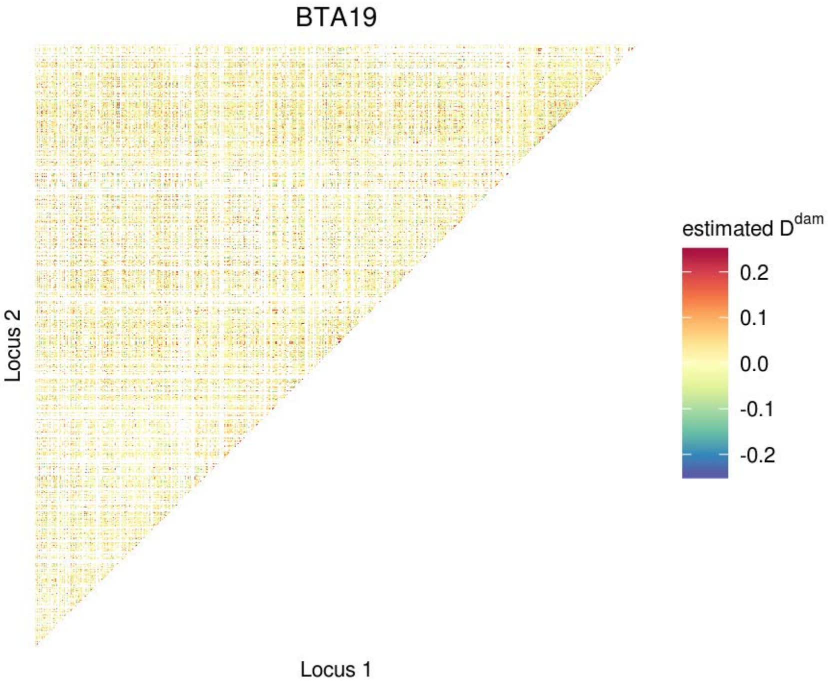
BTA19
Locus 2
Locus 1
estimated D^dam
0.2
0.1
0.0
-0.1
-0.2

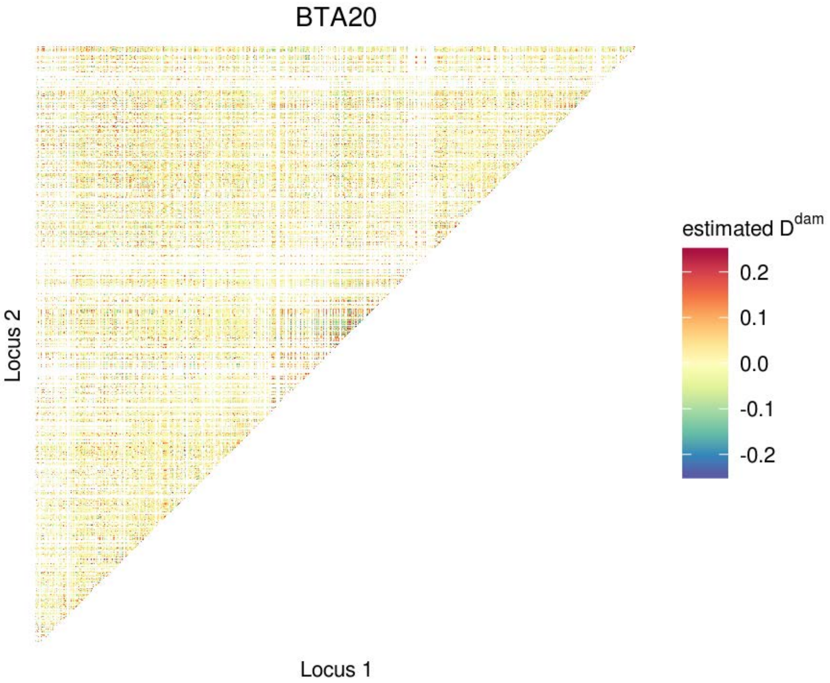
BTA20
Locus 2
Locus 1
estimated D^{dam}
0.2
0.1
0.0
-0.1
-0.2

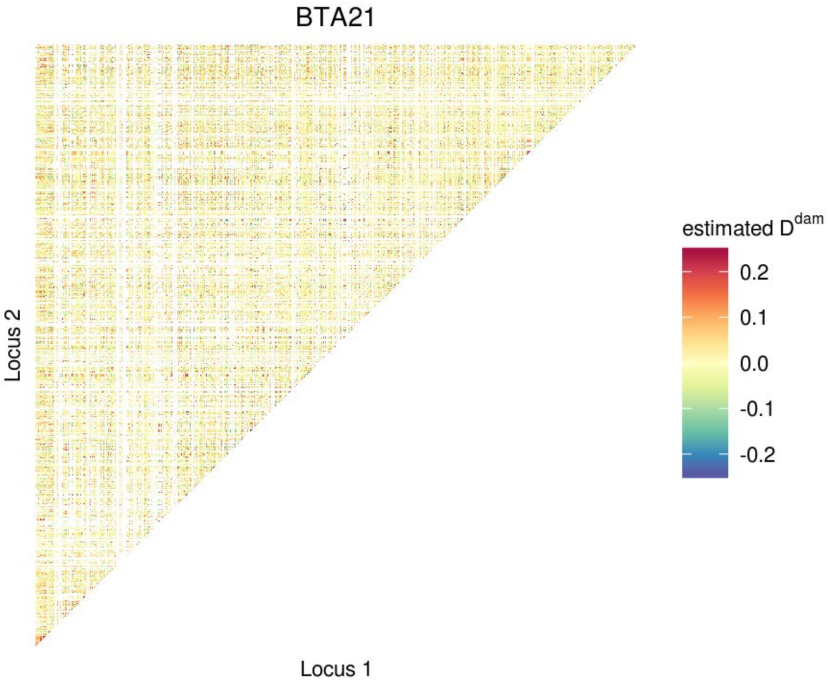

BTA21
Locus 2
Locus 1
estimated D^dam
0.2
0.1
0.0
-0.1
-0.2

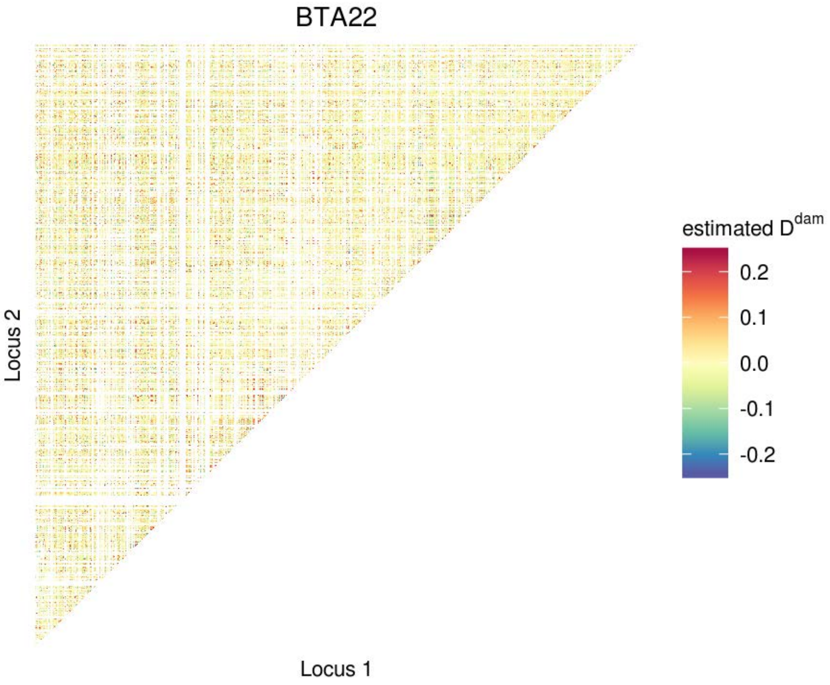
BTA22
estimated D^{dam}
0.2
0.1
0.0
-0.1
-0.2
Locus 2
Locus 1

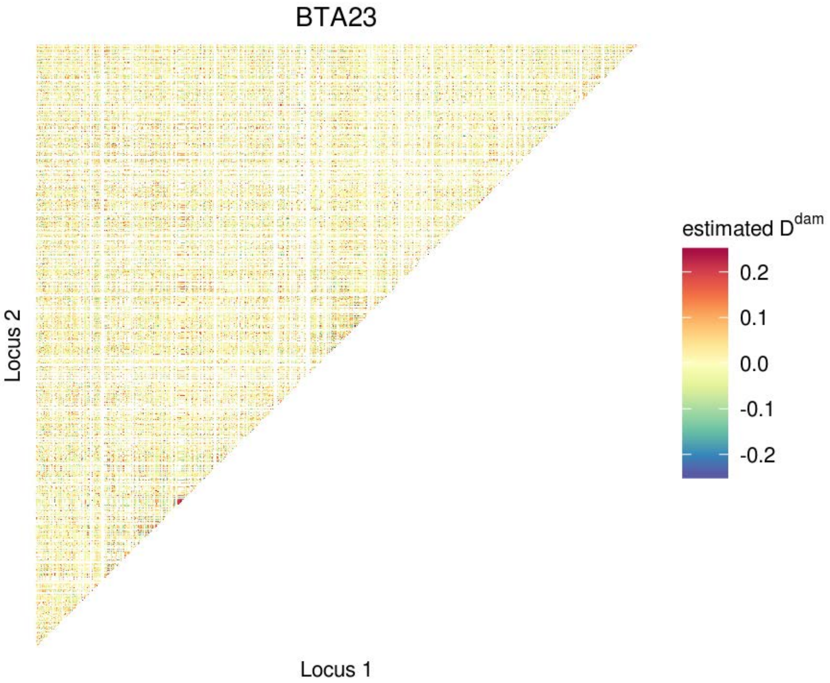

BTA23
Locus 2
Locus 1
estimated D^{dam}
0.2
0.1
0.0
-0.1
-0.2

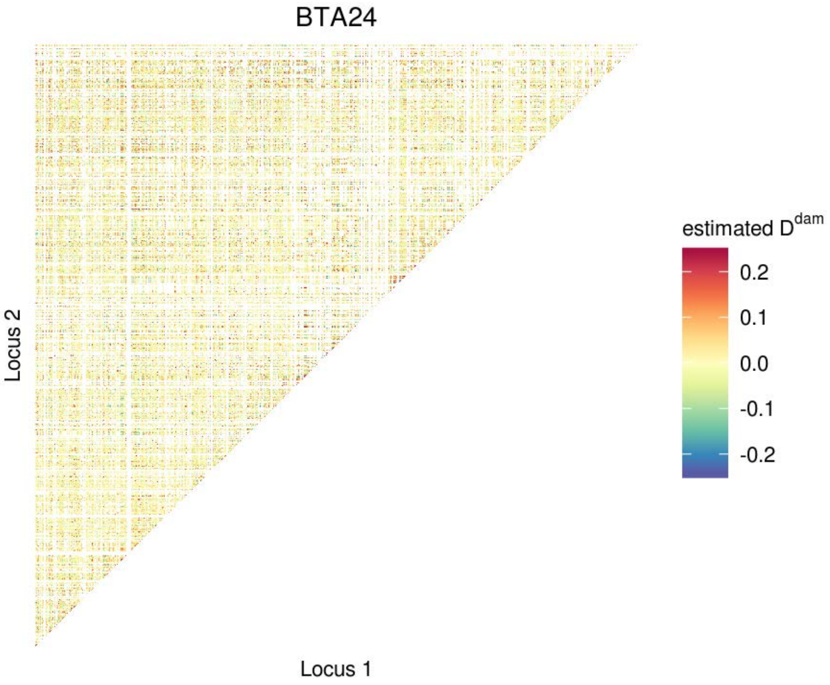

BTA24
Locus 2
Locus 1
estimated D^{dam}
0.2
0.1
0.0
-0.1
-0.2

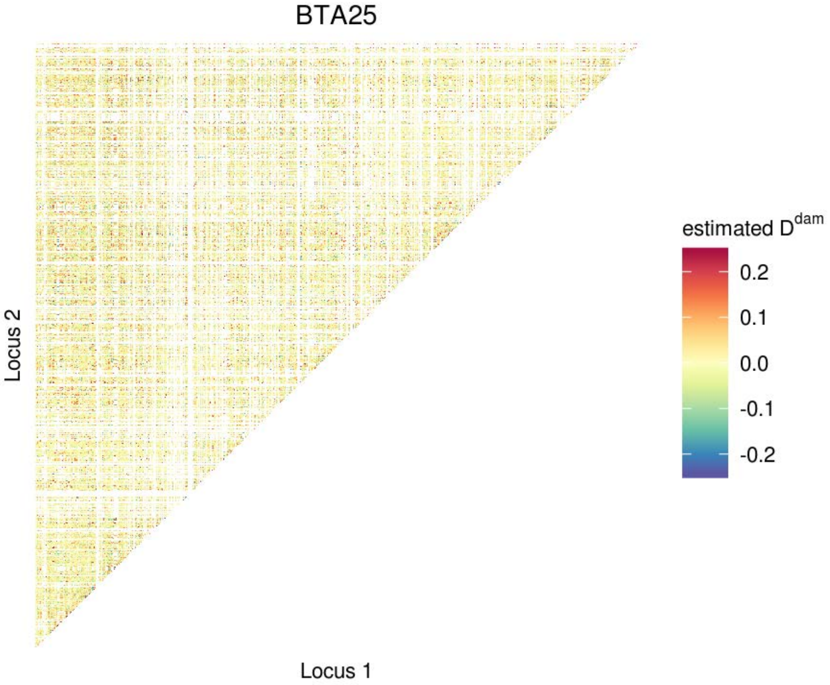

BTA25
estimated D^{dam}
0.2
0.1
0.0
-0.1
-0.2
Locus 2
Locus 1

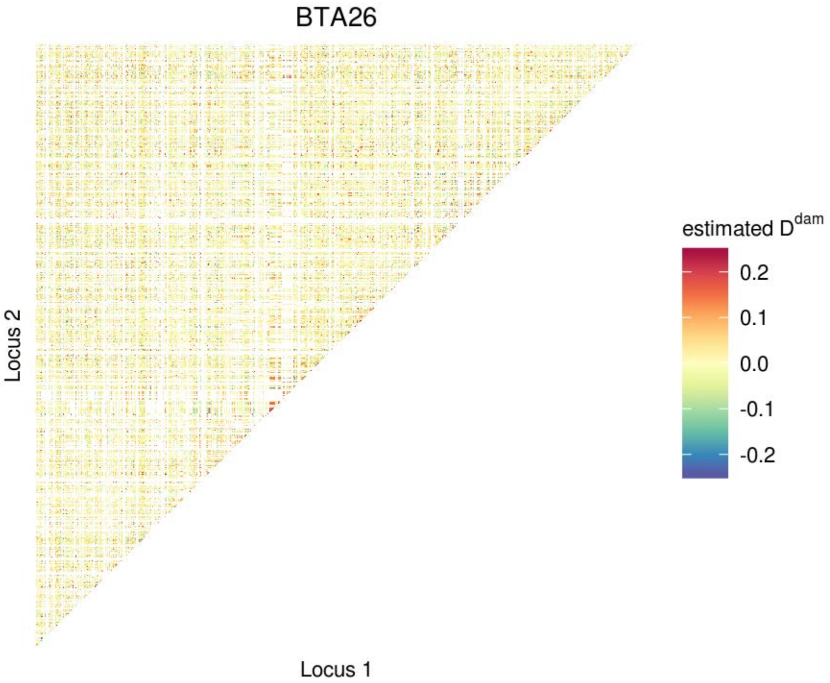
BTA26
Locus 2
Locus 1
estimated D^{dam}
0.2
0.1
0.0
-0.1
-0.2

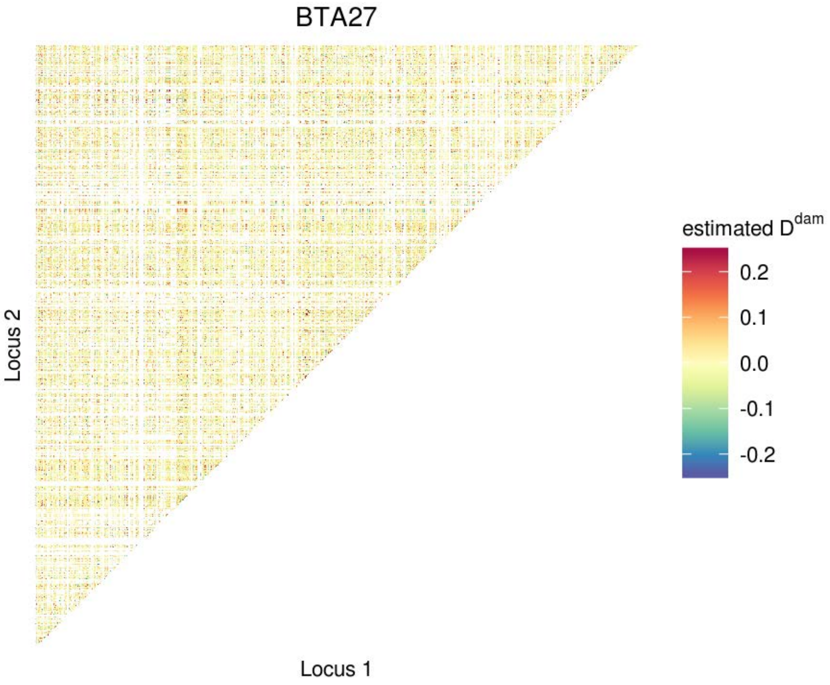
BTA27
Locus 2
Locus 1
estimated D^dam
0.2
0.1
0.0
-0.1
-0.2

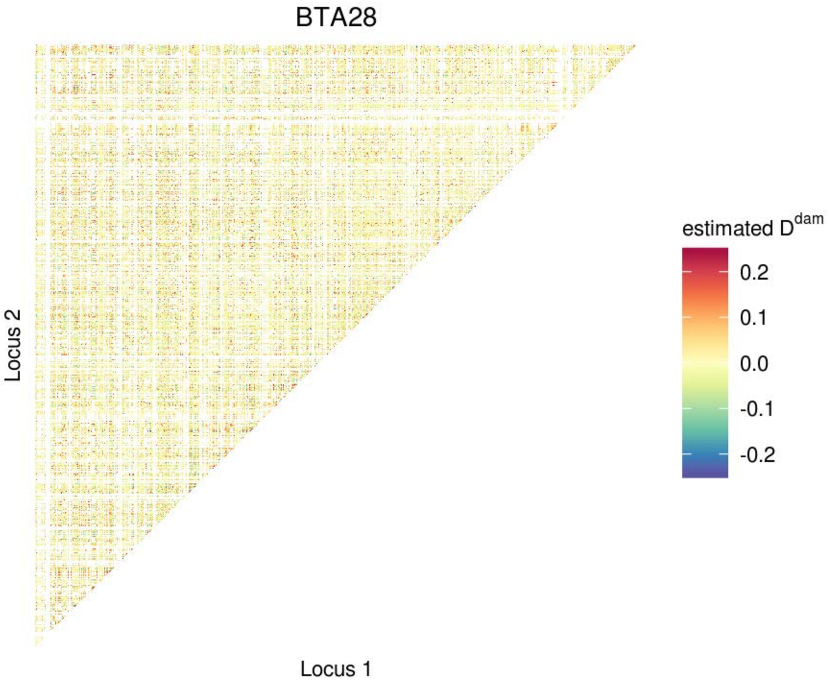

BTA28
Locus 2
Locus 1
estimated D^{dam}
0.2
0.1
0.0
-0.1
-0.2

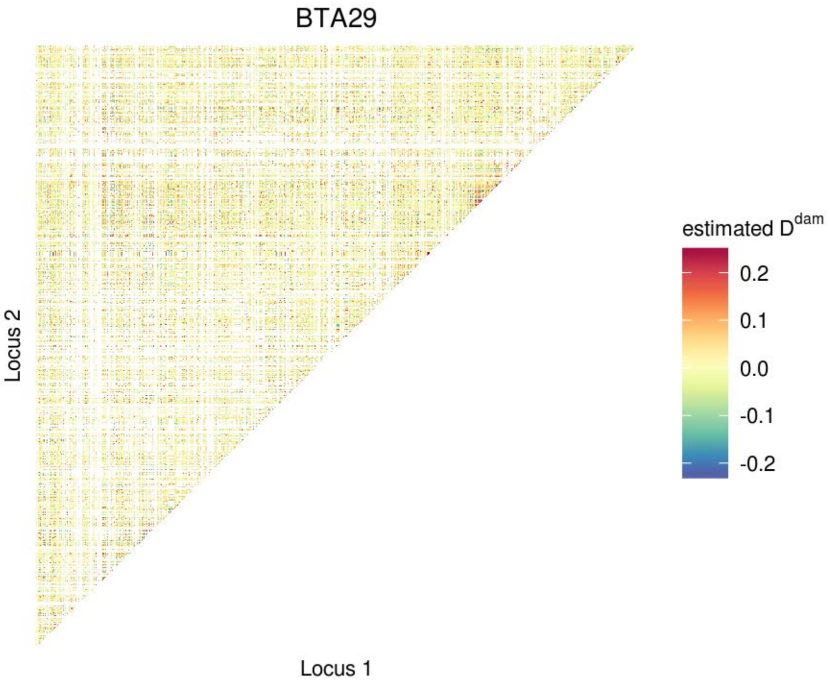
BTA29
Locus 2
Locus 1
estimated D^{dam}
0.2
0.1
0.0
-0.1
-0.2
