# Supplementary material for: Estimation of Recombination Rate and Maternal Linkage Disequilibrium in Half-Sibs
Source: Front Genet. 2018 Jun 5;9:186. doi: 10.3389/fgene.2018.00186 (PMC5996054; doi:10.3389/fgene.2018.00186)
Supplement: Figure S4 — Estimates of r2 for all autosomes using empirical bovine data. Pairwise r2's were obtained using the stepwise procedure EMDP. [file Image_4.PDF]

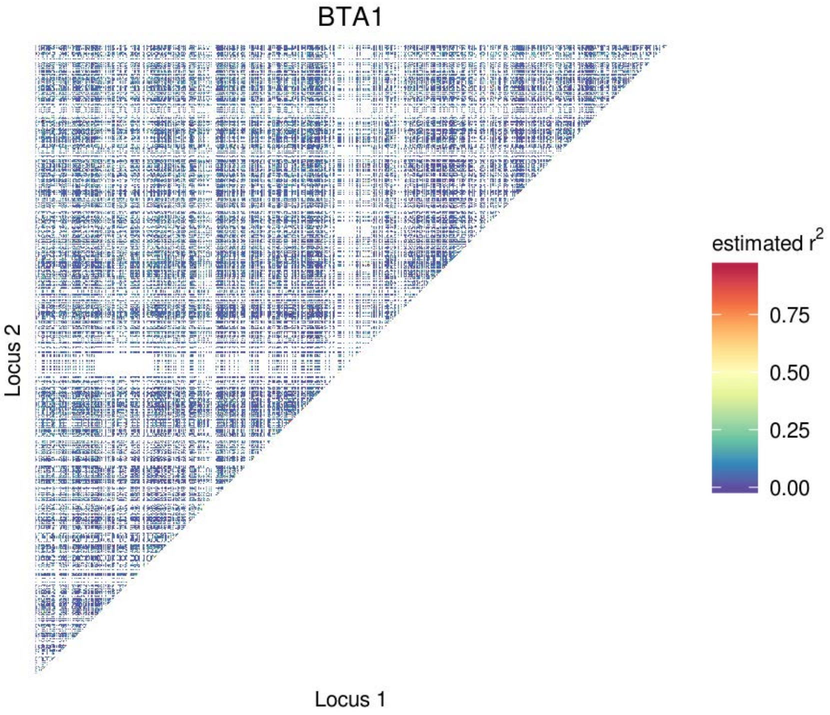
BTA1
Locus 2
Locus 1
estimated r2
0.75
0.50
0.25
0.00

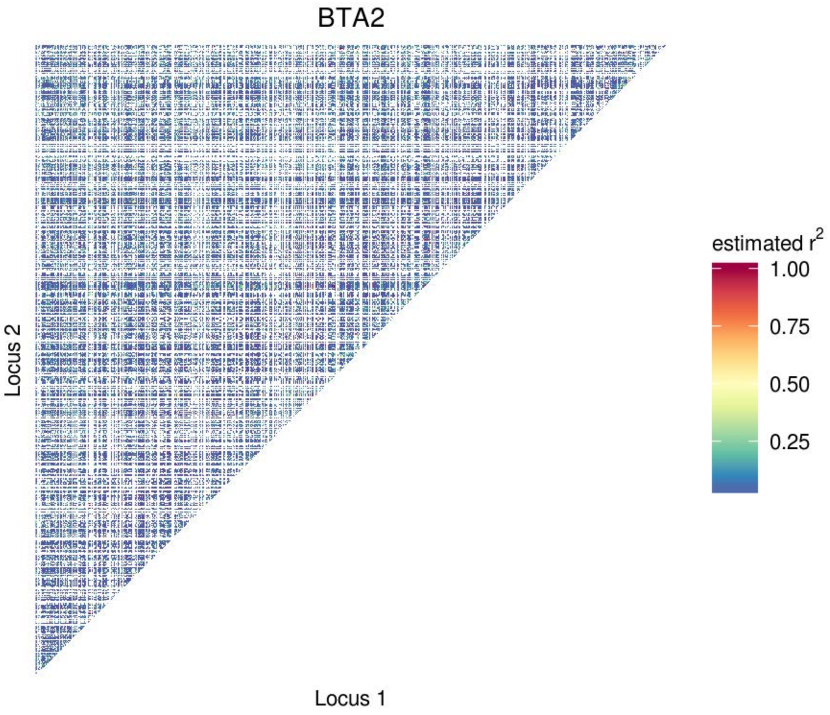
BTA2
Locus 2
Locus 1
estimated r^2
1.00
0.75
0.50
0.25

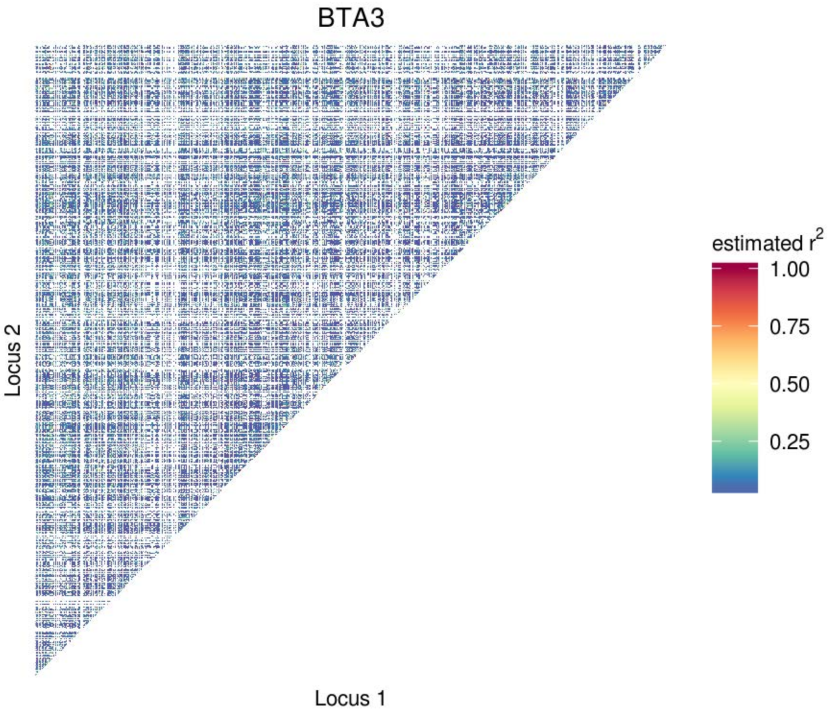
BTA3
Locus 2
Locus 1
estimated r^2
1.00
0.75
0.50
0.25

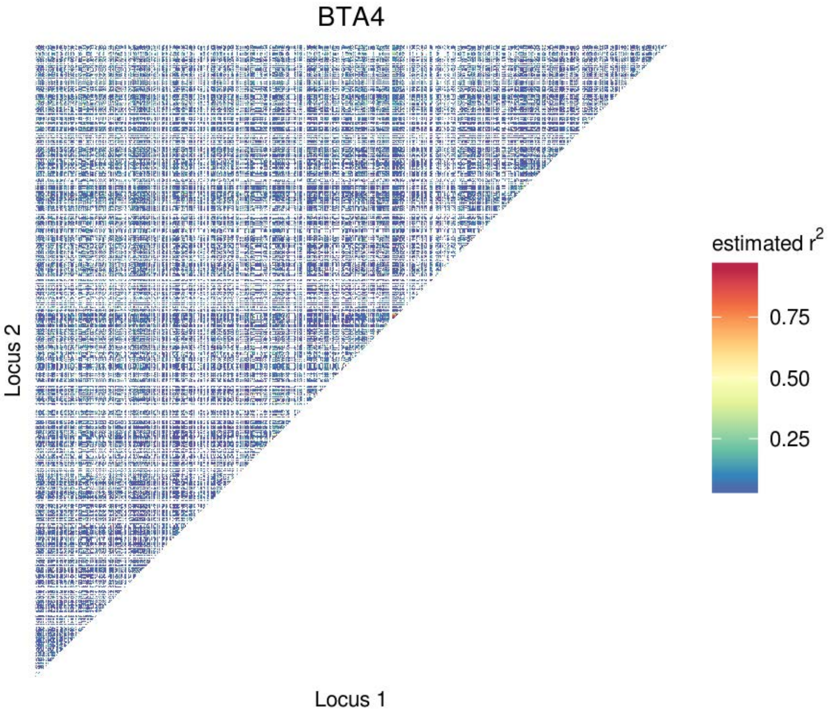
BTA4
Locus 2
Locus 1
estimated r^2
0.75
0.50
0.25

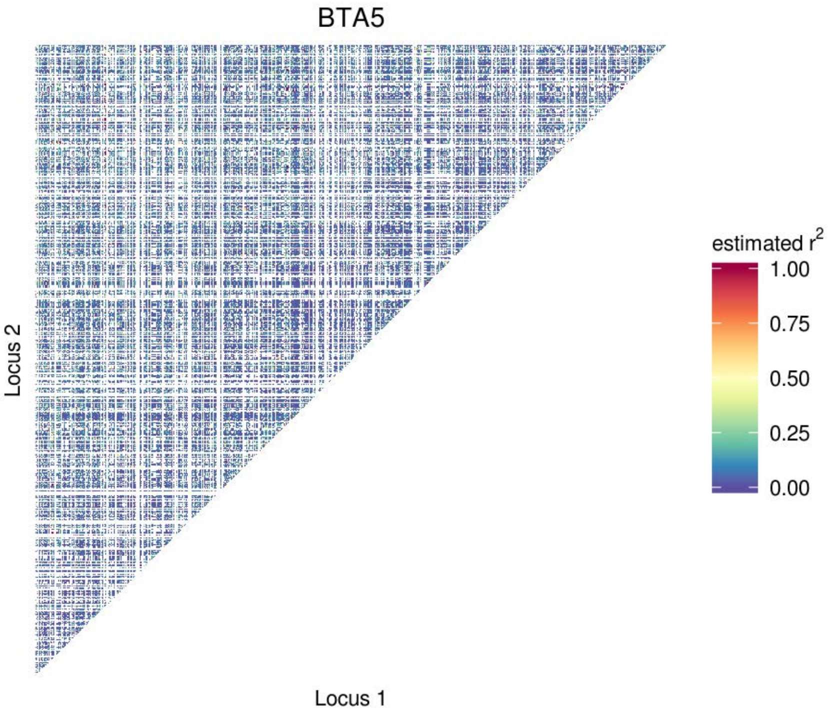
BTA5
Locus 2
Locus 1
estimated r2
1.00
0.75
0.50
0.25
0.00

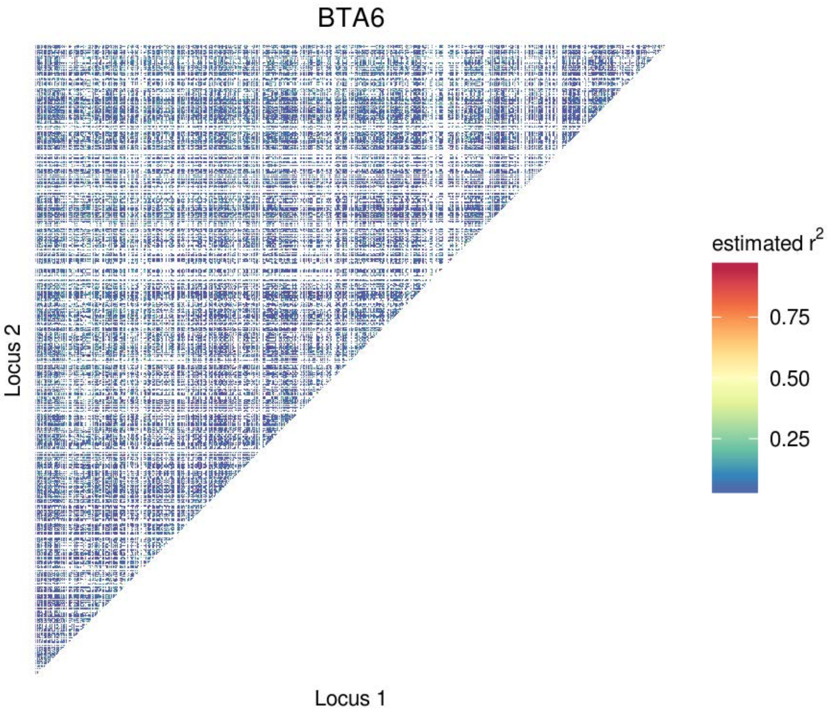
BTA6
Locus 2
Locus 1
estimated r2
0.75
0.50
0.25

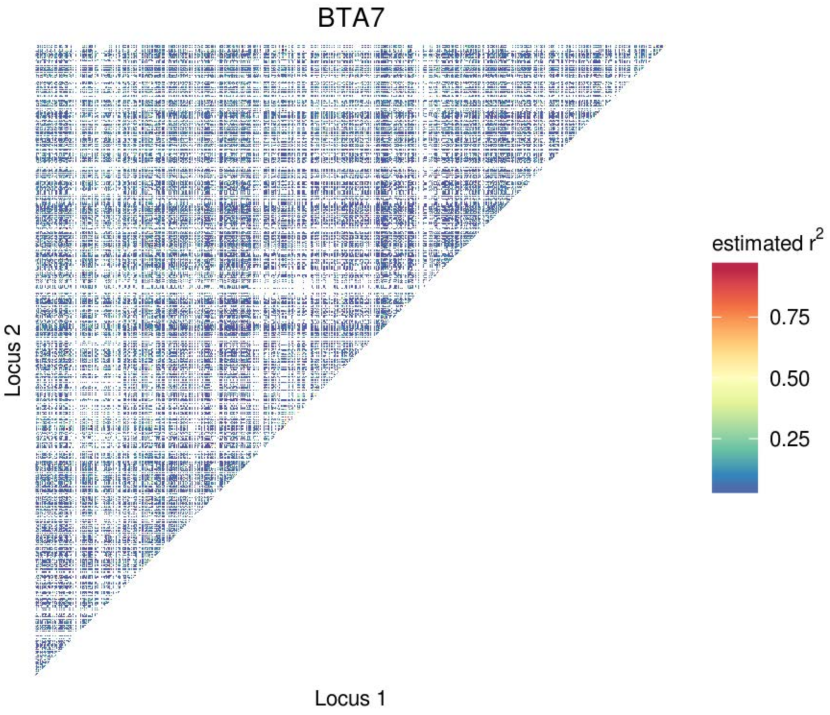

BTA7
Locus 2
Locus 1
estimated r²
0.75
0.50
0.25

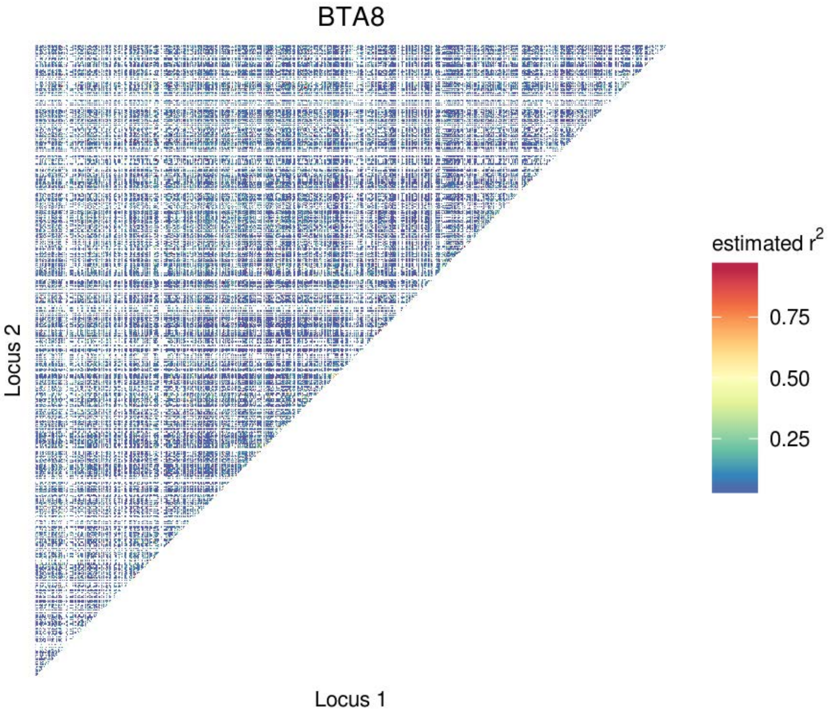

BTA8
Locus 2
Locus 1
estimated r2
0.75
0.50
0.25

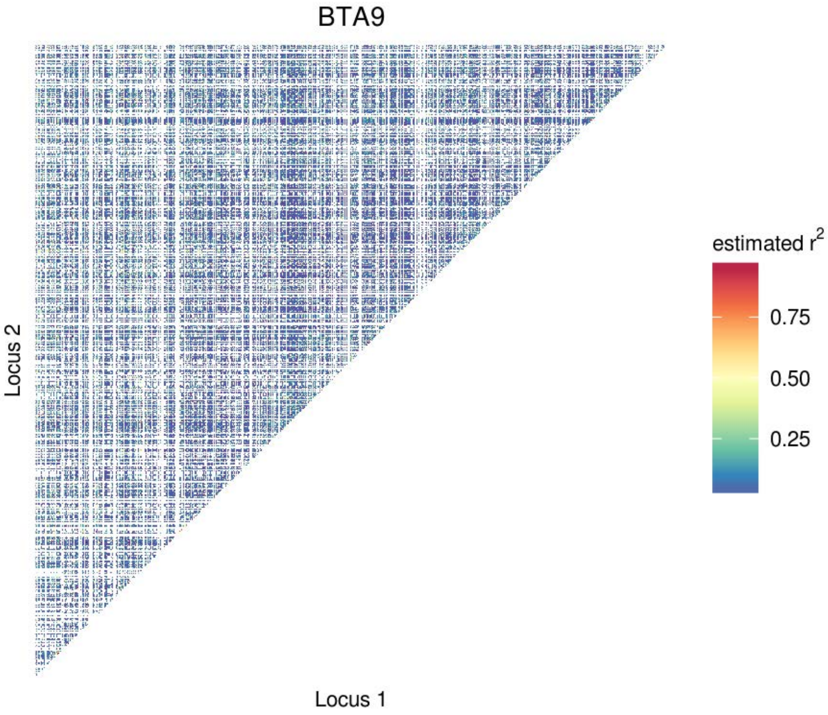
BTA9
estimated r^2
0.75
0.50
0.25
Locus 2
Locus 1

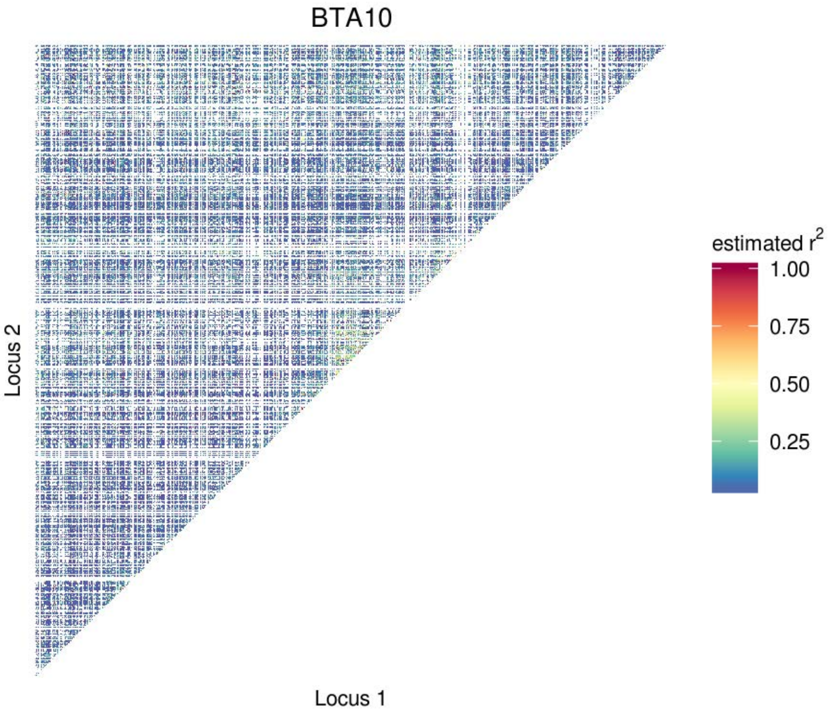
BTA10
Locus 2
Locus 1
estimated r^2
1.00
0.75
0.50
0.25

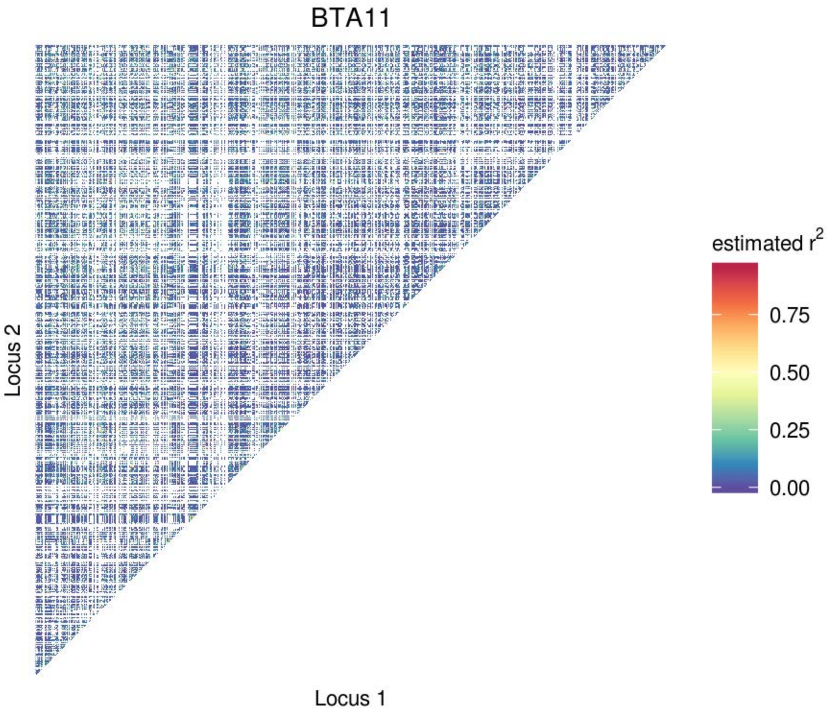
BTA11
Locus 2
Locus 1
estimated r2
0.75
0.50
0.25
0.00

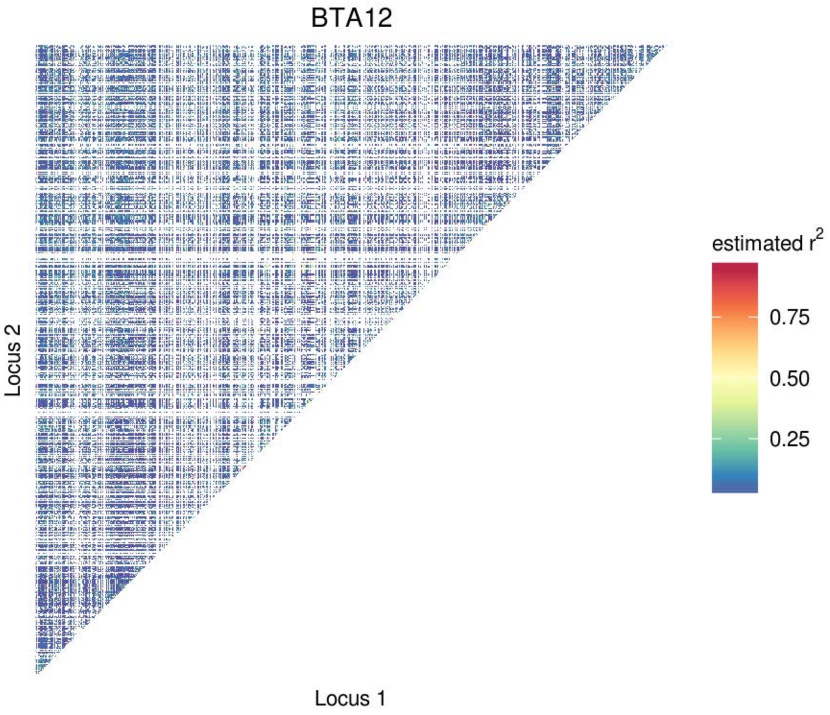
BTA12
estimated r^2
0.75
0.50
0.25
Locus 2
Locus 1

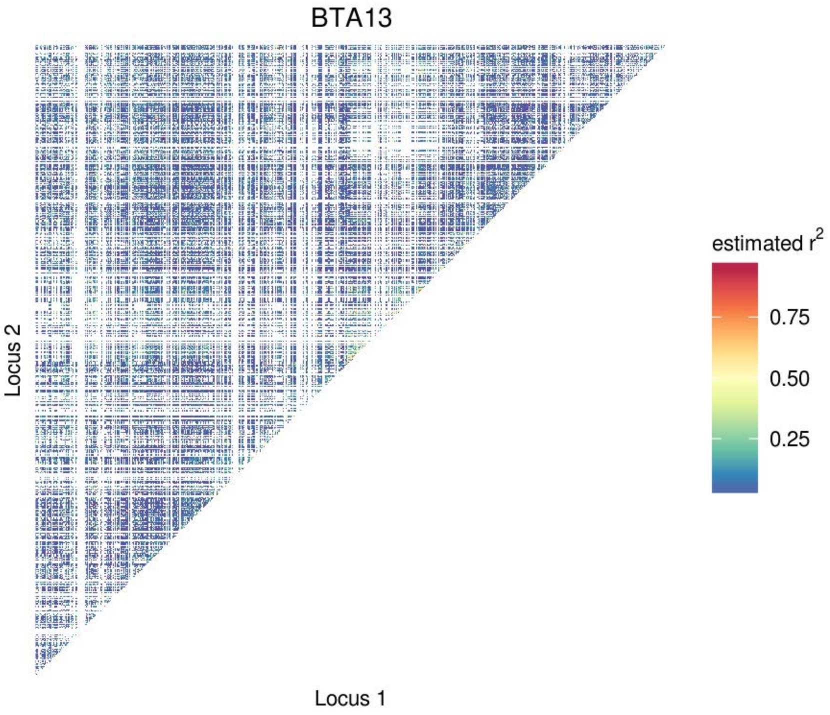

BTA13
Locus 2
Locus 1
estimated r^2
0.75
0.50
0.25

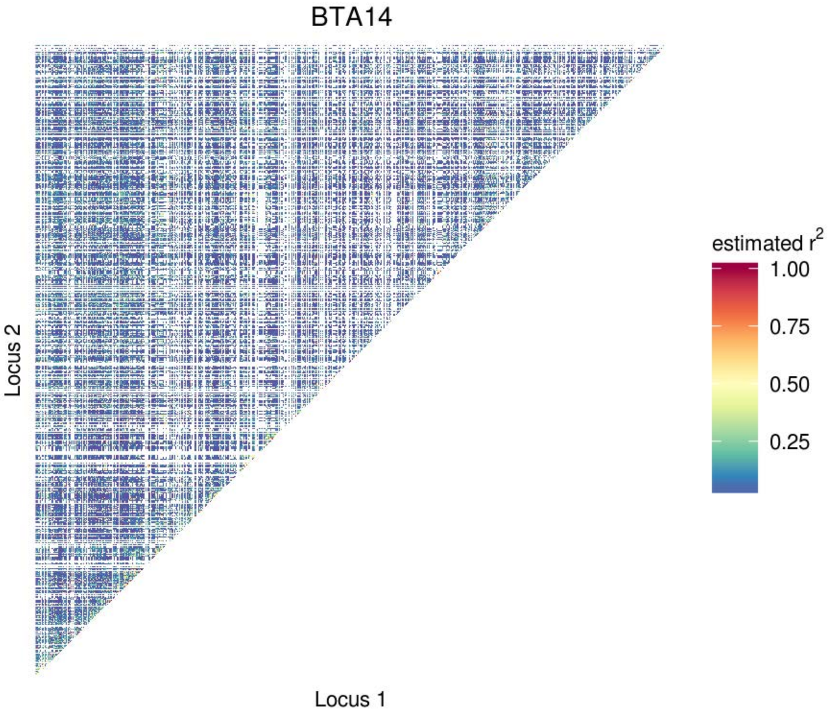
BTA14
Locus 2
Locus 1
estimated r2
1.00
0.75
0.50
0.25

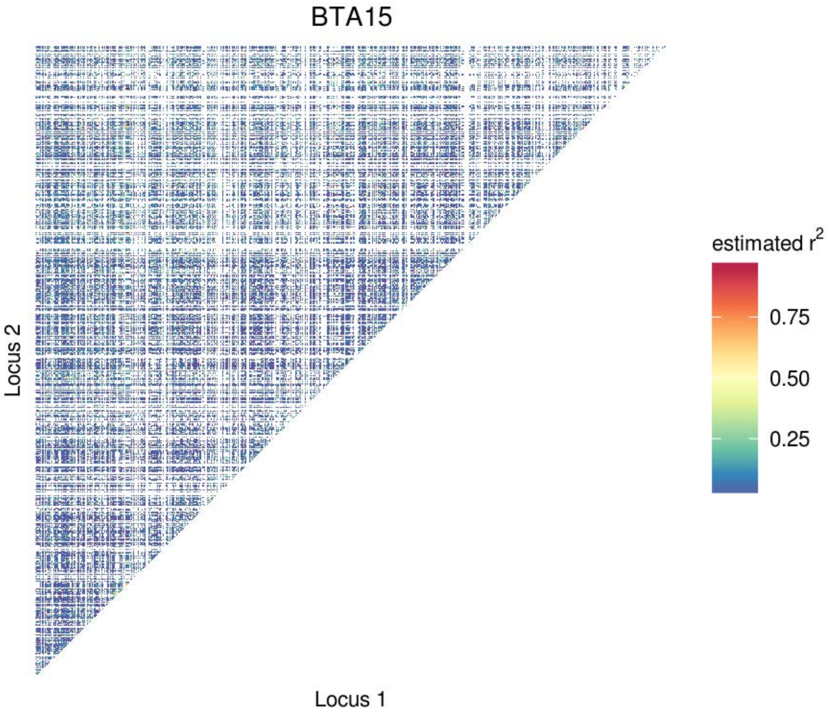

BTA15
Locus 2
Locus 1
estimated r^2
0.75
0.50
0.25

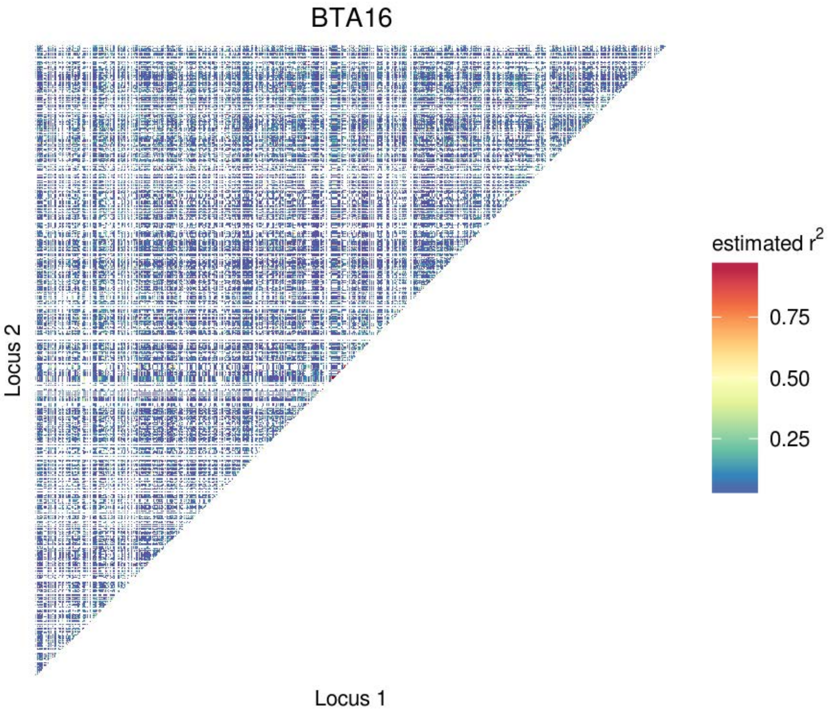

BTA16
Locus 2
Locus 1
estimated r2
0.75
0.50
0.25

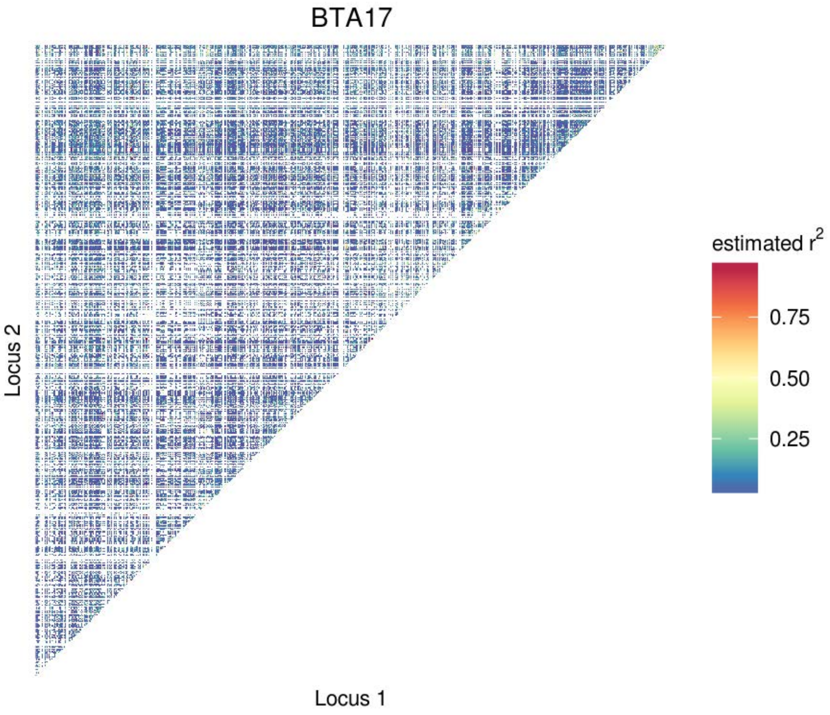

BTA17
Locus 2
Locus 1
estimated r2
0.75
0.50
0.25

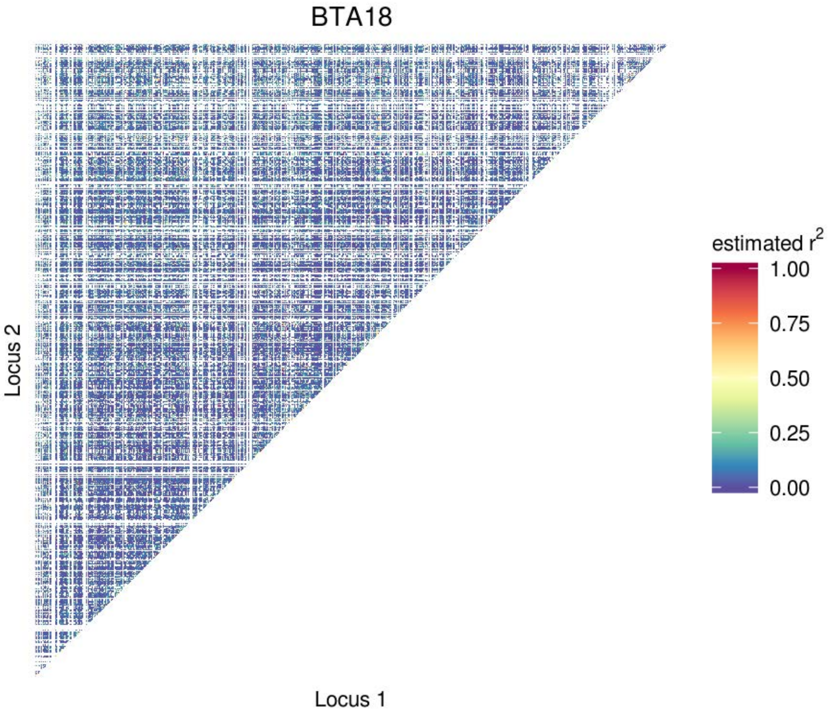

BTA18
Locus 2
Locus 1
estimated r²
1.00
0.75
0.50
0.25
0.00

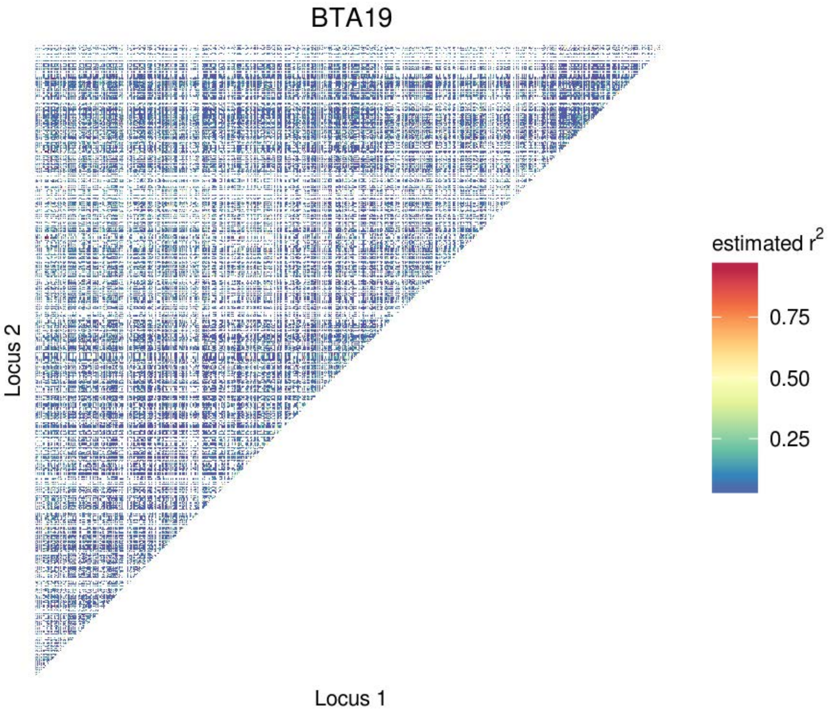
BTA19
Locus 2
Locus 1
estimated r²
0.75
0.50
0.25

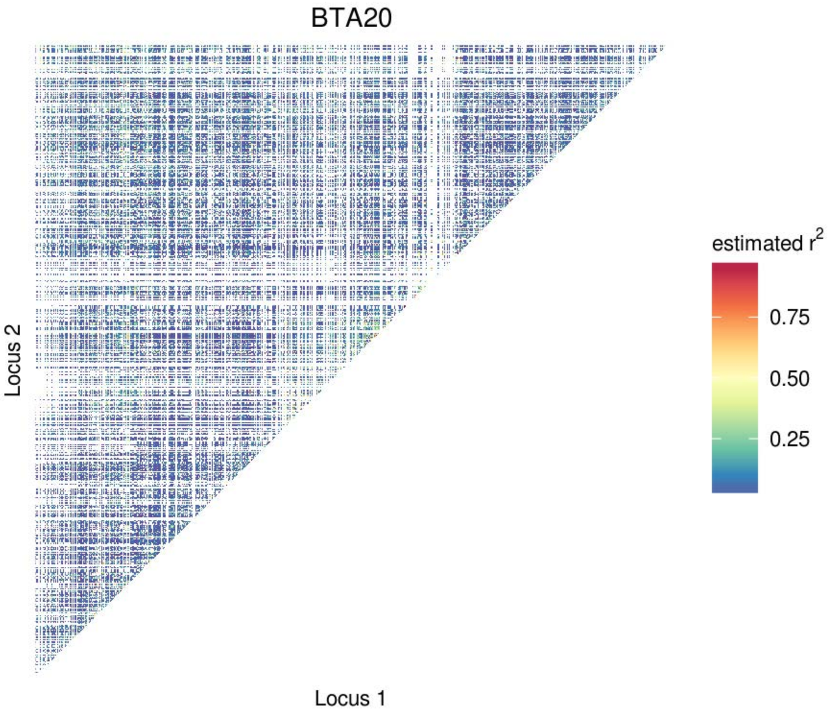
BTA20
estimated r^2
0.75
0.50
0.25
Locus 2
Locus 1

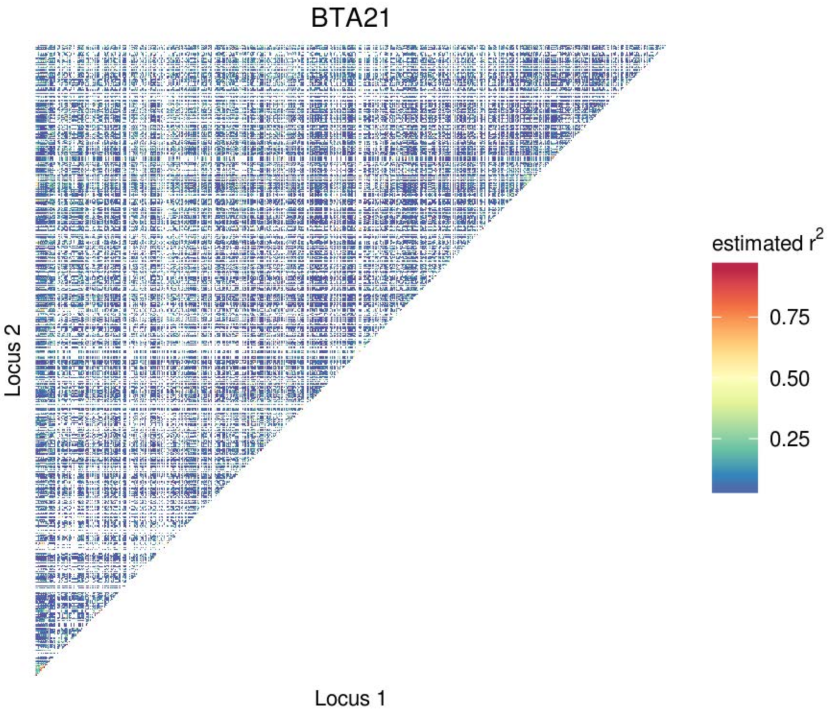
BTA21
Locus 2
Locus 1
estimated r2
0.75
0.50
0.25

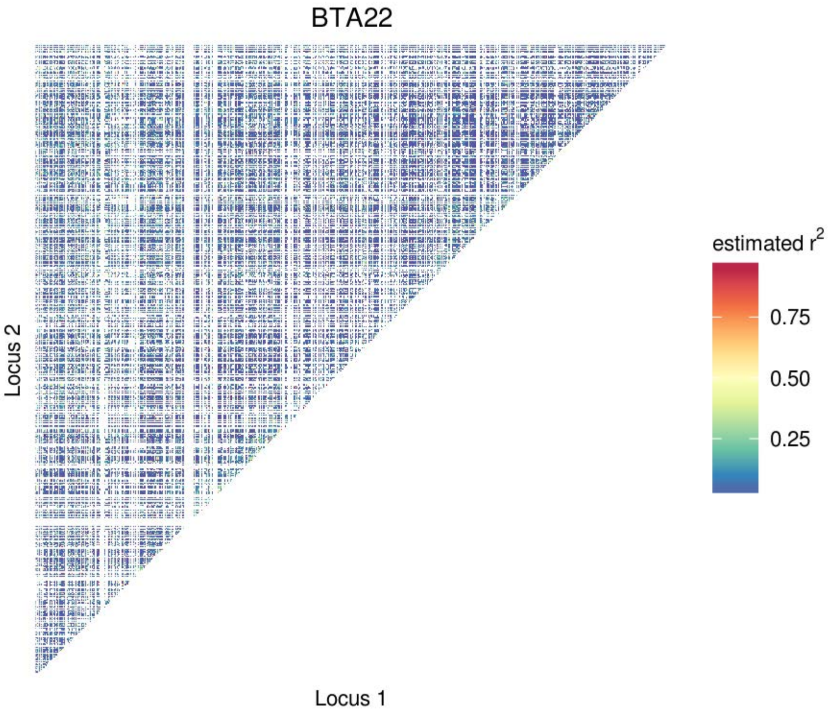
BTA22
Locus 2
Locus 1
estimated r^2
0.75
0.50
0.25

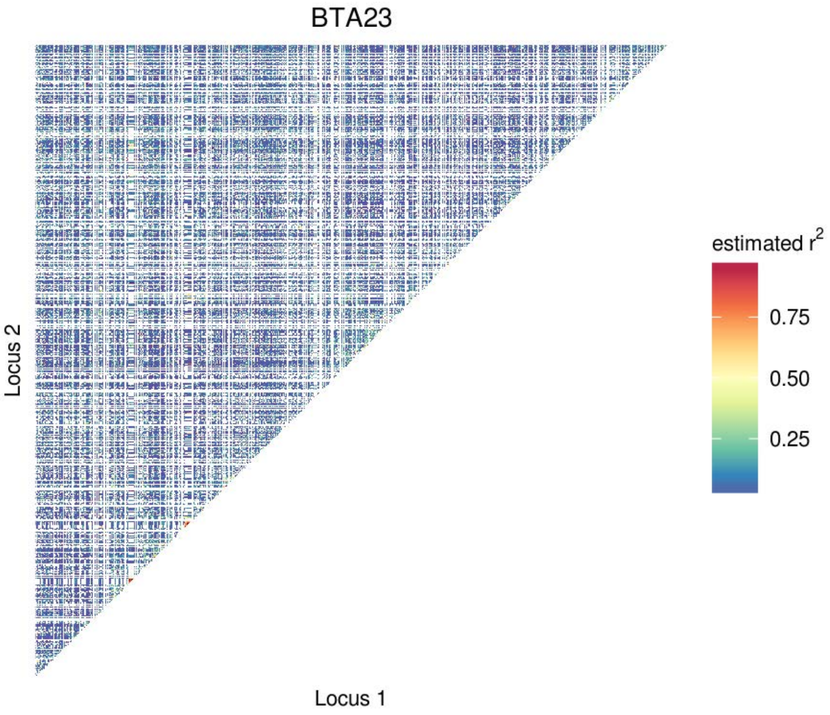

BTA23
Locus 2
Locus 1
estimated r2
0.75
0.50
0.25

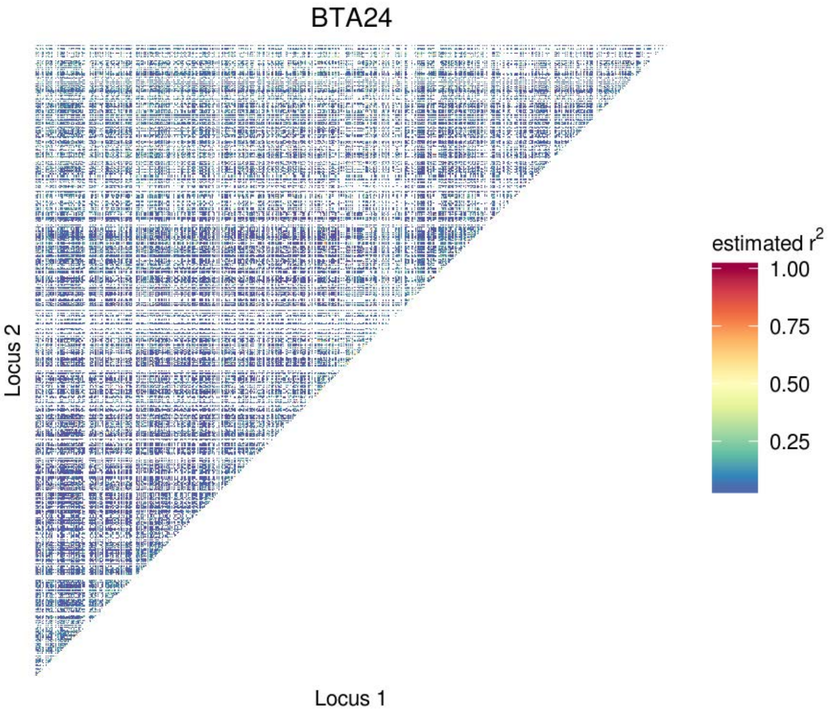
BTA24
Locus 2
Locus 1
estimated r^2
1.00
0.75
0.50
0.25

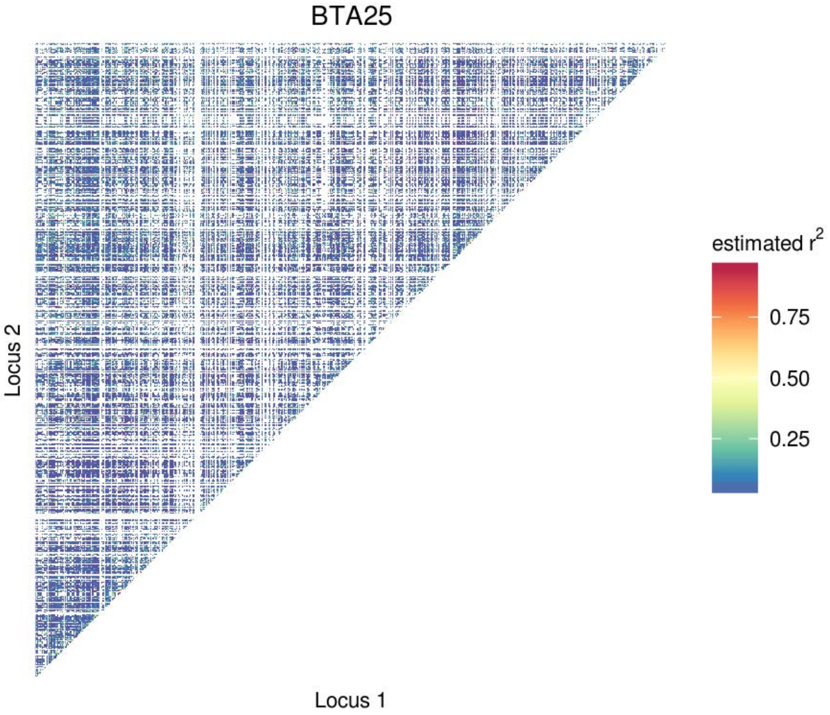
BTA25
Locus 2
Locus 1
estimated r^2
0.75
0.50
0.25

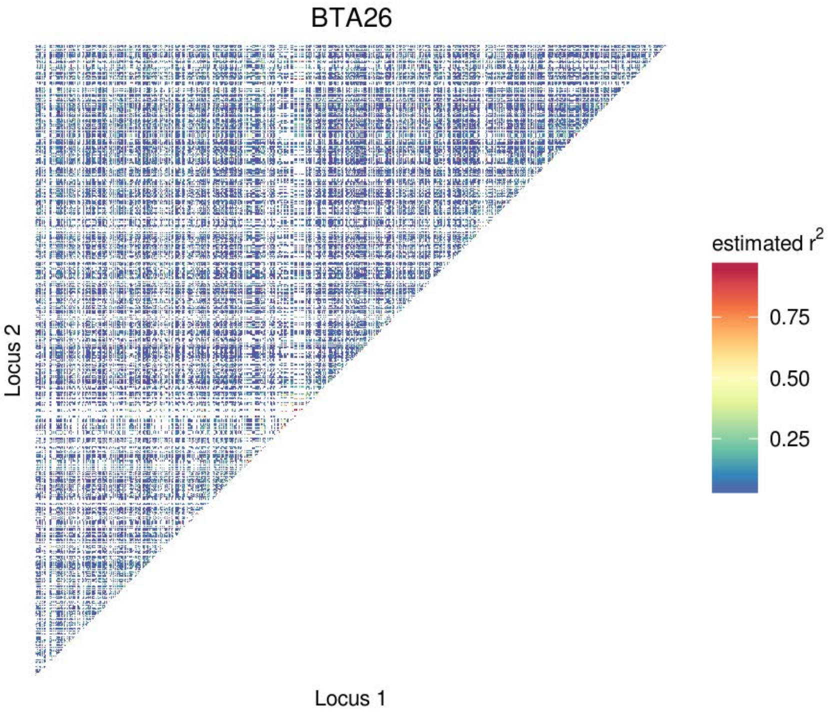
BTA26
estimated r^2
0.75
0.50
0.25
Locus 2
Locus 1

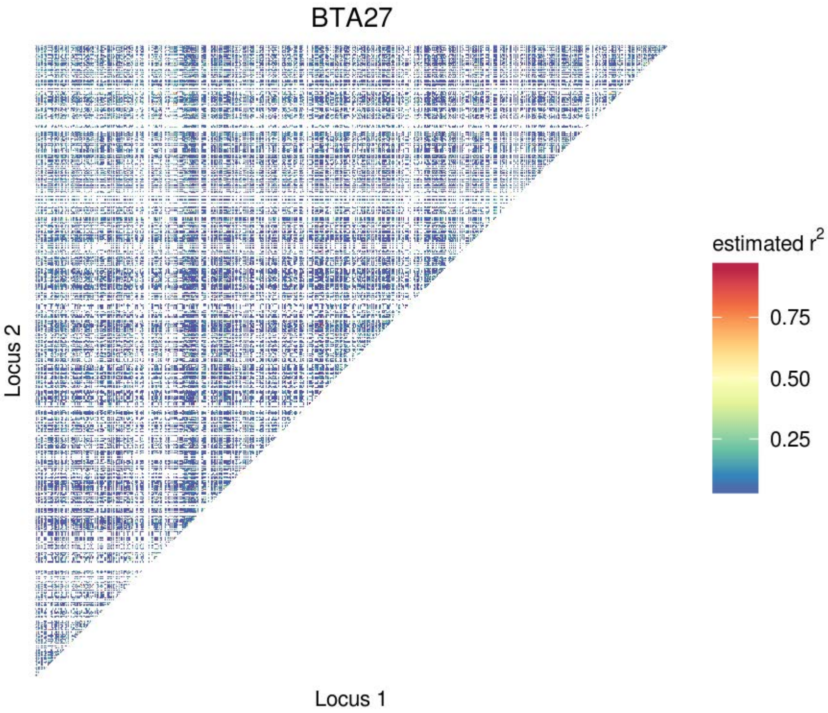
BTA27
Locus 2
Locus 1
estimated r²
0.75
0.50
0.25

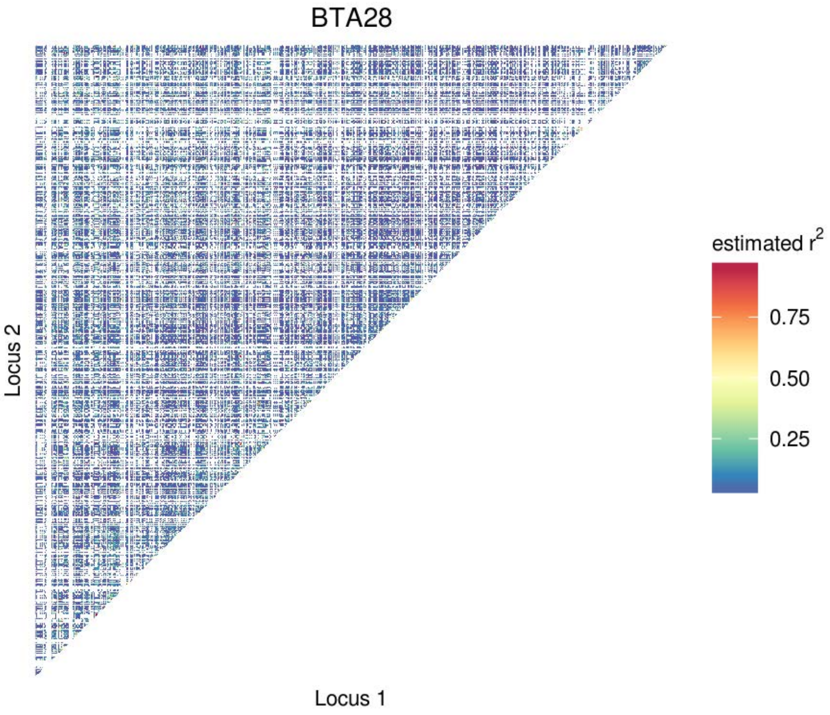
BTA28
Locus 2
Locus 1
estimated r²
0.75
0.50
0.25

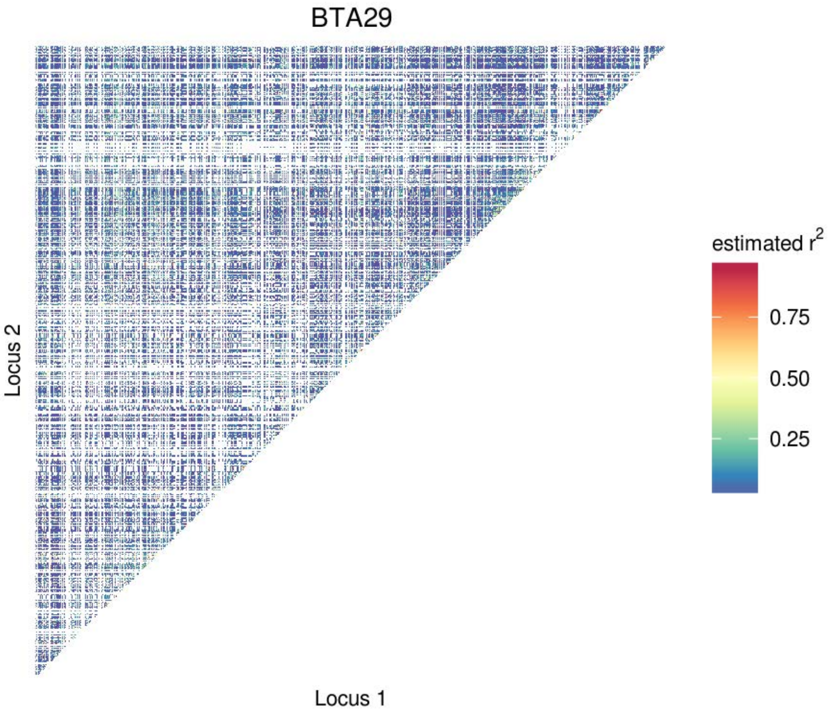
BTA29
Locus 2
Locus 1
estimated r^2
0.75
0.50
0.25
