# Supplementary material for: Estimation of Recombination Rate and Maternal Linkage Disequilibrium in Half-Sibs
Source: Front Genet. 2018 Jun 5;9:186. doi: 10.3389/fgene.2018.00186 (PMC5996054; doi:10.3389/fgene.2018.00186)
Supplement: Table S2 — MSE of estimated paternal recombination rate and maternal linkage disequilibrium for simulated scenarios. Start values were fixed (EMT0 and EMT05) or adapted (EMDP), 1,000 half-sibs were simulated with 10,000 replicates. [file Table_2.DOCX]

| **MSE $\hat{\theta}$** | | | | | |  | **MSE $\hat{D}^{dam}$** | | | | |
| --- | --- | --- | --- | --- | --- | --- | --- | --- | --- | --- | --- |
|  |  | **$p_{1}=p_{2}=0.5$** | **$p_{1}=p_{2}=0.4$** | **$\begin{matrix} p_{1}=0.4 \\ p_{2}=0.6 \end{matrix}$** | **$p_{1}=p_{2}=0.8$** |  |  | **$p_{1}=p_{2}=0.5$** | **$p_{1}=p_{2}=0.4$** | **$\begin{matrix} p_{1}=0.4 \\ p_{2}=0.6 \end{matrix}$** | **$p_{1}=p_{2}=0.8$** |
| **θ=0.01 D^dam^=0.05** | **EMT05** | 0.063 | 0.001 | **0.000** | **0.000** |  | **EMT05** | 0.016 | **0.000** | **0.000** | **0.000** |
|  | **EMT0** | **0.000** | **0.000** | **0.000** | **0.000** |  | **EMT0** | **0.000** | **0.000** | **0.000** | **0.000** |
|  | **EMDP** | 0.029 | 0.001 | **0.000** | **0.000** |  | **EMDP** | 0.007 | **0.000** | **0.000** | **0.000** |
|  |  |  |  |  |  |  |  |  |  |  |  |
| **θ=0.20 D^dam^=0.05** | **EMT05** | 0.021 | 0.005 | 0.003 | **0.001** |  | **EMT05** | 0.005 | **0.001** | 0.001 | **0.000** |
|  | **EMT0** | **0.003** | **0.003** | **0.002** | **0.001** |  | **EMT0** | **0.001** | **0.001** | **0.000** | **0.000** |
|  | **EMDP** | 0.021 | 0.006 | **0.002** | **0.001** |  | **EMDP** | 0.005 | **0.001** | **0.000** | **0.000** |
|  |  |  |  |  |  |  |  |  |  |  |  |
| **θ=0.40 D^dam^=0.15** | **EMT05** | 0.022 | 0.006 | **0.001** | **0.000** |  | **EMT05** | **0.005** | 0.002 | **0.000** | **0.000** |
|  | **EMT0** | 0.039 | 0.022 | 0.006 | 0.003 |  | **EMT0** | 0.010 | 0.005 | 0.001 | **0.000** |
|  | **EMDP** | **0.021** | **0.005** | 0.004 | 0.003 |  | **EMDP** | **0.005** | **0.001** | 0.001 | **0.000** |
|  |  |  |  |  |  |  |  |  |  |  |  |
