# Supplementary material for: Estimation of Recombination Rate and Maternal Linkage Disequilibrium in Half-Sibs
Source: Front Genet. 2018 Jun 5;9:186. doi: 10.3389/fgene.2018.00186 (PMC5996054; doi:10.3389/fgene.2018.00186)
Supplement: File S1 — Investigations on the likelihood function and derivation of the Hessian matrix. [file Data_Sheet_1.pdf]

Supplemental Material

The Likelihood function

Although, the form of the likelihood function (2) is in itself elementary, computing a maximum likelihood estimation is a task not obvious. This will be explained in detail in what follows.

The structure of each parenthesis of equation (2) allows a symbolical simplification of the form

$$f_i(D^{sire}, D^{dam}, p_1, p_2) = a_i D^{sire} D^{dam} + b_i(p_1, p_2) D^{sire} + c_i D^{dam} + d_i(p_1, p_2), \quad i = 1, 2, \ldots, 9 \tag{S1}$$

given that a_i and c_i are constants, whereas both coefficients b_i and d_i are functions of the parameters p_1 and p_2. Their values and parametrization are summarized in Table S1.

Table S1: Values for the coefficients in (S1)

i	a_i	b_i	c_i	d_i
1	1	$(1-p_1)(1-p_2)$	0.25	$0.25(1-p_1)(1-p_2)$
2	-2	$-(1-p_1)(1-2p_2)$	0	$0.25(1-p_1)$
3	1	$-(1-p_1)p_2$	-0.25	$0.25(1-p_1)p_2$
4	-2	$-(1-2p_1)(1-p_2)$	0	$0.25(1-p_2)$
5	4	$(1-2p_1)(1-2p_2)$	0	0.25
6	-2	$(1-2p_1)p_2$	0	$0.25p_2$
7	1	$-p_1(1-p_2)$	-0.25	$0.25p_1(1-p_2)$
8	-2	$p_1(1-2p_2)$	0	$0.25p_1$
9	1	p_1p_2	0.25	$0.25p_1p_2$
	$\sum a_i = 0$	$\sum b_i = 0$	$\sum c_i = 0$	$\sum d_i = 1$

Based on (S1), the Likelihood function (2) can be written as (omitting constants)

$$LF(D^{sire}, D^{dam}, p_1, p_2 \,|\, n_i) = \prod_{i=1}^{9} \Big(f_i(D^{sire}, D^{dam}, p_1, p_2) \Big)^{n_i}, \tag{S2}$$

where n_i denote the observed frequencies (their correspondence according to genotype is displayed in Table S2).

Table S2: Correspondence for n_i's in (S2), (S3) according to genotype.

1	2	3	4	5	6	7	8	9
BB,BB	BB,AB	BB,AA	AB,BB	AB,AB	AB,AA	AA,BB	AA,AB	AA,AA

Employing (S2) favors an extended manipulation of equation (2). In particular, the logarithmic Likelihood function corresponding to (2) reads as

$$\ln LF(D^{sire}, D^{dam}, p_1, p_2 \,|\, n_i) = \sum_{i=1}^{9} n_i \, \ln f_i, \tag{S3}$$

provided that $f_i > 0$ for *each* i. We note that only for $i = 1$ and 9 the corresponding logarithms are positive by default. For the remaining, proper intervals must be chosen. Moreover, $\sum_{i=1}^{9} f_i = 1$ which is easily demonstrated utilizing the last line of Table S1.

Optimization

Obtaining extrema in the classic framework of calculus requires the application of the derivative test. Recall that the logarithm is a monotonic transformation preserving the locations of possible extrema. Therefore, it is irrelevant if one utilizes (S2) or (S3) for the acquisition of the critical points. However, working with factored products as present in (S2) is simpler as working with series, by virtue of

$$\frac{\partial LF(\boldsymbol{\vartheta})}{\partial \vartheta_j} = \left(\mathbf{n}^\top \mathbf{F}_{\vartheta_j}\right) \prod_{i=1}^{M} \left(f_i(\boldsymbol{\vartheta})\right)^{n_i}, \tag{S4}$$

where $\boldsymbol{\vartheta}$ is a generic K-dimensional vector with entries ϑ_j, $j \in \{1, \dots, K\}$, whereas

$$\mathbf{F}_{\vartheta_j} = \begin{pmatrix} \frac{1}{f_1(\boldsymbol{\vartheta})} \frac{\partial f_1(\boldsymbol{\vartheta})}{\partial \vartheta_j} \\ \vdots \\ \frac{1}{f_M(\boldsymbol{\vartheta})} \frac{\partial f_M(\boldsymbol{\vartheta})}{\partial \vartheta_j} \end{pmatrix} = \frac{\partial}{\partial \vartheta_j} \begin{pmatrix} \ln f_1(\boldsymbol{\vartheta}) \\ \vdots \\ \ln f_M(\boldsymbol{\vartheta}) \end{pmatrix}. \tag{S5}$$

In the instance described in the manuscript, $\boldsymbol{\vartheta} = (D^{sire}\ D^{dam}\ p_1\ p_2)^\top$ and $M = 9$. The corresponding expressions for the derivatives present in (S5) are given in Table S3.

Critical points $\boldsymbol{\vartheta}^*$ are evaluated by equating (S4) to zero, namely $\mathbf{n}^\top \mathbf{F}_{\vartheta_j} = 0$, yielding 4 highly non-linear equations with 4 unknowns. These equations are in detail,

$$\sum_{i=1}^{9} \frac{n_i}{f_i}(a_i\, D^{dam} + b_i) = 0,\ \sum_{i=1}^{9} \frac{n_i}{f_i}(a_i\, D^{sire} + c_i) = 0,\ \sum_{i=1}^{9} \frac{n_i}{f_i}(\partial_{p_k} b_i\, D^{sire} + \partial_{p_k} d_i) = 0, k = 1, 2. \tag{S6}$$

The instance where $\prod_{i=1}^{9} f_i^{n_i} = 0$ is examined separately.

Table S3: Expressions for the derivatives $\partial_{\vartheta_j} f_i$

i	$\partial_{D^{sire}} f_i$	$\partial_{D^{dam}} f_i$	$\partial_{p_1} f_i$	$\partial_{p_2} f_i$
1	$D^{dam} + (1-p_1)(1-p_2)$	$D^{sire} + 0.25$	$-0.25(1+4D^{sire})(1-p_2)$	$-0.25(1+4D^{sire})(1-p_1)$
2	$-2D^{dam} + (1-p_1)(1-2p_2)$	$-2D^{sire}$	$-0.25 + D^{sire} - 2D^{sire}p_2$	$2D^{sire}(1-p_1)$
3	$D^{dam} - (1-p_1)p_2$	$D^{sire} - 0.25$	$-\left(0.25 - D^{sire}\right) p_2$	$0.25(1-4D^{sire})(1-p_1)$
4	$-2D^{dam} - (1-2p_1)(1-p_2)$	$-2D^{sire}$	$2D^{sire}(1-p_2)$	$-0.25 + D^{sire} - 2D^{sire}p_1$
5	$4D^{dam} + (1-2p_1)(1-2p_2)$	$4D^{sire}$	$-2D^{sire}(1-2p_2)$	$-2D^{sire}(1-2p_1)$
6	$-2D^{dam} + (1-2p_1)p_2$	$-2D^{sire}$	$-2D^{sire}p_2$	$0.25 + D^{sire} - 2D^{sire}p_1$
7	$D^{dam} - (1-p_2)p_1$	$D^{sire} - 0.25$	$0.25(1-4D^{sire})(1-p_2)$	$(D^{sire} - 0.25)p_1$
8	$-2D^{dam} + p_1(1-2p_2)$	$-2D^{sire}$	$0.25 + D^{sire} - 2D^{sire}p_2$	$-2D^{sire}p_1$
9	$D^{dam} + p_1p_2$	$D^{sire} + 0.25$	$\left(0.25 + D^{sire}\right) p_2$	$(D^{sire} + 0.25)p_1$
	$\sum \partial_{D^{sire}} f_i = 0$	$\sum \partial_{D^{dam}} f_i = 0$	$\sum \partial_{p_1} f_i = 0$	$\sum \partial_{p_2} f_i = 0$

The vector $\mathbf{n}$ corresponds to the observed genotype frequencies, thus connected to the measurements, and accordingly, finding $\mathbf{n}$ such that $\mathbf{n} \perp \mathbf{F}_{\vartheta_j}$ is an invalid statistical statement. Therefore, it must $\mathbf{F}_{\vartheta_j} = \mathbf{0}$. A straightforward scenario for the latter implies solutions constrained about the boundary of the four-dimensional solution space. These are, $\partial_{\vartheta_j} f_i = 0$ or/and f_i very large, so that $(f_i)^{-1}$ vanishes for *specific* i's. The latter or/and requirement is justified as follows.

The solution space regarding D^{sire} and D^{dam} is restricted as $D^{sire} \in [0, 0.25]$ as well as $D^{dam} \in [L_1, L_2]$, where $L_1 = \max\{-p_1p_2, -(1-p_1)(1-p_2)\}$, $L_2 = \min\{p_1(1-p_2), (1-p_1)p_2\}$. It is apparent that the imposed constrains put restrictions on the scenario where $\partial_{\vartheta_j} f_i = 0$, since a number of derivatives involve the term $D^{sire} + 0.25$ (see Table S3), which does not vanish in the given interval.

On the other hand, solutions $\boldsymbol{\vartheta}^*$ in the interior of the solution space must be obtained by synchronously solving equations (S6), thus taking into consideration the data.

Recognizing the identity (minimum, maximum, saddle) of $\boldsymbol{\vartheta}^*$'s, the Hessian matrix

$$\mathcal{H}_{k,j} = \frac{\partial^2 LF}{\partial \vartheta_k \partial \vartheta_j}, \quad 1 \leq k, j \leq 4, \tag{S7}$$

is computed, where

$$\frac{\partial^2 LF}{\partial \vartheta_k \partial \vartheta_j} = \left\{ \sum_{i=1}^{9} \frac{n_i}{f_i} \left(\frac{\partial^2 f_i}{\partial \vartheta_k \partial \vartheta_j} - \frac{1}{f_i} \frac{\partial f_i}{\partial \vartheta_k} \frac{\partial f_i}{\partial \vartheta_k} \right) + \left(\sum_{i=1}^{9} \frac{n_i}{f_i} \frac{\partial f_i}{\partial \vartheta_k} \right) \left(\sum_{i=1}^{9} \frac{n_i}{f_i} \frac{\partial f_i}{\partial \vartheta_j} \right) \right\} \prod_{i=1}^{9} f_i^{n_i}. \tag{S8}$$

It is evident from (S8) that $\mathcal{H}_{k,j} = 0$, $1 \leq k, j \leq 4$, if evaluated at the roots of f_i, provided that $n_i > 2$, and thus the second derivative test is inconclusive.

Working with the logarithmic likelihood instead, yields

$$\mathcal{H}_{k,j} = \frac{\partial^2 \ln LF}{\partial \vartheta_k \partial \vartheta_j} = \mathbf{n}^\top \, \mathbf{F}_{\vartheta_k \vartheta_j}, \quad 1 \le k, j \le 4, \tag{S9}$$

where, analytically

$$\frac{\partial^2 \ln LF}{\partial \left(D^{sire}\right)^2} = -\sum_{i=1}^{9} n_i f_i^{-2} \Big(a_i \, D^{dam} + b_i \Big)^2 < 0, \tag{S10}$$

$$\frac{\partial^2 \ln LF}{\partial \left(D^{dam}\right)^2} = -\sum_{i=1}^{9} n_i f_i^{-2} \Big(a_i \, D^{sire} + c_i \Big)^2 < 0, \tag{S11}$$

$$\frac{\partial^2 \ln LF}{\partial \left(p_\ell\right)^2} = -\sum_{i=1}^{9} n_i f_i^{-2} \left(\frac{\partial b_i}{\partial p_\ell} \, D^{sire} + \frac{\partial d_i}{\partial p_\ell} \right)^2 < 0, \; \ell = 1, 2 \tag{S12}$$

$$\frac{\partial^2 \ln LF}{\partial D^{sire} \partial D^{dam}} = \sum_{i=1}^{9} n_i \left(f_i(D^{sire}, D^{dam}, p_1, p_2) \right)^{-2} \Big(a_i \, d_i - b_i \, c_i \Big), \tag{S13}$$

$$\begin{aligned} \frac{\partial^2 \ln LF}{\partial p_1 \partial p_2} &= -\sum_{i=1}^{9} n_i f_i^{-2} \prod_{\ell=1}^{2} \left(\frac{\partial b_i}{\partial p_\ell} \, D^{sire} + \frac{\partial d_i}{\partial p_\ell} \right) \\ &\quad + \sum_{i=1}^{9} n_i f_i^{-1} \left(\frac{\partial^2 b_i}{\partial p_1 \partial p_2} \, D^{sire} + \frac{\partial^2 d_i}{\partial p_1 \partial p_2} \right) \end{aligned} \tag{S14}$$

$$\frac{\partial^2 \ln LF}{\partial D^{sire} \partial p_k} = \sum_{i=1}^{9} n_i f_i^{-2} \left[\left(c_i \, D^{dam} + d_i \right) \frac{\partial b_i}{\partial p_\ell} - \left(a_i \, D^{dam} + b_i \right) \frac{\partial d_i}{\partial p_\ell} \right], \tag{S15}$$

$$\frac{\partial^2 \ln LF}{\partial D^{dam} \partial p_\ell} = -\sum_{i=1}^{9} n_i f_i^{-2} \left(a_i \, D^{sire} + c_i \right) \left(\frac{\partial b_i}{\partial p_\ell} D^{sire} + \frac{\partial d_i}{\partial p_\ell} \right), \; \ell = 1, 2. \tag{S16}$$
